# An environmental justice analysis of air pollution in India

**DOI:** 10.1038/s41598-023-43628-3

**Published:** 2023-10-04

**Authors:** Priyanka N. deSouza, Ekta Chaudhary, Sagnik Dey, Soohyeon Ko, Jeremy Németh, Sarath Guttikunda, Sourangsu Chowdhury, Patrick Kinney, S. V. Subramanian, Michelle L. Bell, Rockli Kim

**Affiliations:** 1 Department of Urban and Regional Planning, University of Colorado Denver, Denver, CO USA; 2 Centre for Atmospheric Sciences, Indian Institute of Technology (IIT) Delhi, New Delhi, India; 3 Centre of Excellence for Research on Clean Air, IIT Delhi, New Delhi, India; 4 School of Public Policy, IIT Delhi, New Delhi, India; 5 Department of Public Health Sciences, Graduate School of Korea University, Seoul, South Korea; 6 Interdisciplinary Program in Precision Public Health, Department of Public Health Sciences, Graduate School of Korea University, Seoul, South Korea; 7 Transportation Research and Injury Prevention (TRIP) Centre, Indian Institute of Technology, New Delhi, 110016 India; 8 Urban Emissions, New Delhi, 110019 India; 9 CICERO Center for International Climate Research, Oslo, Norway; 10 School of Public Health, Boston University, Boston, MA USA; 11 Harvard Center for Population and Development Studies, Bow Street, Cambridge, MA 02138 USA; 12 Department of Social and Behavioral Sciences, Harvard T.H. Chan School of Public Health, 677 Huntington Avenue, Boston, MA 02115 USA; 13 School of the Environment, Yale University, New Haven, CT USA; 14 Division of Health Policy and Management, College of Health Science, Korea University, Seoul, South Korea

**Keywords:** Environmental impact, Sustainability

## Abstract

Due to the lack of timely data on socioeconomic factors (SES), little research has evaluated if socially disadvantaged populations are disproportionately exposed to higher PM_2.5_ concentrations in India. We fill this gap by creating a rich dataset of SES parameters for 28,081 clusters (villages in rural India and census-blocks in urban India) from the National Family and Health Survey (NFHS-4) using a precision-weighted methodology that accounts for survey-design. We then evaluated associations between total, anthropogenic and source-specific PM_2.5_ exposures and SES variables using fully-adjusted multilevel models. We observed that SES factors such as caste, religion, poverty, education, and access to various household amenities are important risk factors for PM_2.5_ exposures. For example, we noted that a unit standard deviation increase in the cluster-prevalence of Scheduled Caste and Other Backward Class households was significantly associated with an increase in total-PM_2.5_ levels corresponding to 0.127 μg/m^3^ (95% CI 0.062 μg/m^3^, 0.192 μg/m^3^) and 0.199 μg/m^3^ (95% CI 0.116 μg/m^3^, 0.283 μg/m^3^, respectively. We noted substantial differences when evaluating such associations in urban/rural locations, and when considering source-specific PM_2.5_ exposures, pointing to the need for the conceptualization of a nuanced EJ framework for India that can account for these empirical differences. We also evaluated emerging axes of inequality in India, by reporting associations between recent changes in PM_2.5_ levels and different SES parameters.

## Main

Ambient air pollution is the world’s single largest environmental health risk and is estimated to have been responsible for 6.7 million premature deaths in 2019^1^. Fine particulate matter (PM_2.5_) concentrations in India are among the highest in the world^2–4^. According to the 2019 Global Burden of Disease, PM_2.5_ was estimated to be responsible for 1.67 million deaths (0.98 million deaths from ambient pollution and 0.61 million deaths from household pollution), or 17.8% of the total deaths recorded in India, with economic losses alone corresponding to ~ 1.36% of the India’s Gross Domestic Product in 2017^5^.

In the United States, and elsewhere, a rich body of environmental justice (EJ) research documents the substantial and persistent disparities in exposure to pollution by markers of privilege^6–10^. Such work has resulted in deliberate efforts to incorporate concerns of equity into environmental policymaking^11^. Little work has been done examining if socially disadvantaged and marginalized communities are also disproportionately burdened by particulate matter pollution in low- and middle-income countries like India^12^, although such work could inspire similar policy efforts.

In India, limited existing evidence has shown that pollution from coal fired power plants is higher among marginalized populations belonging to lower castes and among the poor^13^. Recent work has also found disparities in air pollution-related mortality from power generation plants, with poorer, coal-dependent states in eastern India bearing the brunt of PM_2.5_-mortality from electricity generation^14^. Other research has found that PM_2.5_ levels are higher in districts with a higher percentage of lower caste or Scheduled Caste (SC) residents, young children, and households in poor condition^15^. Scheduled Castes and Scheduled Tribes, and Other Backward Classes are officially designated groups of people who are among the most socioeconomically disadvantaged in India; and that the greatest increase in PM_2.5_ concentrations were in less urbanized districts with a high percentage of SCs, women, children, persons with disabilities, and households without toilets^15^.

The EJ studies described here draw on data for socioeconomic status (SES) from the Census as well as the Houselisting and Housing Census data of India^13,15^. As the Census is conducted once every 10 years, some of the variables, such as asset ownership, likely do not reflect the current distribution of wealth. In addition, the Census datasets contain limited information on SES information relevant to evaluating environmental justice concerns in India. For example, religion is typically not recorded. Finally, existing research that has utilized these datasets have been conducted at the district-level which may not be a fine-enough spatial scale to capture the substantial heterogeneity in PM_2.5_ and SES status in India. We aim to fill these gaps in the current study by evaluating how total (main analysis), anthropogenic PM_2.5_ levels, and source-specific PM_2.5_ concentrations (supplementary analyses) vary over a rich array of context-specific SES variables relating to caste, religion, income, education, household assets and wealth associated with social advantage in India, using data from the National Family and Health Survey (NFHS-4) conducted between 2015-2016.

The NFHS are nationally representative surveys measuring indicators of population, health and nutrition, with a focus on maternal and infant health. In the NFHS, women between 15 and 49 years of age from ~ 25-30 households, sampled randomly from each of 28,526 clusters (villages in rural areas and census enumeration blocks in urban areas), which were in turn randomly sampled from each district in India were interviewed in detail. Using a precision-weighted method (described in more detail in “Methods” Section) that accounts for the NFHS-4 survey design and sampling variability^16,17^, we estimated the prevalence of the following SES factors at the cluster-level from the survey responses for the time period 2010-2015: (1) Households that were in the lowest wealth quintile, (2) Households that had Below Poverty Line (BPL) ration cards, (3) Households that had electricity, (4) Households that had improved sanitation, (5) Households that used solid fuels for their energy needs, (6) Households that had access to safe drinking water, (7) Households headed by a Muslim, (8) Households headed by a college-educated individual, (9) Households headed by a woman, (10) Households headed by an individual belonging to a Scheduled Caste (SC), (11) Households headed by an individual belonging to a Scheduled Tribe (ST), (12) Households headed by an individual belonging to an Other Backward Class (OBC), (13) Mothers married young (< 18 years of age), and (14) Underweight mothers (BMI < 18.5 kg/m^2^), an indicator of food-access (Figs. S1–4). We also used population density available for each cluster in our analysis (Fig. S5).

Total-PM_2.5_ concentrations, averaged over the years 2010-2015 were obtained from a well-validated satellite-derived dataset^18^. The PM_2.5_ sources considered in this analysis are Agricultural Residue Burning (ARB), Domestic Burning (DOM), Industrial (IND), International (INT), OTH (other), POW (power), road dust (RD), and Transport (TRA). We derived anthropogenic PM_2.5_ values by deducting soil dust from natural sources from total PM_2.5_ levels. Source- and species- specific PM_2.5_ concentrations were obtained from the output of the Community Multiscale Air Quality (CMAQ) model, described elsewhere for the year 2016, alone^19^. We chose to consider anthropogenic concentrations, in addition to total levels, because policymakers have control over the former exposure.

We first visually examined relationships between the PM levels and SES variables considered by plotting mean PM concentrations for clusters categorized into deciles based on the prevalence of different SES variables. We used multilevel models to quantify the geographic variation of total, anthropogenic and source-specific PM_2.5_ across different spatial scales. We evaluated associations between each exposure of interest and the SES factors described in unadjusted and fully-adjusted multilevel models. We also evaluated how disparities in exposure to PM_2.5_ from power generation (POW) varied *relative* to the benefits consumers receive. We used average nighttime luminosity as a proxy for energy consumption from power generation. Finally, we evaluated associations between the change in PM_2.5_ levels between 2010 and 2015 with changes in different SES factors (for more details refer to “Methods” section).

## Results

Descriptive statistics of the SES parameters and PM_2.5_ concentrations for the 28,072 clusters with non-missing SES and total PM_2.5_ levels are displayed in Table S1 in Supplementary Information*.* PM_2.5_ levels are high in India, with mean concentrations of 53.4 μg/m^3^ (median: 47 μg/m^3^; range: 3.5–131.7 μg/m^3^). Descriptive statistics for 27,534 clusters that have non-missing SES, anthropogenic, and source-specific PM_2.5_ levels are displayed in Table S2. Pair-wise Pearson correlation coefficients between the different parameters considered are displayed in Fig. S12.

### Evaluating disparities in PM concentrations along different EJ dimensions

One-way ANOVA tests revealed that total-PM_2.5_ concentrations varied significantly over all clusters classified into deciles based on the prevalence of all SES parameters, considered. When repeating this analysis for urban clusters, alone, we generally observed similar results with one exception: total-PM_2.5_ levels did not vary significantly over urban clusters categorized into deciles based on the prevalence of poor residents.

Mean total-PM_2.5_ concentrations were on average higher in clusters corresponding to a high prevalence of SCs (Decile 10: 60.7 μg/m^3^, Decile 1: 21.2 μg/m^3^), OBCs (Decile 10: 54.1 μg/m^3^, Decile 1: 28.0 μg/m^3^), Muslims (Decile 10: 56.1 μg/m^3^, Decile 1: 38.3 μg/m^3^), poor households (Decile 10: 59.3 μg/m^3^, Decile 1: 58.8 μg/m^3^), households with no formal education (Decile 10: 56.5 μg/m^3^, Decile 1: 44.9 μg/m^3^), underweight mothers (Decile 10: 55.7 μg/m^3^, Decile 1: 35.4 μg/m^3^) and mothers who were married young (Decile 10: 59.1 μg/m^3^, Decile 1: 40.7 μg/m^3^). Mean total-PM_2.5_ levels were lower in clusters with a high percentage of STs (Decile 10: 31.6 μg/m^3^, Decile 1: 72.3 μg/m^3^), and electrified households (Decile 10: 58.1 μg/m^3^, Decile 1: 72.0 μg/m^3^). STs tend to live in remote rural areas^20^, which explains the trend observed in PM_2.5_ levels.

**Figure 1:**
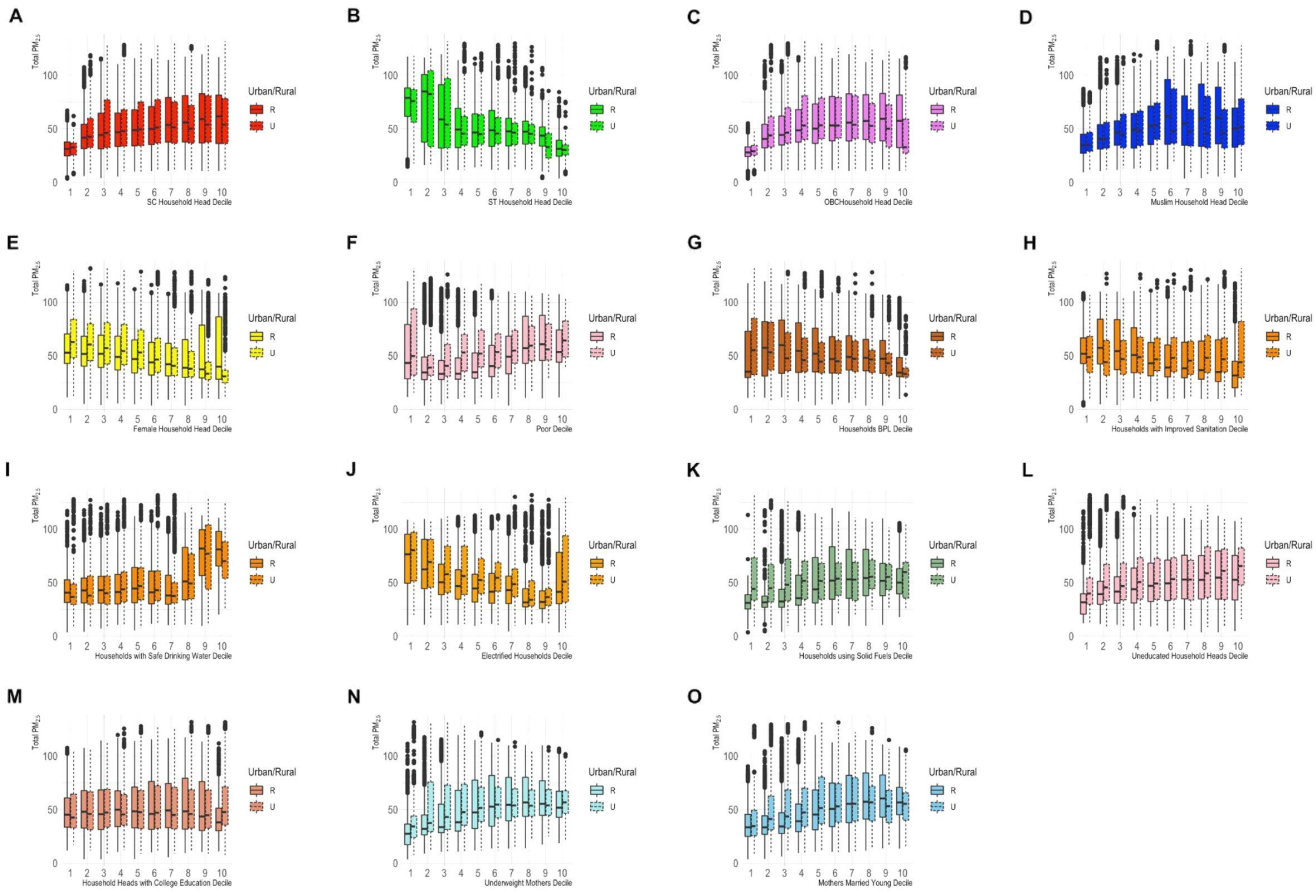
Total-PM_2.5_ concentrations by decile of the different SES prevalence parameters considered, disaggregated by urban/rural clusters. Total-PM_2.5_ concentrations corresponding to the first and tenth decile are highlighted. The boxes correspond to the first and third quartiles of the distribution of Total-PM_2.5_ concentrations corresponding to each group.

Contrary to expectations, total-PM_2.5_ levels were higher in clusters with a higher prevalence of households with safe drinking water (Decile 10: 76.7 μg/m^3^, Decile 1: 43.4 μg/m^3^), in clusters with a high prevalence of college-educated household heads (Decile 1: 49.3 μg/m^3^, Decile 10: 55.2 μg/m^3^), and in clusters with a lower prevalence of households headed by women (Decile 10: 51.2 μg/m^3^, Decile 1: 59.4 μg/m^3^). Total-PM_2.5_ concentrations were also lower in clusters with a higher prevalence of households living below the poverty line with ration cards (Decile 10: 39.7 μg/m^3^, Decile 1: 57.6 μg/m^3^). There is a negative correlation between the prevalence of poverty and households with safe drinking water (− 0.16), and almost no correlation (0.00) between the prevalence of households headed by women and poverty (Fig. S12). Although the prevalence of poverty and the prevalence of households living BPL with ration cards were correlated (0.37), not all households BPL can avail of a ration card due to limitations imposed by state quotas. The quotas rely on data from National Sample Survey (NSS) Household Consumption Survey for 2011–2012 which are outdated. Research has shown that it is often the most underprivileged who cannot access ration cards even though they are BPL^21^. Thus, the prevalence of households BPL, likely, does not capture the poorest of the poor in India. When we repeated this analysis, disaggregated by urban/rural designation, we observed similar trends among urban and rural clusters (Fig. 1).

We observed similar relationships between anthropogenic-PM_2.5_ levels and SES parameters (Fig. S13). One-way ANOVA tests revealed that anthropogenic-PM_2.5_ concentrations varied significantly over all clusters classified into deciles based on the prevalence of all SES parameters except college-educated household heads. The same was true for the variation of industrial-PM_2.5_, agricultural residue burning-PM_2.5_, and other-PM_2.5_ levels. Transport-PM_2.5_ varied significantly over all clusters categorized by the prevalence of all SES variables. The same was true for power-PM_2.5_ concentrations except over clusters classified into deciles on the basis of the prevalence of Muslim household and heads, and households with safe drinking water; And for domestic burning-PM_2.5_ levels except over clusters classified into deciles on the basis of the prevalence of households BPL; And for road dust-PM_2.5_ except for clusters classified into deciles on the basis of the prevalence households with a female head; And for international-PM_2.5_ except over clusters classified into deciles on the basis of the prevalence of households with underweight mothers. A full description of the variation of source-specific PM_2.5_ across clusters classified on the basis of the prevalence of different SES parameters can be found in section S2 in the *SI*.

When evaluating the distribution of SES parameters for different concentrations of total-PM_2.5_, we observed that at higher total-PM_2.5_ levels, there was a greater prevalence of SCs (prevalence at Decile 1: 0.10, Decile 10: 0.23), OBCs (Decile 1: 0.30, Decile 10: 0.48), Muslims (Decile 1: 0.10, Decile 10: 0.13), poor households (Decile 1: 0.10, Decile 10: 0.25), households that used solid fuels (Decile 1: 0.53, Decile 10: 0.59), households that were headed by someone with no formal education (Decile 1: 0.25, Decile 10: 0.30), households with a college educated head (Decile 1: 0.08, Decile 10: 0.11), underweight/thin mothers (Decile 1: 0.12, Decile 10: 0.21), and mothers who had married young (Decile 1: 0.30, Decile 10: 0.45).

The prevalence of the following SES parameters were lower for clusters experiencing high levels of total-PM_2.5_: household headed by an ST (Decile 1: 0.43, Decile 10: 0.01), households below the poverty line with ration cards (Decile 1: 0.39, Decile 10: 0.25), households with improved sanitation (Decile 1: 0.77, Decile 10: 0.51), and electrified households (Decile 1: 0.94, Decile 10: 0.79) (Fig. S22).

### Evaluating variation in PM_2.5_ across multiple geographic scales

When we evaluated the partitioning of variation in total PM_2.5_ concentrations by the different geographic scales using multilevel models that only controlled for the logarithm of population density and urban/rural, we found that most of the variation (> 80%) was observed at the state-level, ~ 15% of the variation in total PM_2.5_ concentrations was observed at the district level, while the remaining was at the cluster-level (Table S2). Further adjusting for SES variables only explained a small proportion of variance (~ 1%) in PM_2.5_ concentrations at each spatial scale (Table S3). We found similar results when evaluating the partitioning of variation for anthropogenic PM_2.5_ levels (Table S4), and source-specific PM_2.5_ values (Table S5). The large variation of PM_2.5_ concentrations at the state-spatial scale indicates that tackling large regional sources should be a priority in tackling pollution in India. These results could also suggest that more detailed ground-based PM_2.5_ measurements and emission inventories are needed to capture fine-scale PM variations in India.

### Evaluating associations between PM_2.5_ levels and different EJ dimensions

We used unadjusted and fully-adjusted multilevel models to evaluate associations between total PM exposures considered in this study and the various SES parameters. In order to compare associations across the different SES variables, we standardized each variable in the model using z-scores. More information can be found in “Methods” Section.

From the fully-adjusted multilevel models, we found that an increasing prevalence of SC, OBC households were associated with small but significant increases in total-PM_2.5_ concentrations. An increasing prevalence of poor, electrified, ST, Muslim households, households BPL with ration cards, underweight or thin mothers were associated with decreasing levels of total-PM_2.5_. Specifically, we observed that a 1 standard deviation (SD) increase in the prevalence of households with an OBC head was associated with the largest increase in total-PM_2.5_ concentrations of 0.199 μg/m^3^ (95% CI 0.116 μg/m^3^, 0.283 μg/m^3^). The next largest positive association was observed with the SES parameter: the prevalence of SC households: 0.127 μg/m^3^ (95% CI 0.062 μg/m^3^, 0.129 μg/m^3^), followed by the prevalence of mothers married young: 0.106 μg/m^3^ (95% CI − 0.003 μg/m^3^, 0.215 μg/m^3^).

The largest negative association was observed with the SES variable: the prevalence of ST households: − 0.383 μg/m^3^ (95% CI − 0.497 μg/m^3^, − 0.269 μg/m^3^) and the prevalence of households living in poverty: − 0.260 μg/m^3^ (95% CI − 0.376 μg/m^3^, − 0.144 μg/m^3^). The latter association diverges from our initial hypothesis that lower total-PM_2.5_ concentrations would be present in richer clusters (Table 1).

**Table 1:** Associations between the different SES parameters and total-PM_2.5_ in models only adjusted for urban/rural and the logarithm of population density, as well in fully-adjusted models, mutually adjusted for other SES parameters.

	Total PM_2.5_			
	All		Urban	Rural
	Unadjusted	Fully-adjusted	Fully-adjusted	Fully-adjusted
SC household heads	0.110* (0.061, 0.160)	0.127* (0.062, 0.192)	− 0.030 (− 0.125, 0.065)	0.198* (0.114, 0.282)
ST household heads	− 0.619* (− 0.706, − 0.531)	− 0.383* (− 0.497, − 0.269)	− 0.032 (− 0.231, 0.166)	− 0.371* (− 0.515, − 0.228)
OBC household heads	0.249* (0.187, 0.311)	0.199* (0.116, 0.283)	− 0.032 (− 0.160, 0.096)	0.245* (0.139, 0.350)
Muslim household heads	− 0.075* (− 0.129, − 0.021)	− 0.083* (− 0.141, − 0.025)	− 0.017 (− 0.104, 0.069)	− 0.123* (− 0.199, − 0.048)
Female household heads	− 0.009 (− 0.094, 0.076)	0.007 (− 0.080, 0.095)	− 0.136* (− 0.268, − 0.003)	0.056 (− 0.053, 0.165)
Poor	− 0.320* (− 0.396, − 0.245)	− 0.260* (− 0.376, − 0.144)	0.081 (− 0.056, 0.219)	− 0.298* (− 0.441, − 0.156)
Households BPL with ration cards	− 0178* (− 0.261, − 0.095)	− 0.094* (− 0.184, − 0.005)	0.001 (− 0.140, 0.141)	− 0.096 (− 0.201, 0.010)
Households with improved sanitation	0.112* (0.037, 0.188)	− 0.086 (− 0.187, 0.016)	0.123* (0.010, 0.236)	− 0.137* (− 0.256, − 0.018)
Households with safe drinking water	0.089* (0.033, 0.144)	0.034 (− 0.022, 0.091)	− 0.127* (− 0.216, − 0.039)	0.111* (0.041, 0.181)
Electrified households	0.067* (0.003, 0.132)	− 0.095* (− 0.175, − 0.015)	0.025 (− 0.088, 0.138)	− 0.119* (− 0.216, − 0.022)
Households using solid fuels	− 0.098* (− 0.181, − 0.015)	0.034 (− 0.073, 0.140)	− 0.055 (− 0.185, 0.074)	− 0.029 (− 0.126, 0.067)
Uneducated household heads	− 0.172* (− 0.238, − 0.106)	− 0.047 (− 0.129, 0.035)	0.044 (− 0.077, 0.165)	− 0.088 (− 0.184, 0.008)
Household heads with college education	0.006 (− 0.046, 0.058)	− 0.005 (− 0.066, 0.056)	− 0.007 (− 0.100, 0.086)	− 0.055 (− 0.121, 0.010)
Mothers married young	0.080 (− 0.023, 0.183)	0.106 (− 0.003, 0.215)	0.130 (− 0.035, 0.295)	0.094 (− 0.037, 0.226)
Underweight mothers	− 0.146* (− 0.230, − 0.062)	− 0.096* (− 0.184, − 0.007)	0.030 (− 0.102, 0.162)	− 0.116* (− 0.220, − 0.011)

We also display associations from fully-adjusted models disaggregated by urban/rural.

*(*p* < 0.05).

When evaluating these associations, disaggregated by urban/rural, we observed substantial differences. For instance, we observed positive associations between total-PM_2.5_ and the prevalence of SC: 0.198 μg/m^3^ (95% CI 0.114 μg/m^3^, 0.282 μg/m^3^) and OBC household heads: 0.245 μg/m^3^ (95% CI 0.139 μg/m^3^, 0.350 μg/m^3^) in rural clusters, respectively, but not urban clusters: − 0.030 μg/m^3^ (95% CI − 0.125 μg/m^3^, 0.065 μg/m^3^) and − 0.032 μg/m^3^ (95% CI − 0.231 μg/m^3^, 0.166 μg/m^3^), respectively. The negative association between total-PM_2.5_ levels and the prevalence of ST household heads was significant in rural clusters: − 0.371 μg/m^3^ (95% CI − 0.515 μg/m^3^, − 0.228 μg/m^3^), but not urban clusters: − 0.032 μg/m^3^ (95% CI − 0.231 μg/m^3^, 0.166 μg/m^3^), although the association in urban clusters demonstrated the same general trend observed in rural clusters.

We observed significant negative associations between total-PM_2.5_ levels in urban clusters with a higher prevalence of female-headed households: − 0.136 μg/m^3^ (95% CI − 0.268 μg/m^3^, − 0.003 μg/m^3^), but not in rural clusters: 0.056 μg/m^3^ (95% CI − 0.053 μg/m^3^, 0.165 μg/m^3^). We observed significant negative associations between total-PM_2.5_ levels in rural clusters with a higher prevalence of households living in poverty: − 0.298 (95% CI − 0.441, − 0.156), but not in urban areas: 0.081 (95% CI − 0.056, 0.219).

We noted significant associations between total-PM_2.5_ concentrations and the prevalence of households with improved sanitation : 0.123 μg/m^3^ (95% CI 0.010 μg/m^3^, 0.236 μg/m^3^) and households with safe drinking water: − 0.127 μg/m^3^ (95% CI − 0.216 μg/m^3^, − 0.039 μg/m^3^) in urban clusters, compared with − 0.137 μg/m^3^ (95% CI − 0.256 μg/m^3^, − 0.018 μg/m^3^) and 0.111 μg/m^3^ (95% CI 0.041 μg/m^3^, 0.181 μg/m^3^), respectively, in rural locations (Table 1). Our results suggest that different dimensions of inequality operate differently in urban and rural clusters.

When evaluating associations between the various SES parameters considered with anthropogenic-PM_2.5_ levels, we observed similar trends in associations to those estimated with total-PM_2.5_ levels (Table S6), with some differences. Namely, the general trend of associations between anthropogenic-PM_2.5_ concentrations and the prevalence of households with a Muslim head was positive: 0.010 μg/m^3^ (95% CI − 0.037 μg/m^3^, 0.056 μg/m^3^), whereas it was negative when considering total-PM_2.5_ levels. The same trend was observed when evaluating associations with the prevalence of households with improved sanitation: Associations with anthropogenic-PM_2.5_ were − 0.029 μg/m^3^ (95% CI − 0.089 μg/m^3^, 0.032 μg/m^3^), while those with total-PM_2.5_ were 0.112 μg/m^3^ (95% CI 0.037 μg/m^3^, 0.144 μg/m^3^).

We also reported associations with source-specific PM_2.5_ exposures using unadjusted (Table S7), fully-adjusted models (Table S8), and disaggregated by urban/rural designation (Table S9). We observed substantial differences in the magnitude and direction of associations observed with the different SES parameters considered. For example, PM_2.5_ from domestic burning is significantly lower in clusters with a higher prevalence of household heads with no formal education; However, we observed the opposite trend for PM_2.5_ from agricultural residue burning (for more details refer to section S4). The differences in associations are a result of the different distribution of sources (agricultural land, for example), as well as SES parameters (access to reliable electricity, among others). The latter is an environmental justice concern as research has shown that villages inhabited solely by SCs are significantly less likely to be electrified^22^ than other villages; while the former is not. Another possible reason for the difference in results is that so far, we assume a linear relationship between PM exposures and the different SES parameters considered. In future sections, we relax this assumption.

### Evaluating associations between PM levels and different EJ dimensions after accounting for potential non-linearities

**Figure 2:**
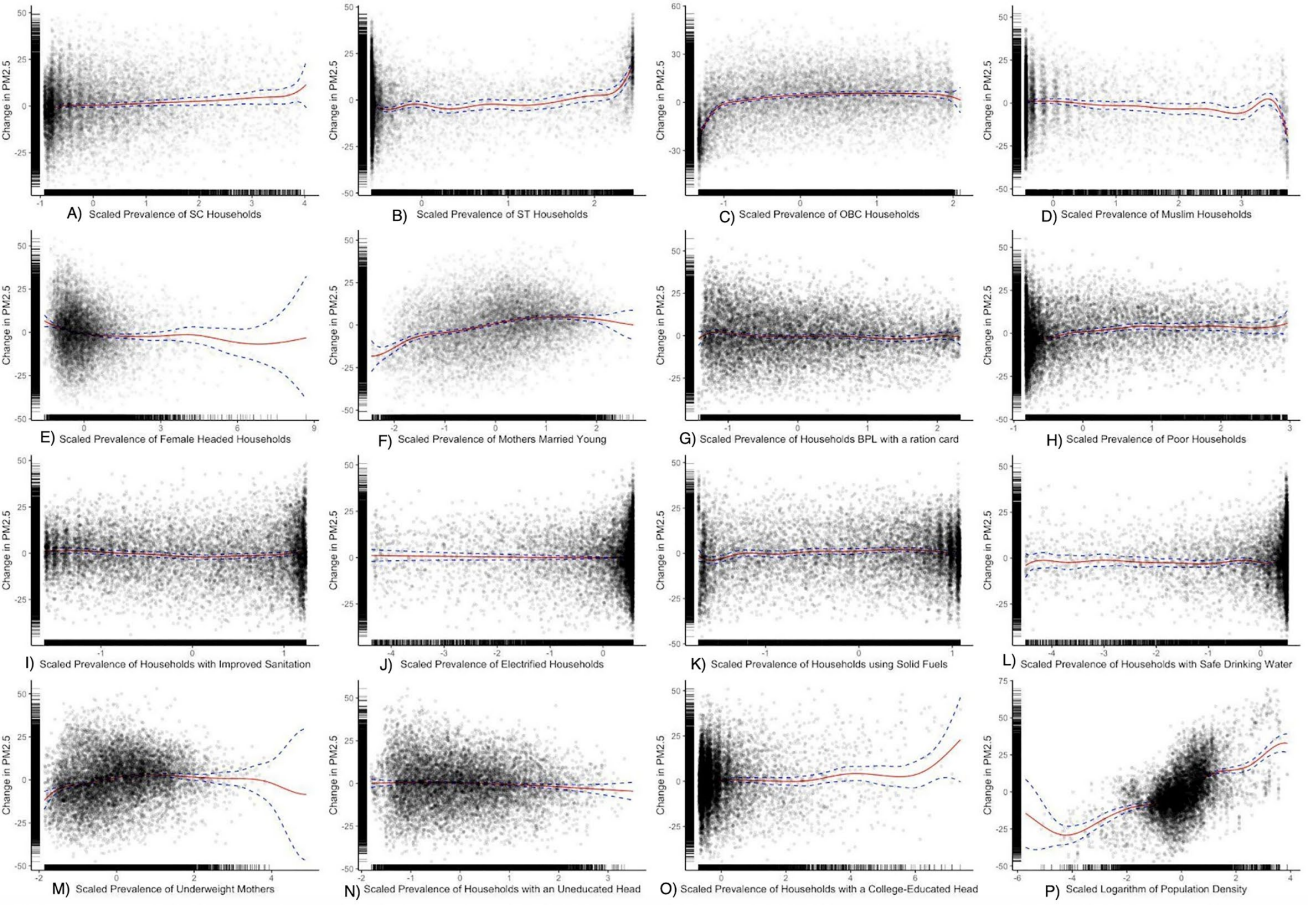
Partial response plot (red line) and 95% CI (between the blue dashed lines) for the association between total-PM_2.5_ and the prevalence of (**A**) SC households, (**B**) ST households, (**C**) OBC households, (**D**) Muslim households, (**E**) Households with a female head, (**F**) Mothers married young < 18 y of age), (**G**) Households BPL, (**H**) Poor Households, (**I**) Households with improved sanitation, (**J**) Electrified households, (**K**) Households using solid fuels, (**L**) Households with safe drinking water, (**M**) Underweight mothers, (**N**) Household head without formal education, (**O**) Household head with college-educated head, and (**P**) Population density in fully-adjusted models. We also display partial residual points and rug plots to provide readers with an understanding of the distribution of variables considered.

After accounting for potential non-linearities between the different SES parameters and total-PM_2.5_, there are several important nuances regarding associations between PM_2.5_ and the SES under consideration (Fig. 2). For example, when evaluating non-linear associations between total-PM_2.5_ and religion, caste, and gender-related SES, we find that although total-PM_2.5_ decreases on average with a unit increase in prevalence of ST households, total-PM_2.5_ concentrations *increase* in clusters with the highest prevalence of ST households. We observe a similar result in associations between totla-PM_2.5_ concentrations and clusters with a high prevalence of Muslim households (Fig. 2). Note that clusters with the highest prevalence of Muslim households have the lowest total-PM_2.5_ levels.

When evaluating associations between total-PM_2.5_ and the prevalence of poverty, we note that total-PM_2.5_ levels increase after initially decreasing for clusters corresponding to the highest prevalence of poverty (Fig. 2). We observed that the associations between total-PM_2.5_ and various household characteristics such as access to drinking water, access to improved sanitation, and access to electricity were fairly constant across different levels of these SES parameters. Total-PM_2.5_ concentrations first increased with the increasing prevalence of underweight mothers and then decreased (Fig. 2). Finally, we observed increases in total-PM_2.5_ in clusters with a higher prevalence of college-educated household heads (Fig. 2). These plots suggest that it is important to take into consideration non-linearity when evaluating associations between total-PM_2.5_ and SES in India. We note similar nonlinearities when evaluating associations between anthropogenic-PM_2.5_ levels and SES (Fig. S23), and between source-specific PM_2.5_ levels and SES (Figs. S24–S31) in supplementary analyses.

### Evaluating associations between the ratio of power-PM levels and nighttime luminosity and different EJ dimensions

A higher prevalence of ST households in a cluster was significantly associated with a decrease in total-PM_2.5_ concentrations (Table 1); but was not significantly associated with changes in PM_2.5_ from power-generation (Table S8). However, we noticed significant disparities in exposure to PM_2.5_ from power generation *relative* to the benefits consumers receive (nighttime luminosity as a proxy for electricity use).

Specifically, the association between PM_2.5_ from power generation (burden) relative to nighttime luminosity (benefit) and the prevalence of ST households was significant: 15.187 (95% CI 3.829, 26.539) (Table 2). We observe similar results for clusters with a high prevalence of households in poverty: 15.970 (95% CI 3.459, 28.629). Unsurprisingly, we find that exposure relative to the benefits of PM_2.5_ from power generation is low in clusters with a high prevalence of electrified households, and safe drinking water (proxies of power consumption) (Table 2). Our analysis thus explores environmental justice concerns beyond looking at the distributional impacts of PM_2.5_, to the distributional impacts *relative* to the benefit from a key source of PM_2.5_.

**Table 2:** Associations between the different SES parameters and Power generation- PM_2.5_/Nighttime Luminosity in fully-adjusted models, mutually adjusted for other SES parameters.

	Power-PM_2.5_/nighttime luminosity		
	All	Urban	Rural
	Fully-adjusted	Fully-adjusted	Fully-adjusted
SC household heads	1.172 (− 5.952, 8.322)	0.450 (− 0.455, 1.350)	6.872 (− 3.544, 17.348)
ST household heads	15.187* (3.829, 26.539)	0.268 (− 1.140, 1.658)	15.030 (− 1.168, 31.281)
OBC household heads	− 4.086 (− 12.953, 4.848)	1.046 (− 0.098, 2.184)	− 0.673 (− 13.395, 12.173)
Muslim household heads	2.020 (− 4.257, 8.279)	0.793 (− 0.023, 1.619)	3.599 (− 5.608, 12.806)
Female household heads	5.660 (− 2.677, 13.927)	1.300* (0.372, 2.251)	16.877* (5.150, 28.523)
Poor	15.970* (3.459, 28.629)	0.131 (− 1.161, 1.423)	11.271 (− 6.141, 28.929)
Households BPL with ration cards	5.986 (− 3.414, 15.402)	− 0.379 (− 1.530, 0.783)	1.695 (− 10.861, 14.242)
Households with improved sanitation	15.149* (4.192, 26.067)	0.049 (− 0.995, 1.096)	7.673 (− 6.679, 22.013)
Households with safe drinking water	− 10.934* (− 17.147, − 4.810)	− 0.593 (− 1.431, 0.202)	− 10.409* (− 19.149, − 1.772)
Electrified households	− 35.462* (− 44.226, − 26.649)	− 0.723 (− 1.778, 0.322)	− 43.548* (− 55.770, − 31.290)
Households using solid fuels	5.500 (− 5.989, 16.950)	0.826 (− 0.302, 2.009)	7.469 (− 4.100, 19.034)
Uneducated household heads	6.741 (− 1.969, 15.405)	− 1.443* (− 2.568, − 0.367)	2.545 (− 8.931, 13.918)
Household heads with college education	3.885 (− 2.864, 10.673)	− 0.132 (− 1.017, 0.745)	1.379 (− 6.930, 9.761)
Mothers married young	− 24.767* (− 34.838, − 14.605)	0.645 (− 0.508, 1.770)	− 27.978* (− 41.578, − 14.288)
Underweight mothers	− 18.777* (− 27.696, − 9.840)	− 0.759 (− 1.840, 0.294)	− 17.476* (− 113.368, − 86.483)

We also display associations from fully-adjusted models disaggregated by urban/rural.

*(*p* < 0.05).

### Evaluating associations between the difference and percentage difference in PM_2.5_ concentrations in each cluster between 2015 and 2020 and different EJ dimensions

Overall, the mean difference in PM_2.5_ concentrations between 2010 and 2015 was 0.66 (min: − 24.4 μg/m^3^, max: 16.5 μg/m^3^, median: 0.8 μg/m^3^, standard deviation: 5.0 μg/m^3^). The mean percentage difference was 2.6% (min: − 22.3%, max: 24.9%, median: 1.9%, standard deviation: 9.5%).

We observed that there was a significant increase in PM_2.5_ increase levels 2015 relative to 2010 of 0.024 μg/m^3^ (95% CI 0.013 μg/m^3^, 0.047 μg/m^3^) for every standard deviation increase in the prevalence of Muslim households (Table 3). We observed the same general trend when evaluating associations between the percentage difference in PM_2.5_ levels between 2010 and 2015, relative to 2010 levels, instead of the absolute difference in PM_2.5_ levels in urban but not rural areas. In rural areas, there were significant decreases in PM_2.5_ levels in clusters with a higher prevalence of SC: − 0.040 μg/m^3^ (95% CI − 0.072 μg/m^3^, − 0.009 μg/m^3^), ST: − 0.054 μg/m^3^ (95% CI − 0.108 μg/m^3^, 0.000 μg/m^3^), OBC: − 0.042 μg/m^3^ (95% CI − 0.082 μg/m^3^, − 0.003 μg/m^3^), and mothers married < 18 years of age: − 0.062 μg/m^3^ (95% CI − 0.112 μg/m^3^, − 0.013 μg/m^3^); while we observed an increase in clusters with a higher prevalence of underweight mothers: 0.048 μg/m^3^ (95% CI 0.009 μg/m^3^, 0.088 μg/m^3^). We observed similar results in rural areas when evaluating associations between the percentage difference in PM_2.5_ levels, instead (Table 3). Our results that in recent years, overall, religion is becoming an increasingly important lens in India to evaluate EJ patterns.

**Table 3:** Associations between the different SES parameters and difference and percent difference in PM_2.5_ between 2015 and 2020 in fully-adjusted models, mutually adjusted for other SES parameters.

	Difference in PM_2.5_ levels (μg/m^3^) between 2015 and 2010			Percent difference in PM_2.5_ levels between 2015 and 2010		
	All	Urban	Rural	All	Urban	Rural
SC household heads	− 0.014 (− 0.039, 0.012)	− 0.005 (− 0.048, 0.038)	− 0.040* (− 0.072, − 0.009)	− 0.036 (− 0.087, 0.015)	− 0.011 (− 0.087, 0.066)	− 0.111* (− 0.176, − 0.046)
ST household heads	− 0.021 (− 0.066, 0.023)	0.027 (− 0.063, 0.116)	− 0.054* (− 0.108, 0.000)	− 0.018 (− 0.107, 0.072)	0.091 (− 0.069, 0.251)	− 0.110 (− 0.221, 0.001)
OBC household heads	− 0.018 (− 0.051, 0.015)	− 0.004 (− 0.062, 0.054)	− 0.042* (− 0.082, − 0.003)	− 0.056 (− 0.122, 0.009)	− 0.035 (− 0.139, 0.069)	− 0.137* (− 0.219, − 0.055)
Muslim household heads	0.024* (0.013, 0.047)	0.014 (− 0.025, 0.054)	0.028 (− 0.001, 0.056)	0.011 (− 0.035, 0.056)	0.006 (− 0.064, 0.077)	− 0.010 (− 0.069, 0.048)
Female household heads	0.004 (− 0.030, 0.039)	− 0.031 (− 0.091, 0.029)	0.017 (− 0.025, 0.058)	0.023 (− 0.046, 0.091)	− 0.088 (− 0.194, 0.019)	0.064 (− 0.021, 0.149)
Poor	− 0.003 (− 0.049, 0.043)	− 0.093* (− 0.155, − 0.030)	0.012 (− 0.041, 0.066)	0.135* (0.044, 0.226)	− 0.146* (− 0.257, − 0.034)	0.140* (0.029, 0.250)
Households BPL with ration cards	− 0.051* (− 0.086, − 0.016)	− 0.021 (− 0.084, 0.043)	− 0.029 (− 0.069, 0.010)	− 0.071* (− 0.142, − 0.001)	− 0.025 (− 0.138, 0.089)	− 0.059 (− 0.141, 0.023)
Households with improved sanitation	0.016 (− 0.024, 0.056)	0.050 (− 0.001, 0.101)	0.001 (− 0.044, 0.046)	− 0.031 (− 0.111, 0.049)	0.056 (− 0.035, 0.148)	− 0.005 (− 0.097, 0.088)
Households with Safe drinking water	− 0.010 (− 0.032, 0.013)	− 0.017 (− 0.057, 0.023)	− 0.018 (− 0.044, 0.008)	− 0.036 (− 0.080, 0.009)	− 0.050 (− 0.122, 0.021)	− 0.042 (− 0.096, 0.012)
Electrified households	− 0.005 (− 0.037, 0.026)	− 0.042 (− 0.093, 0.010)	− 0.001 (− 0.038, 0.036)	0.043 (− 0.019, 0.106)	− 0.037 (− 0.129, 0.054)	0.037 (− 0.039, 0.113)
Households using solid fuels	0.022 (− 0.020, 0.064)	0.145* (0.087, 0.204)	− 0.019 (− 0.056, 0.017)	− 0.131* (− 0.215, − 0.048)	0.236* (0.132, 0.341)	− 0.150* (− 0.225, − 0.075)
Uneducated household heads	0.018 (− 0.015, 0.050)	0.033 (− 0.022, 0.088)	0.012 (− 0.025, 0.048)	0.026 (− 0.039, 0.091)	0.027 (− 0.071, 0.125)	0.045 (− 0.030, 0.120)
Household heads with college education	0.023 (− 0.001, 0.047)	0.058 (− 0.022, 0.088)	0.021 (− 0.004, 0.046)	0.039 (− 0.009, 0.086)	0.095* (0.020, 0.170)	0.044 (− 0.007, 0.094)
Mothers married Young	− 0.040 (− 0.082, 0.003)	− 0.041 (− 0.116, 0.033)	− 0.062* (− 0.112, − 0.013)	− 0.096* (− 0.181, − 0.011)	− 0.057 (− 0.190, 0.076)	− 0.179* (− 0.281, − 0.078)
underweight mothers	0.023 (− 0.011, 0.058)	− 0.025 (− 0.085, 0.034)	0.048* (0.009, 0.088)	0.068 (− 0.001, 0.137)	− 0.091 (− 0.130, 0.083)	0.096* (0.015, 0.177)

We also display associations from fully-adjusted models disaggregated by urban/rural.

*(*p* < 0.05).

We also noted a significant decrease in levels of − 0.051 μg/m^3^ (95% CI − 0.086 μg/m^3^, − 0.016 μg/m^3^) for every standard deviation increase in the prevalence of households BPL. We observed similar results when using the percentage difference in PM_2.5_ levels: − 0.071% (95% CI − 0.142%, − 0.001%) (Table 3). However, we noted a significant increase in the percentage difference in PM_2.5_ concentrations for every increase in the prevalence of poor households, overall: 0.135% (95% CI 0.044%, 0.226%), and in rural areas: 0.140% (95% CI 0.029%, 0.250%). We observed the opposite results in urban areas: − 0.146% (95% CI − 0.257%, − 0.034%) (Table 3). Our results suggest that villages in rural areas with a high prevalence of poorer residents, without access to services such as ration cards are vulnerable to increases in PM_2.5_ concentrations, relative to base levels.

Finally, we observed significant decreases in the percentage difference in PM_2.5_ levels for every increase in the prevalence of solid fuels, overall: − 0.131% (95% CI − 0.215%, − 0.048%) and in rural areas: − 0.150% (95% CI − 0.225%, − 0.075%), and opposite results in urban clusters: 0.236% (95% CI 0.132%, 0.341%) (Table 3).

## Discussion

We curated a dataset of total, anthropogenic, and source-specific PM_2.5_ levels and SES variables associated with social advantage in India for a nationally representative set of clusters in India, which are villages in rural areas and census enumeration blocks in urban areas for the year 2015. Evaluating the variation in total-PM_2.5_ across multiple geographic scales, revealed that most variation occurred at the state-level, indicating that tackling large regional sources should be a priority in tackling pollution in India. This result could also suggest that more detailed ground-based PM_2.5_ measurements and emission inventories are needed to capture fine-scale PM variations in India.

In many regions of the world there is a growing understanding in EJ research that although identifying risk factors such as race, education and income is important, it is just as important to identify structural factors that result in such disparities^23,24^. Our research suggests that SES factors such as caste, religion, poverty, education, and access to various household amenities are important risk factors for PM_2.5_ exposures. Specifically, we observed that total-PM_2.5_ levels were significantly higher in clusters with a higher prevalence of SC, OBC households, and underweight and lower in clusters with a high prevalence of Muslim, ST, poor, and electrified households. However, different directions in associations were observed when disaggregating our analysis by urban/rural designation. For example, the general trend of associations between the prevalence of poor households and total-PM_2.5_ was positive in urban areas, but negative in rural locations. When considering other PM_2.5_ exposures, we also noted differences in the direction and magnitude of associations with different SES factors. Our results suggest that different dimensions of inequality operate differently in urban and rural areas, and for different sources. Future theoretical frameworks developed to conceptualize EJ in India, need to take these empirical differences into consideration.

Our relaxation of the assumption of linearity between the PM_2.5_ exposures considered and the different SES parameters can also potentially add nuance to the conceptualization of EJ in India. Specifically, we observed that total-PM_2.5_ levels were significantly lower, overall, in clusters with a higher prevalence of poor and Muslim households. However, when we accounted for potential non-linearities in the relationship between PM_2.5_ and the SES parameters considered, we observed that PM_2.5_ levels were highest in clusters with the highest prevalence of poverty and among the highest prevalence of Muslim households. Our analyses showed that clusters with a high prevalence of Muslim residents were observing significant increases in PM_2.5_ concentrations, suggesting that religion is becoming an important axis of inequality in India.

Although, most of this work conceptualized EJ in terms of evaluating disparities in exposure to PM_2.5_, we also considered a different definition of EJ; i.e. evaluating disparities in PM_2.5_ exposure from a key-source: power generation *relative* to the benefits received (using nighttime luminosity as a proxy). We observed that ST and poor households were exposed to significantly higher exposures from power-generation relative to the benefits they received. These results point to the urgency of expanding the theoretical discourse on EJ in India.

To summarize, this research presents a comprehensive overview of disparities in exposure to air pollution along several dimensions of environmental justice in India. Our approach has some limitations. Specifically, the PM_2.5_ exposures considered have several uncertainties. For example, previous work has shown that exposure estimates derived from satellite data diverge from each other, especially in rural areas where ground-based monitors are sparse^4^. In addition, we assigned ambient exposures to all individuals based on their cluster of residence. Due to the lack of data, we did not account for differences in housing characteristics, occupational exposures, activity patterns that could influence exposure to ambient PM_2.5_ concentrations.

## Data and methods

### Socioeconomic status (SES) and demographics

We drew data from the fourth round of National Family Health Survey (NFHS-4) of India (equivalent to Demographic and Health Survey) conducted between Jan 2015 and Nov 2016^25^. NFHS are nationally representative household sample surveys measuring indicators of population, health and nutrition, with special emphasis on maternal and child health.

The NFHS-4 has a two-stage design, in which a number of clusters (villages in rural areas and census enumeration blocks in urban areas) are first selected from each of the 640 districts that existed at the time of the 2011 Census of India. Each of the 28,526 clusters was categorized as urban or rural. A household listing operation was then carried out by visiting each of the selected clusters and listing all residential households. Clusters with more than 300 households were divided into segments of 100–150 households. The resulting list of households served as a sampling frame for selection of households in the second stage. A fixed number of 22 households were selected from each cluster based on equal probability systematic sampling. Women aged 15–49 years were selected from these households for in-depth surveys. NFHS uses extensive interviewer training, standardized measurement methods, and an identical questionnaire to ensure standardization and comparability across diverse sites and times.

The GPS coordinates data for the NFHS-4 clusters were obtained via a special request. These survey cluster coordinates were collected in the field using GPS receivers, usually during the survey sample listing process. In general, the GPS readings for most clusters were accurate to less than 15 m. To ensure that respondent confidentiality was maintained, the GPS latitude/longitude positions were displaced for all clusters. The displacement was randomly carried out so that rural clusters contained a minimum of 0 and a maximum of 5 km of positional error. For 1% of the rural clusters, the displacement occurred up to 10 km. The displacement was restricted so that the points stayed within the second administrative level of the district.

We chose context-specific SES covariates to evaluate the EJ implications of pollution in India. In addition to choosing risk-factors related to income, education, household assets and wealth, which are commonly associated with social advantage, we also looked at caste- and religion-specific variables. Caste has a deep sociological history in Indian society. Lower castes now referred to as Schedule Caste (SCs), Scheduled Tribes (STs) and Other Backward Classes (OBCs) have historically been denied access to important public services. There is still strong evidence of discrimination against these groups in both the education sector and the labor market^26–28^. There is also evidence that religious minorities like Muslims have been marginalized in India^29^. We thus included these covariates as key EJ dimensions in India.

From Household Recode NFHS data, we extracted the following binary household-level covariates: (1) Poor: Household was in the lowest wealth quintile, (2) Household had a Below Poverty Line (BPL) ration card, (3) Household had electricity, (4) Household had improved sanitation, (5) Household used solid fuels for their energy needs, (6) Household had access to safe drinking water, (7) Household head was Muslim, (8) Household head had been to college, (9) Household head was uneducated, (10) Household head was female, (11) Household head belonged to a Scheduled Caste (SC), (12) Household head belonged to a Scheduled Tribe (ST), and (13) Household head belonged to an Other Backward Class (OBC).

From Individual-level NFHS-4 data from the women interviewed, we extracted the following binary level covariates: (1) Mother is uneducated, (2) Mother is literate, (3) Mother was married before 18 years of age, and (4) Mother is underweight (BMI < 18.5 kg/m^2^), an indicator of food-access.

NFHS-4 provides addition geospatial covariates for each cluster, on population density for the year 2015 (#/km^2^) within the 2 km (urban) or 10 km (rural) buffer surrounding the NFHS-4 survey cluster location. The estimate of population density is Population counts for each cluster used to produce these estimates were derived from the Gridded Population of the World, Version 4 (GPWv4). Although population density is traditionally measured as persons per square kilometer (or, square mile), a natural logarithmic transformation of this measure is used in our multivariate analysis to account for its skewed distribution, as recommended in previous EJ research. We also derived average nighttime luminosity in the form of a nightlight index (dimensionless) from the NFHS-4 geospatial data for the year 2015. (Figs. S1–S6 in *Supplementary Information* displays the spatial distribution of various SES parameters.)

We removed clusters for which we did not have information on context-specific SES covariates or population density and were left with 28,072 of a total of 28,526 clusters. Most of these clusters are in Jammu and Kashmir and Assam (Fig. S11).

### Deriving cluster-specific SES covariates

To account for the complex survey design and sampling variability, we derived cluster-specific predicted probabilities of each variable from NFHS household and individual data described in “Evaluating Disparities in PM concentrations along different EJ dimensions” Section using four-level multilevel models^16,30^. The four levels of geographic units are individuals (or households) at level-1 (i), clusters at level-2 (j), districts at level-3 (k) and states at level-4 (l). The model is presented below:

1 $$\log it(\pi_{ijkl} ) = \beta_{0} + u_{0jkl} + v_{0kl} + f_{0l}$$

$\beta_{0}$ is the constant and represents the median log odds of each covariate across all of India; $u_{0jkl}$, $v_{0kl}$, and $f_{0l}$ are the residuals at the cluster, district, and state levels, respectively. The residuals are assumed to be normally distributed with a mean 0 and a variance of $\sigma_{u0}^{2}$, $\sigma_{v0}^{2}$, and $\sigma_{f0}^{2}$. These variance terms can be interpreted as within-district between-cluster variation ($\sigma_{u0}^{2}$), within-state between-district variation ($\sigma_{v0}^{2}$), and between-state variation ($\sigma_{f0}^{2}$).

From the model described in Eq. 1, the cluster-specific logit values were converted to probabilities by taking the average over the simulations, i.e.. $exp \left( {\beta_{0} + u_{0jkl} + v_{0kl} + f_{0l} } \right) /\left( {1 + exp \left( {\beta_{0} + u_{0jkl} + v_{0kl} + f_{0l} } \right) } \right)$. For estimation, we used Monte Carlo Markov Chain (MCMC) methods with a burn-in of 5000 cycles, and monitoring of 50,000 iterations of chains. For all estimates, we used 2nd order penalized quasi-likelihood (PQL) for the estimation of starting values, but for few variables (Households with electricity, Poor households, Households with a Muslim head, Household with an ST head) the convergence failed, and we used 1st order marginalized quasi-likelihood (MQL) instead.

In this manner, cluster-specific predictions of the various covariates can be made by “shrunken” higher level residuals that consider the ratio of the between-state, between-district and between-cluster variance to the total variance, which includes the within-state, within-district and within-cluster sampling variance attributable to the sample size of districts with states, clusters within districts, and individuals within clusters. Hence, more shrinkage occurs i.e. cluster-specific means are pulled more towards district-means (and state-means) if there are fewer individuals within a cluster, and consequently higher sampling variances, and/or when the estimated variance of the clusters is small.

### Total PM_2.5_ exposure

The main exposure variable in this study was long-term ambient PM_2.5_ between the years 2010–2015. Because India lacks surface PM_2.5_ monitoring sites at the spatial resolution required for the study, and the NFHS surveys do not record PM_2.5_ concentration in each cluster, we used satellite-derived annual averaged PM_2.5_ estimates derived by Hammer et al.^18^ as the main exposure of interest as this dataset has been validated and used in several global studies^4,31^. Satellite aerosol optical depths (AODs) were combined from multiple satellite products: MISR, MODIS Dark Target, MODIS and SeaWiFS Deep Blue, and MODIS MAIAC with simulation-based results based on their relative uncertainties. These AODs were related to near-surface monthly PM_2.5_ concentrations at a 0.01° × 0.01° (~ 1 km × 1 km at the equator) resolution over the globe using the ratio of simulated AOD and PM_2.5_ from the GEOS-Chem model. We clipped these estimates to India. On an annual scale the PM_2.5_ estimates are highly consistent with globally distributed ground monitors (R^2^ = 0.90–0.92). We previously evaluated this dataset based on ground-based monitors in India and found the India-specific R^2^ was 0.55 (RMSE: 27.5)^4^. We extracted mean PM_2.5_ levels in the 2 km/5 km buffer for urban and rural household clusters respectively (Fig. S7).

Note, we opted to use a satellite-derived exposure product for this analysis, instead of model-based products that we use to map source-specific and anthropogenic PM_2.5_ concentrations discussed below, as the model-based results are available for a single year, alone. We evaluate associations between the difference in PM_2.5_ concentrations between 2010 and 2015 and SES parameters in this paper. Moreover, the model-based PM_2.5_ exposure products are at a coarser resolution (36 km × 36 km), compared to the satellite-derived concentrations (1 km × 1 km). The spatial resolution of the satellite-derived concentrations is more well aligned with the spatial resolution of the NFHS clusters.

### Anthropogenic and source-specific PM_2.5_ exposure

We estimated annual-averaged anthropogenic PM_2.5_ concentrations for the year 2016 using the Community Multiscale Air Quality (CMAQ) model^19^. The model set up WRF v.3.9.1^32^ & CMAQ v.5.3.1^33^ was used to estimate species-specific PM_2.5_ concentrations (elemental carbon: EC, ammonium: NH_4_, nitrate: NO_3_, organic carbon: OC, sulfate: SO_4_, soil, and others, including chloride (Cl), sodium (Na), magnesium (Mg), potassium (K), calcium (Ca), soil, and water molecules, and other unspecified species), as well as source-specific PM_2.5_ levels from agricultural residue burning (ARB), industry (IND), power (POW), transport (TRA), domestic burning (DOM), road dust (RDUST), international contributions (INT), others (OTH), that include refuse burning, construction, crematoria, NH_3_, biogenic emissions, refineries, and evaporative non-methane volatile organic compounds at a 36 km × 36 km scale^19^ (Fig. S9). We derived anthropogenic PM_2.5_ concentrations by subtracting soil dust levels from total PM_2.5_ concentrations derived from the speciated PM_2.5_ analysis. (Fig. S8). When conducting analyses involving anthropogenic PM_2.5_, and source-specific PM_2.5_ levels, we removed clusters for which we did not have information on these exposures due to issues with clipping the exposure dataset and were left with 27,535 clusters (Fig. S11). A coefficient of determination between ground-based observations and simulated monthly averaged PM_2.5_ concentrations of ~ 0.81 was reported. For more details refer to^19^. We also estimated the ratio of exposure to PM_2.5_ from power generation (POW) relative to the NFHS-4 nighttime luminosity index as a measure of inequalities of exposure to POW relative to the benefits that different consumers receive (Fig. S10).

### Statistical methods

We evaluated disparities in exposure based on local demographic characteristics. To do so, we rank ordered all clusters based on the prevalence of the different SES parameters considered in this study. We compared the distribution of pollution levels in the top and bottom decile of clusters based on each SES parameter (Fig. 1). Note, we do not present population-weighted exposures because our prevalence parameters are based on the number of households or the number of mothers in each cluster, whereas we only have data on the total population in each cluster. We evaluated high-end exposure disparities to pollution by analyzing the distribution of demographic characteristics of clusters above the 90th percentiles of air pollution exposure among all clusters and comparing it to the national distribution (Fig. 2).

We analyzed the PM_2.5_ exposures as a continuous variable, with multilevel linear models including random effects for cluster, district and state-spatial scales. First, we used null models, only including fixed effects for urban/rural to estimate the crude variation in the pollutant exposures at each geographic level. The proportion of variance attributed to each level, z, was computed as follows: 100 × var_z_/(var_cluster_ + var_district_ + var_state_). We next added the logarithm of population density to our model and repeated this calculation.

We then used multilevel regression models, again only including urban/rural fixed effects and the logarithm of population density, using each of the PM exposures as the outcome and each SES variable as the exploratory parameters to evaluate associations between pollution and SES. We report the % variance change at each level from introducing the SES variables into the models.

We then ran fully adjusted models where we evaluated associations between the exposures of interest and SES factors after also adjusting for all other SES parameters. In all models, we scaled all independent variables by using z-scores to present effect estimates of linear associations per one standard deviation (SD) increase and facilitate comparability of estimates across all variables used. In the fully-adjusted models, we did not include the prevalence of literate mothers in the analysis, to ensure that the variance inflation factors of all coefficients included were less than four. We also report results from this analysis, disaggregated by urban/rural designation.

We tested for potential non-linearities between the exposures of interest and each SES under consideration and time of operation in the following manner: We used penalized splines (p-spline) to flexibly model the associations between the exposures of interest and the SES under consideration in the fully-adjusted model using a generalized additive model (GAM). We used the minimized generalized cross-validation score (GCV) criterion to select the optimal degrees of freedom (df). We plotted the relationships observed. The GAM fitting and analysis were conducted with the mgcv package in the programming language R.

We used fully-adjusted models to evaluate associations between the ratio of PM_2.5_ concentrations from power generation and the average nighttime luminosity (as a proxy for the benefits from power-generation) and each SES parameter. In this manner we evaluated the variation in exposure to concentrations from an important source, relative to benefits received across different SES levels. Finally, we evaluated associations between the difference in PM_2.5_ concentrations between the years 2015 and 2010, and the percentage difference with each SES parameter considered using fully-adjusted models. We repeated these analyses using data from urban and rural clusters, separately.

We mapped the geographic distribution of all analyzed variables. All models were run in R 4.2.1. Maps were plotted using QGIS 3.10.1.

### Supplementary Information

Supplementary Information.

## Data Availability

NFHS-4 data is available on submitting a request via the DHS website https://dhsprogram.com/.
