# Author Correction: An environmental justice analysis of air pollution in India

**DOI:** 10.1038/s41598-023-46982-4

**Published:** 2023-11-09

**Authors:** Priyanka N. deSouza, Ekta Chaudhary, Sagnik Dey, Soohyeon Ko, Jeremy Németh, Sarath Guttikunda, Sourangsu Chowdhury, Patrick Kinney, S. V. Subramanian, Michelle L. Bell, Rockli Kim

**Affiliations:** 1 Department of Urban and Regional Planning, University of Colorado Denver, Denver, CO USA; 2 Centre for Atmospheric Sciences, Indian Institute of Technology (IIT) Delhi, New Delhi, India; 3 Centre of Excellence for Research on Clean Air, IIT Delhi, New Delhi, India; 4 School of Public Policy, IIT Delhi, New Delhi, India; 5 Department of Public Health Sciences, Graduate School of Korea University, Seoul, South Korea; 6 Interdisciplinary Program in Precision Public Health, Department of Public Health Sciences, Graduate School of Korea University, Seoul, South Korea; 7 Transportation Research and Injury Prevention (TRIP) Centre, Indian Institute of Technology, New Delhi, 110016 India; 8 Urban Emissions, New Delhi, 110019 India; 9 CICERO Center for International Climate Research, Oslo, Norway; 10 School of Public Health, Boston University, Boston, MA USA; 11 Harvard Center for Population and Development Studies, Bow Street, Cambridge, MA 02138 USA; 12 Department of Social and Behavioral Sciences, Harvard T.H. Chan School of Public Health, 677 Huntington Avenue, Boston, MA 02115 USA; 13 School of the Environment, Yale University, New Haven, CT USA; 14 Division of Health Policy and Management, College of Health Science, Korea University, Seoul, South Korea

Correction to: *Scientific Reports* 10.1038/s41598-023-43628-3, published online 04 October 2023

The original version of this Article contained an error in the spelling of the author Sagnik Dey which was incorrectly given as Sagnk Dey.

The original Article has been corrected.

