# Supplementary material for: An environmental justice analysis of air pollution in India
Source: Sci Rep. 2023 Oct 4;13:16690. doi: 10.1038/s41598-023-43628-3 (PMC10551031; doi:10.1038/s41598-023-43628-3)
Supplement: Supplementary file 1 — Supplementary Information. [file 41598_2023_43628_MOESM1_ESM.pdf]

Supplementary Information for An Environmental Justice Analysis of Air Pollution in India

Priyanka N deSouza[1,2*], Ekta Chaudhary[2], Sagnk Dey[2,3,4], Soohyeon Ko[5,6], Jeremy Németh[1], Sarath Guttikunda[7,8], Sourangsu Chowdhury[9], Patrick Kinney[10], SV Subramanian[11,12], Michelle L Bell[13], Rockli Kim[5,6,11]

1: Department of Urban and Regional Planning, University of Colorado Denver, CO, USA
2: Centre for Atmospheric Sciences, Indian Institute of Technology (IIT) Delhi, New Delhi, India
3: Centre of Excellence for Research on Clean Air, IIT Delhi, New Delhi, India
4: School of Public Policy, IIT Delhi, New Delhi, India
5: Department of Public Health Sciences, Graduate School of Korea University, Seoul, South Korea
6: Interdisciplinary Program in Precision Public Health, Department of Public Health Sciences, Graduate School of Korea University, Seoul, South Korea
7: Transportation Research and Injury Prevention (TRIP) Centre, Indian Institute of Technology, New Delhi 110016, India
8: Urban Emissions, New Delhi 110019, India
9: CICERO Center for International Climate Research, Oslo, Norway
10: Boston University School of Public Health, Boston, MA
11: Harvard Center for Population and Development Studies, Bow Street, Cambridge, MA, 02138, USA
12: Department of Social and Behavioral Sciences, Harvard T.H. Chan School of Public Health, 677 Huntington Avenue, Boston, MA, 02115, USA
13: School of the Environment, Yale University, New Haven, CT, USA

*: Corresponding author (priyanka.desouza@ucdenver.edu)

S1: Maps of SES parameters and PM exposures considered

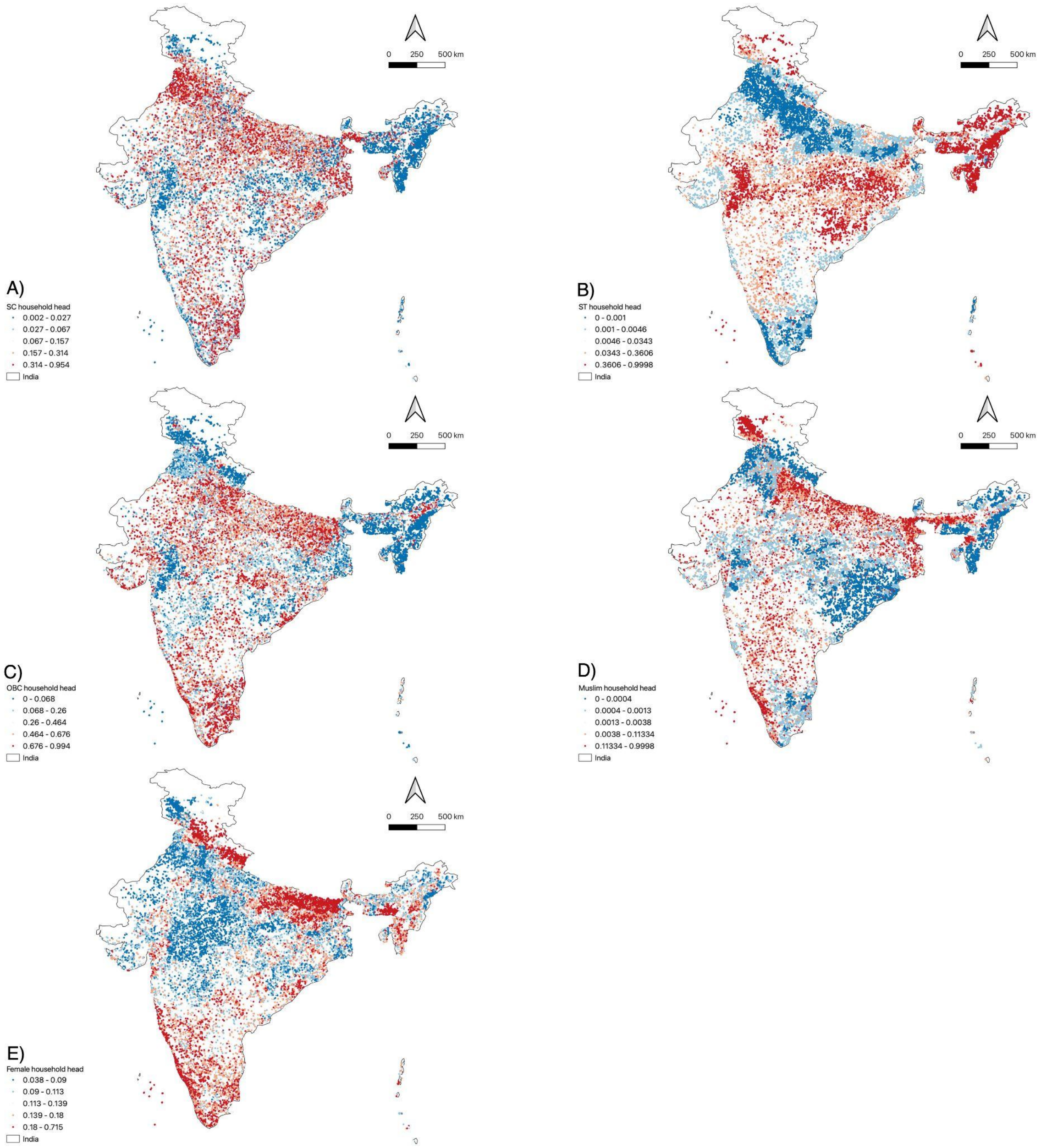

Figure S1*: The prevalence of households with an A) SC household head, B) ST household head, C) OBC household head, D) Muslim household head, E) Female household head classified by quintiles across India. Maps were generated using QGIS 3.28*[1]

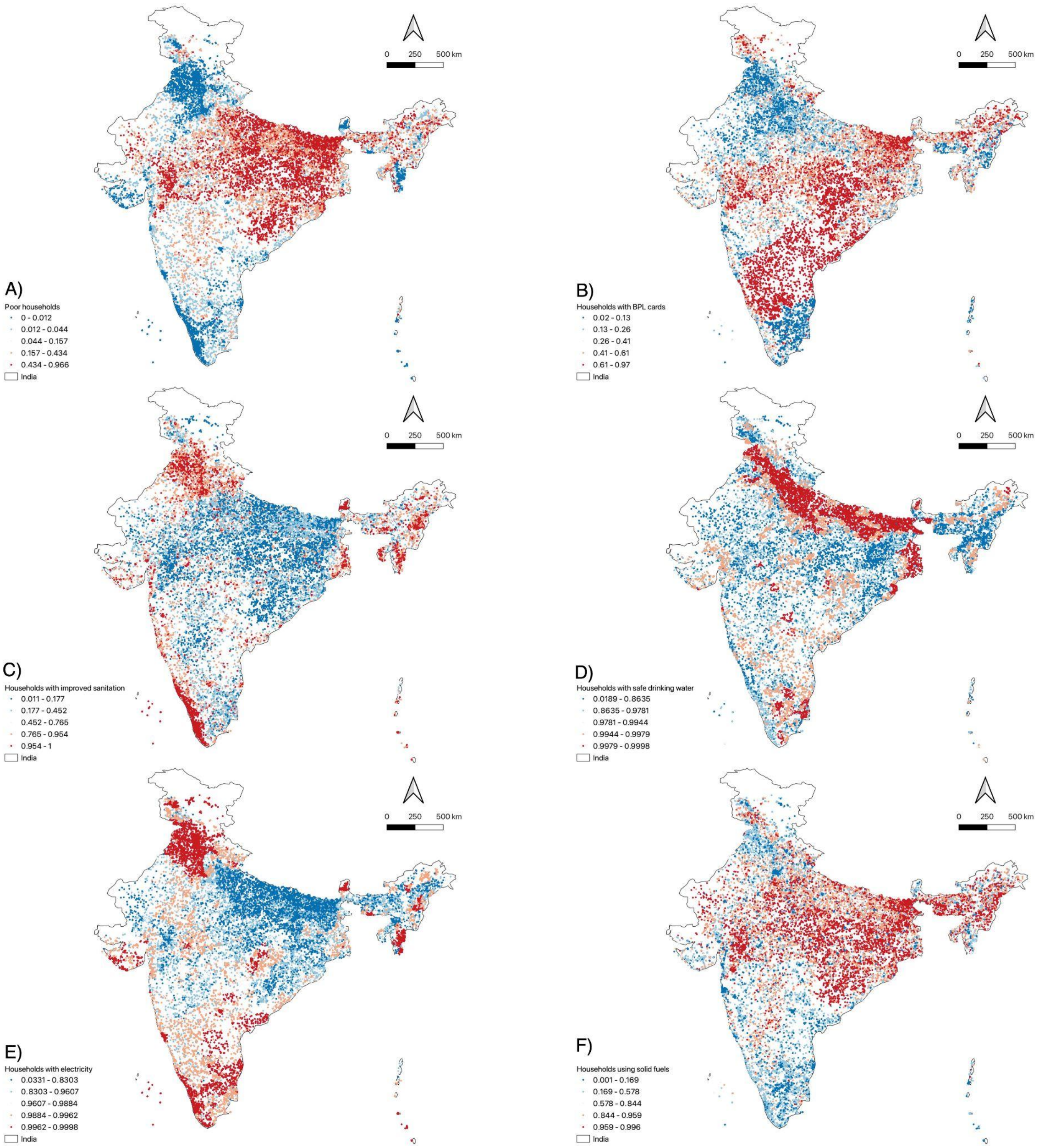

Figure S2*: The prevalence of households which: A) Were Poor, B) Had a BPL card, C) Had Improved Sanitation, D) Had access to safe drinking water, E) Had access to Electricity, F) Used solid fuels, classified by quintiles across India. Maps were generated using QGIS 3.28*[1]

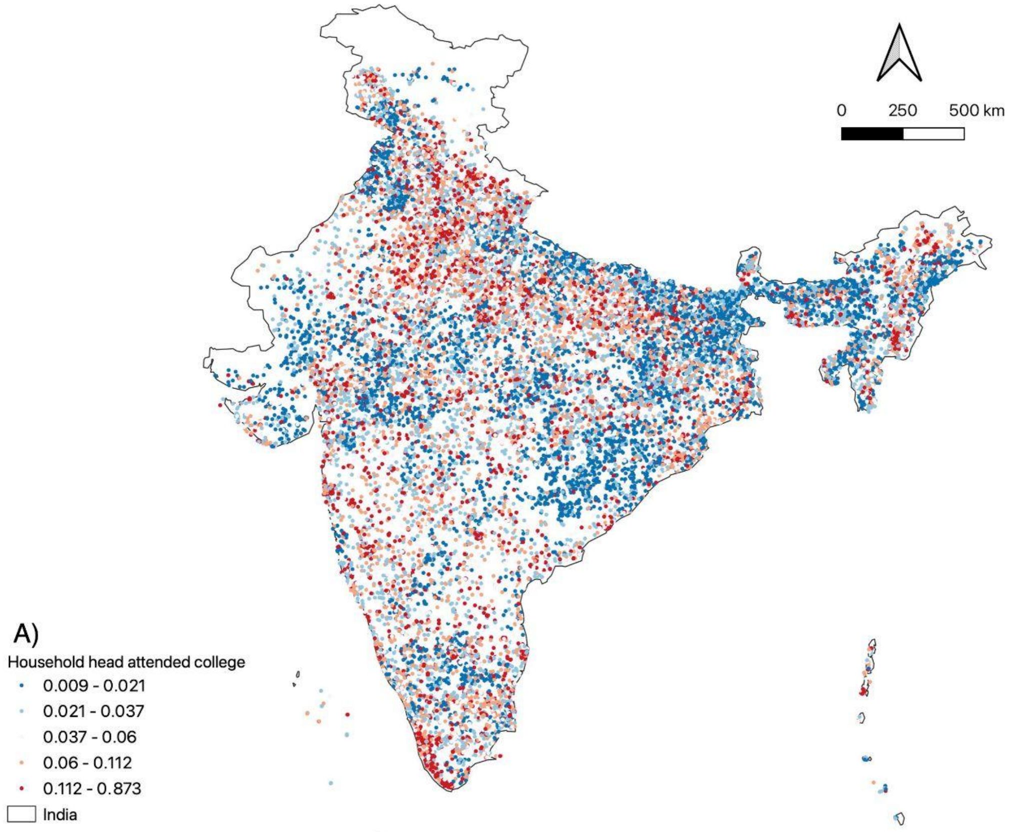
0
250
500 km
A)
Household head attended college
0.009 - 0.021
0.021 - 0.037
0.037 - 0.06
0.06 - 0.112
0.112 - 0.873
India

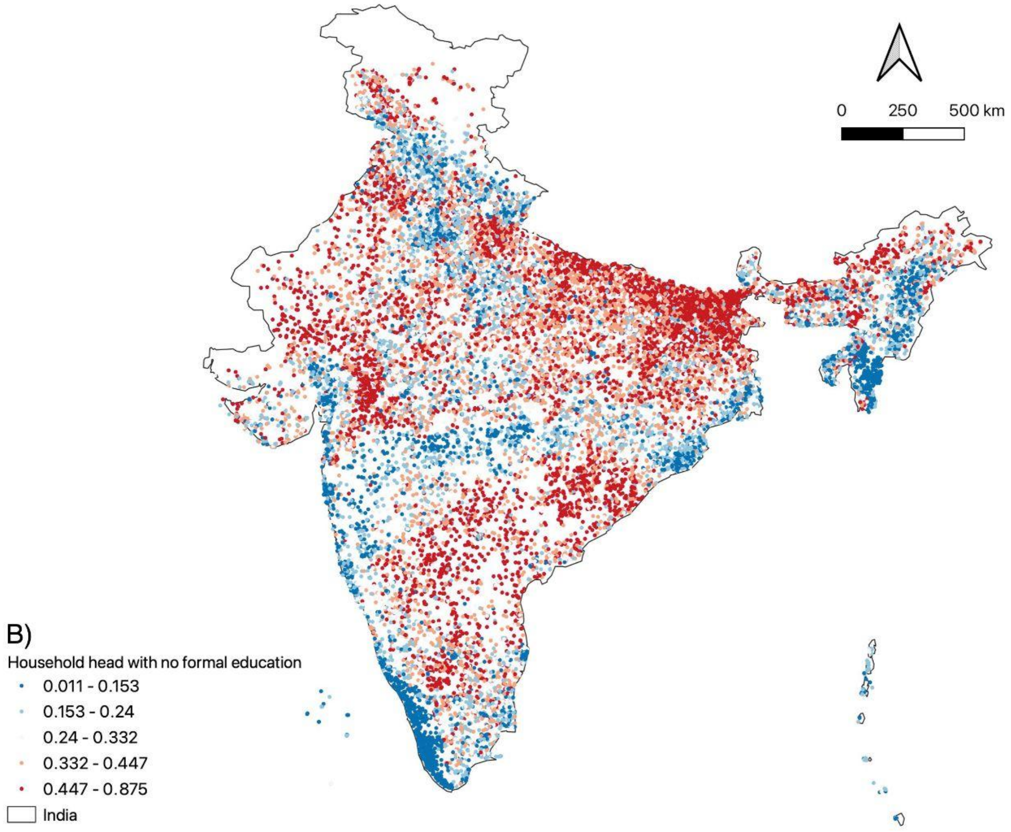
0
250
500 km
B)
Household head with no formal education
0.011 - 0.153
0.153 - 0.24
0.24 - 0.332
0.332 - 0.447
0.447 - 0.875
India

Figure S3*: The prevalence of households where: A) Household head was college educated, B) Household head had no formal education, classified by quintiles across India. Maps were generated using QGIS 3.28*[1]

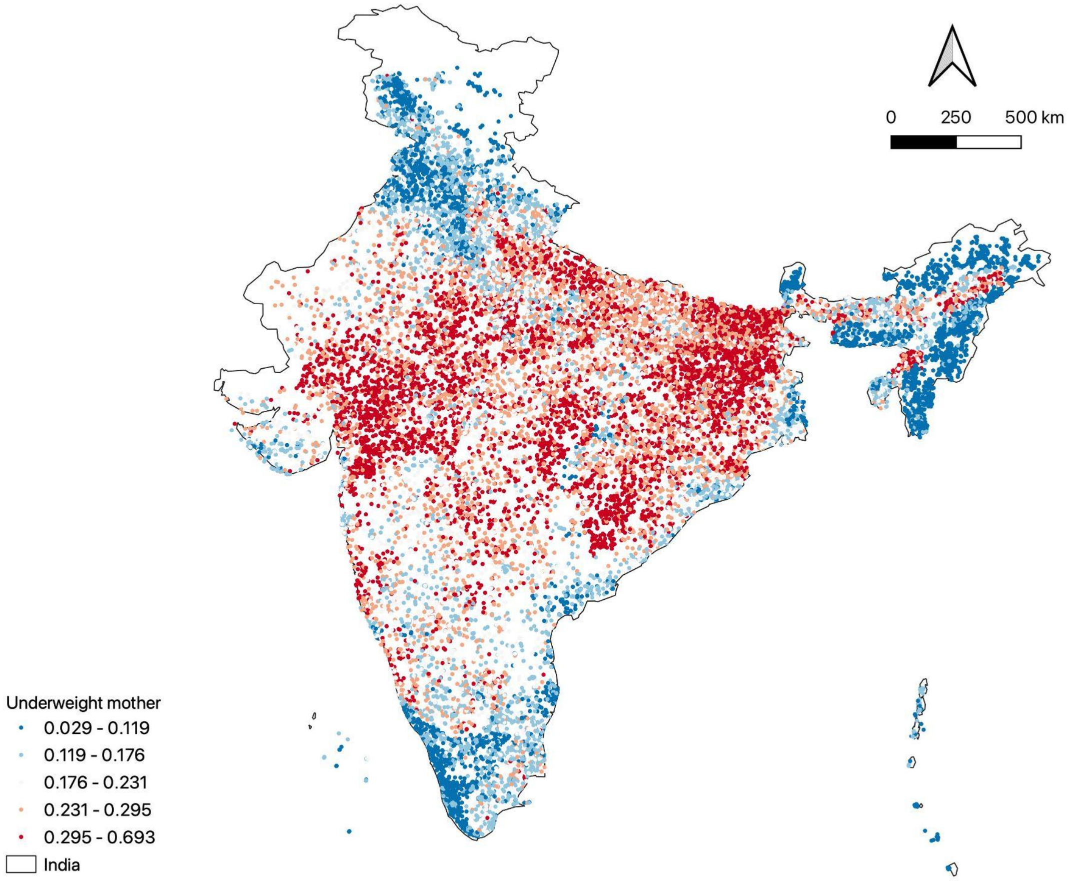

Figure S4*: The prevalence of households where the mother was thin (or underweight), classified by quintiles across India. The map was generated using QGIS 3.28*[1]

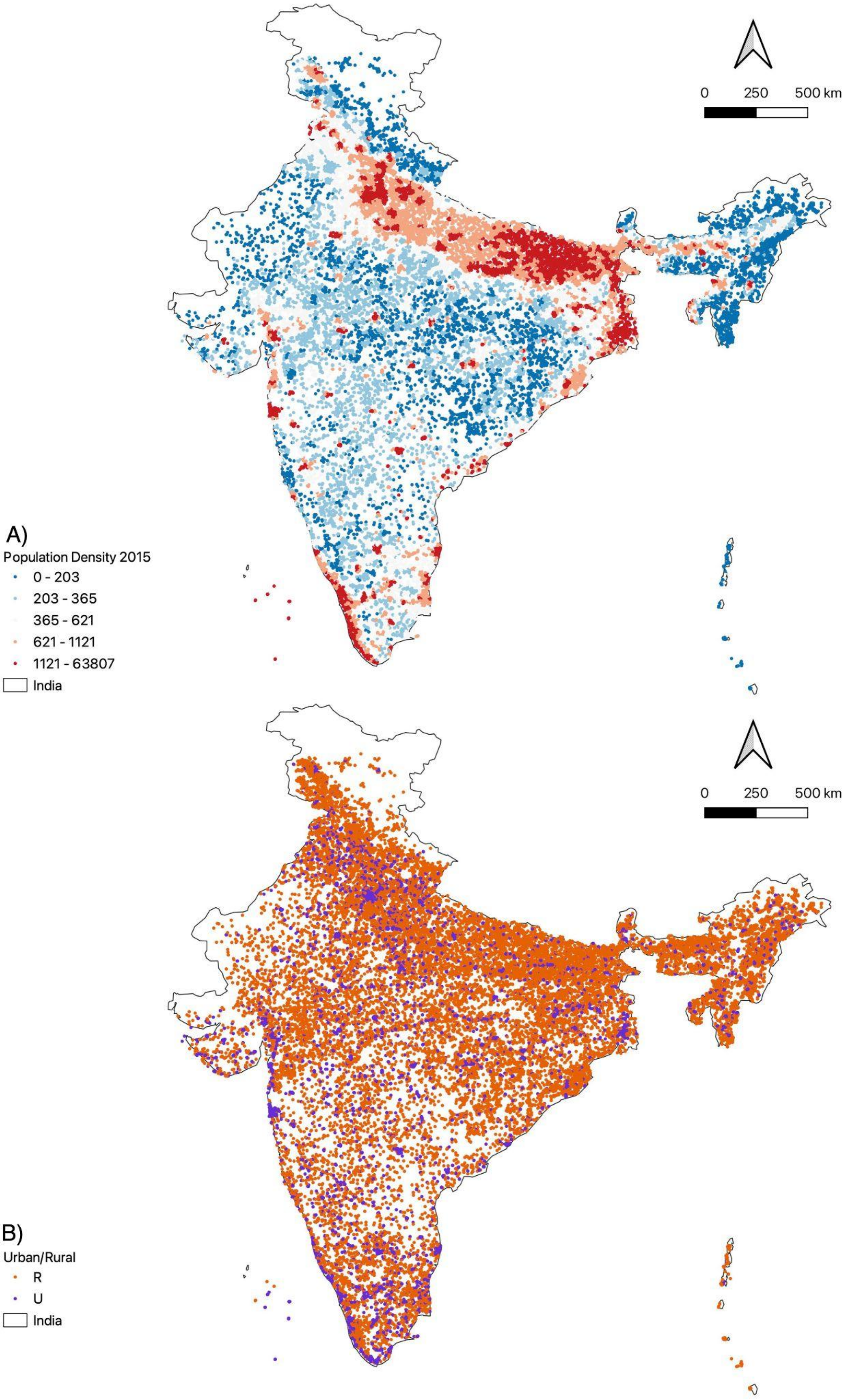
0
250
500 km
A)
Population Density 2015
0 - 203
203 - 365
365 - 621
621 - 1121
1121 - 63807
India
0
250
500 km
B)
Urban/Rural
R
U
India

Figure S5*: A) Population Density classified by decile and B) Urban (U)/Rural (R) clusters. Maps were generated using QGIS 3.28*[1]

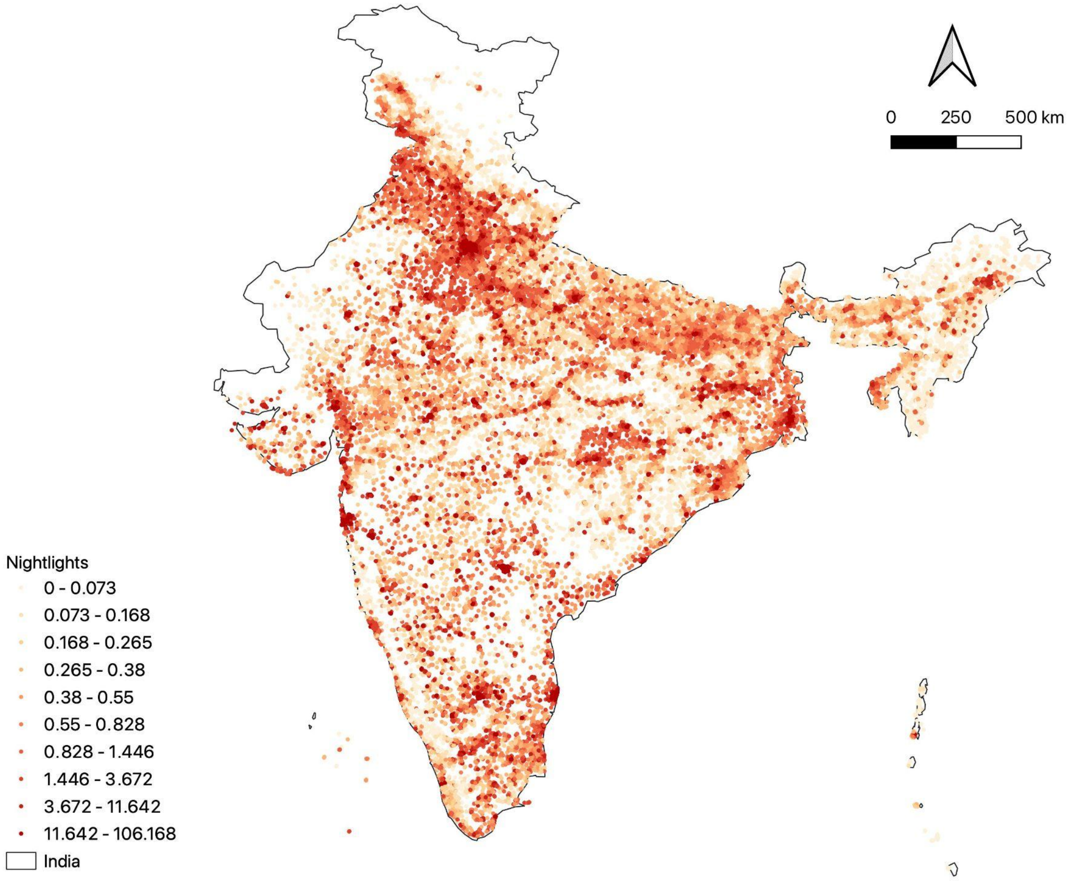

Figure S6*: Nightlight Index categorized by decile. The map was generated using QGIS 3.28*[1]

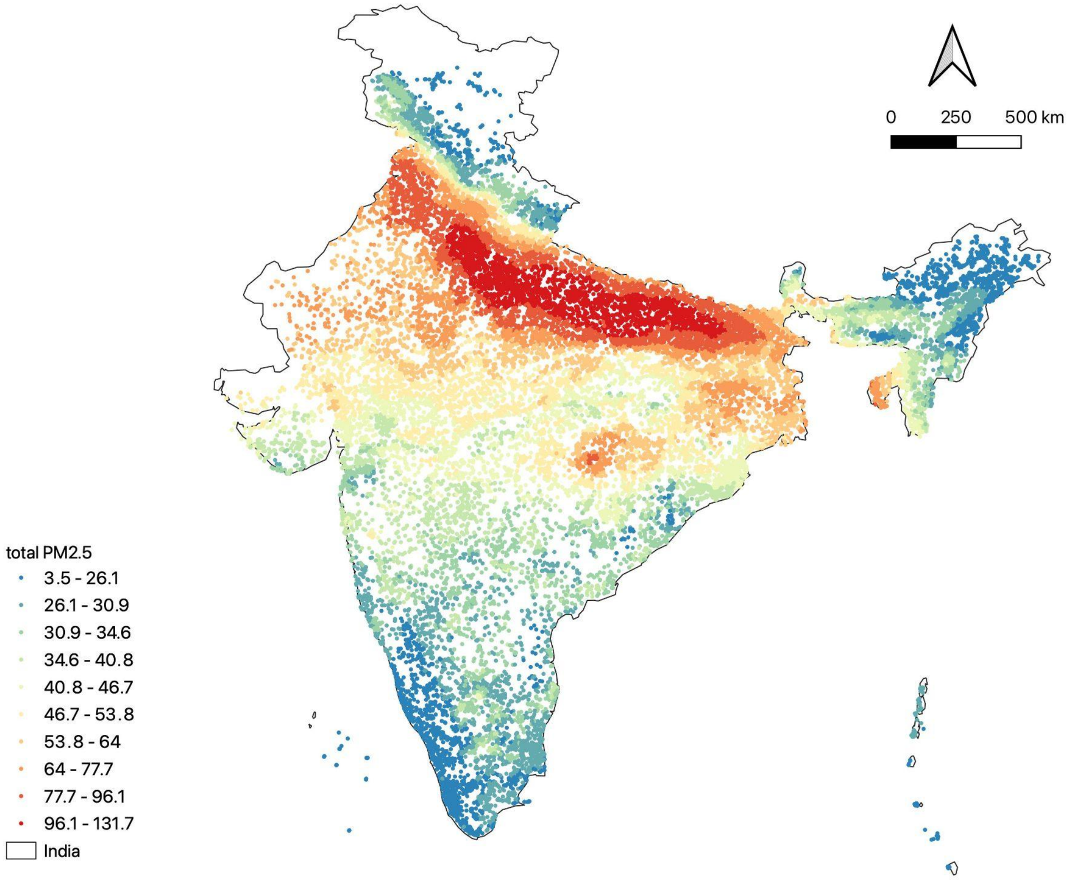

Figure S7*: Average $PM_{2.5}$ levels for the years 2010-2015 classified by decile for each DHS cluster. The map was generated using QGIS 3.28*[1]

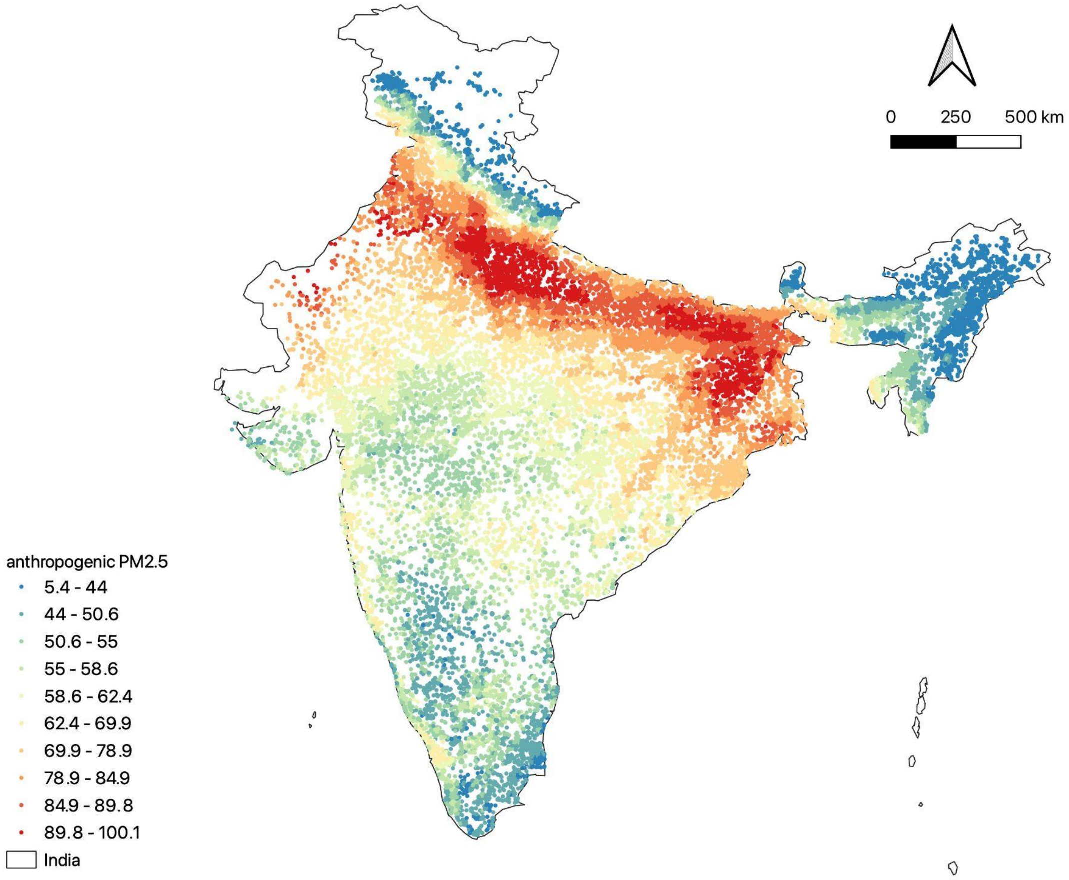

Figure S8: *Average anthropogenic $PM_{2.5}$ levels for the year 2016 classified by decile for each DHS cluster. The map was generated using QGIS 3.28*[1]

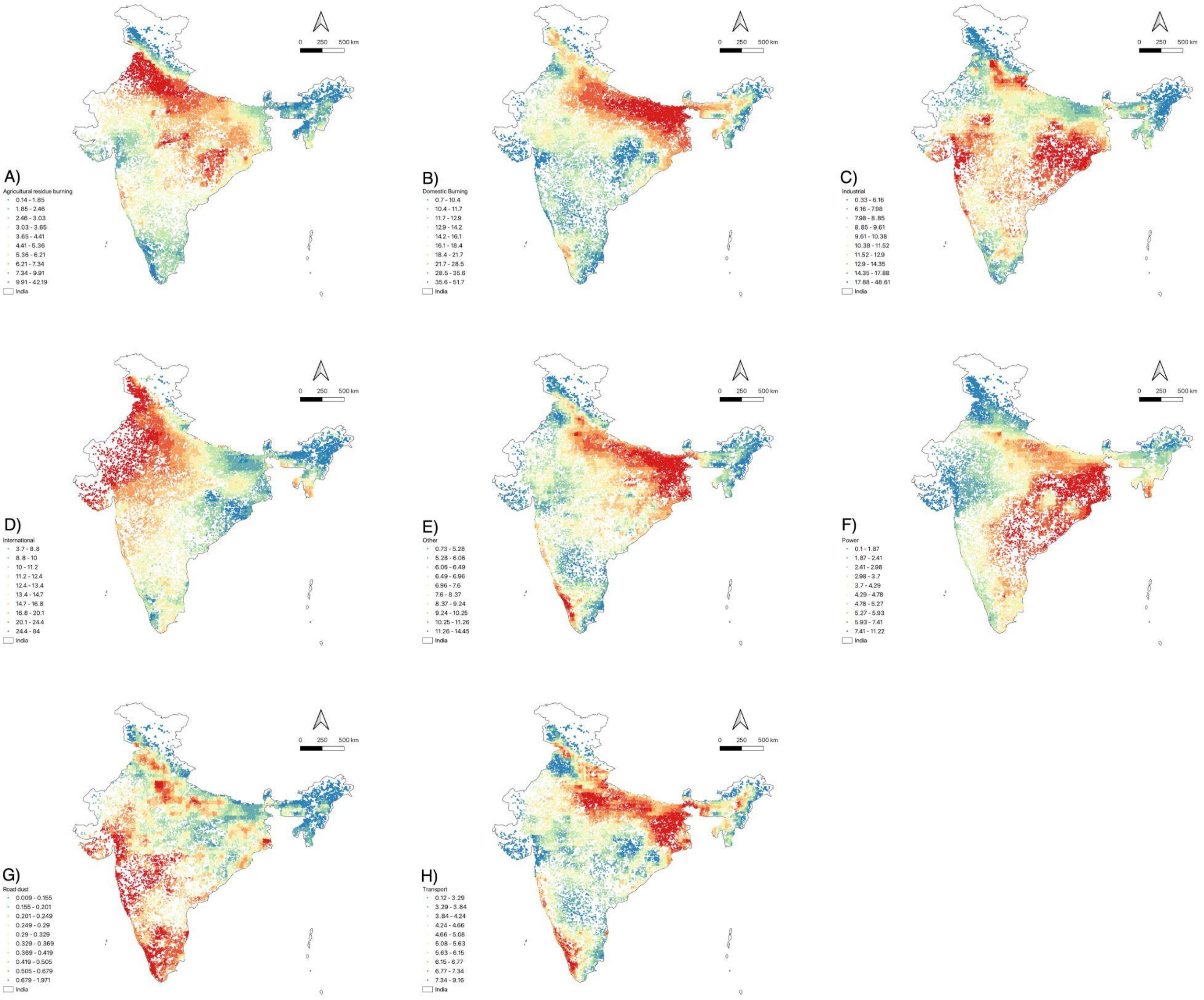

Figure S9*: Average source-specific $PM_{2.5}$ levels from A) Agricultural Residue Burning, B) Domestic Burning, C) Industrial sources, D) International sources, E) Other, F) Power, G) Road Dust, H) Transport for the year 2016 classified by decile for each DHS cluster. Maps were generated using QGIS 3.28*[1]

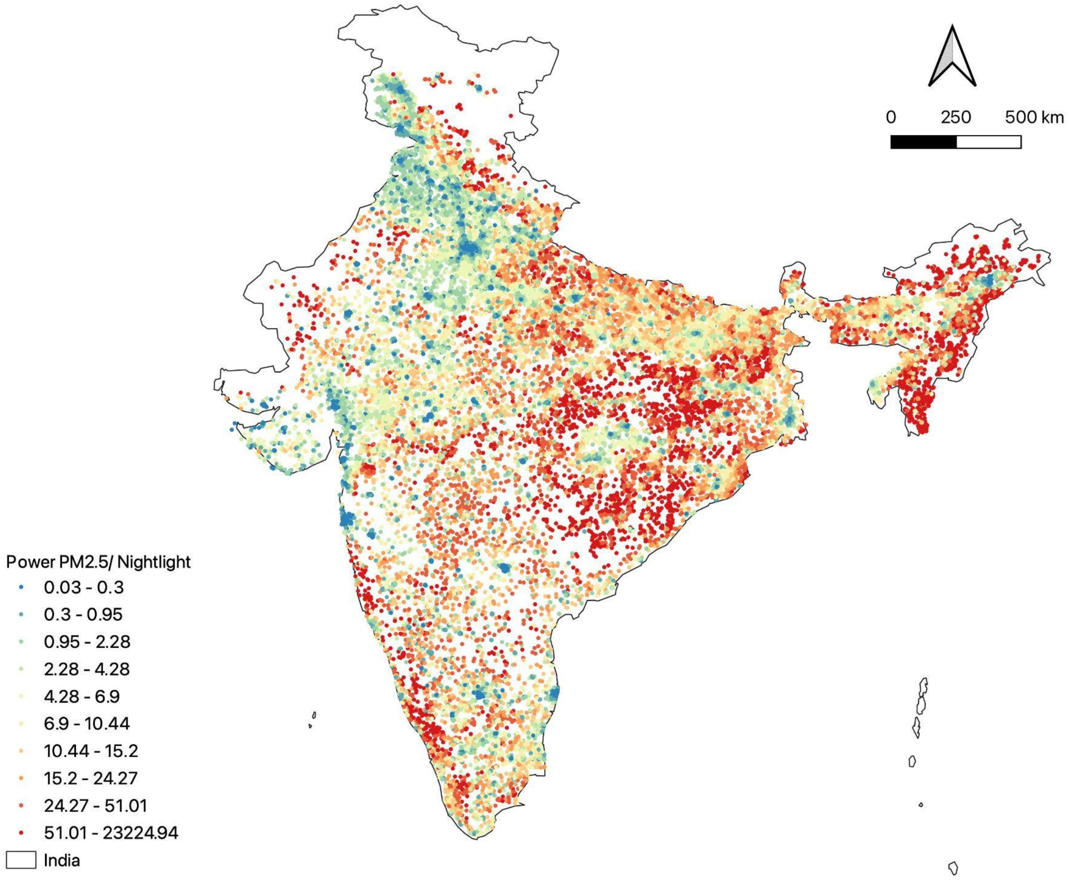

***Figure S10**: Power-PM$_{2.5}$ by Nightlight Index categorized by decile. The map was generated using QGIS 3.28*[1]

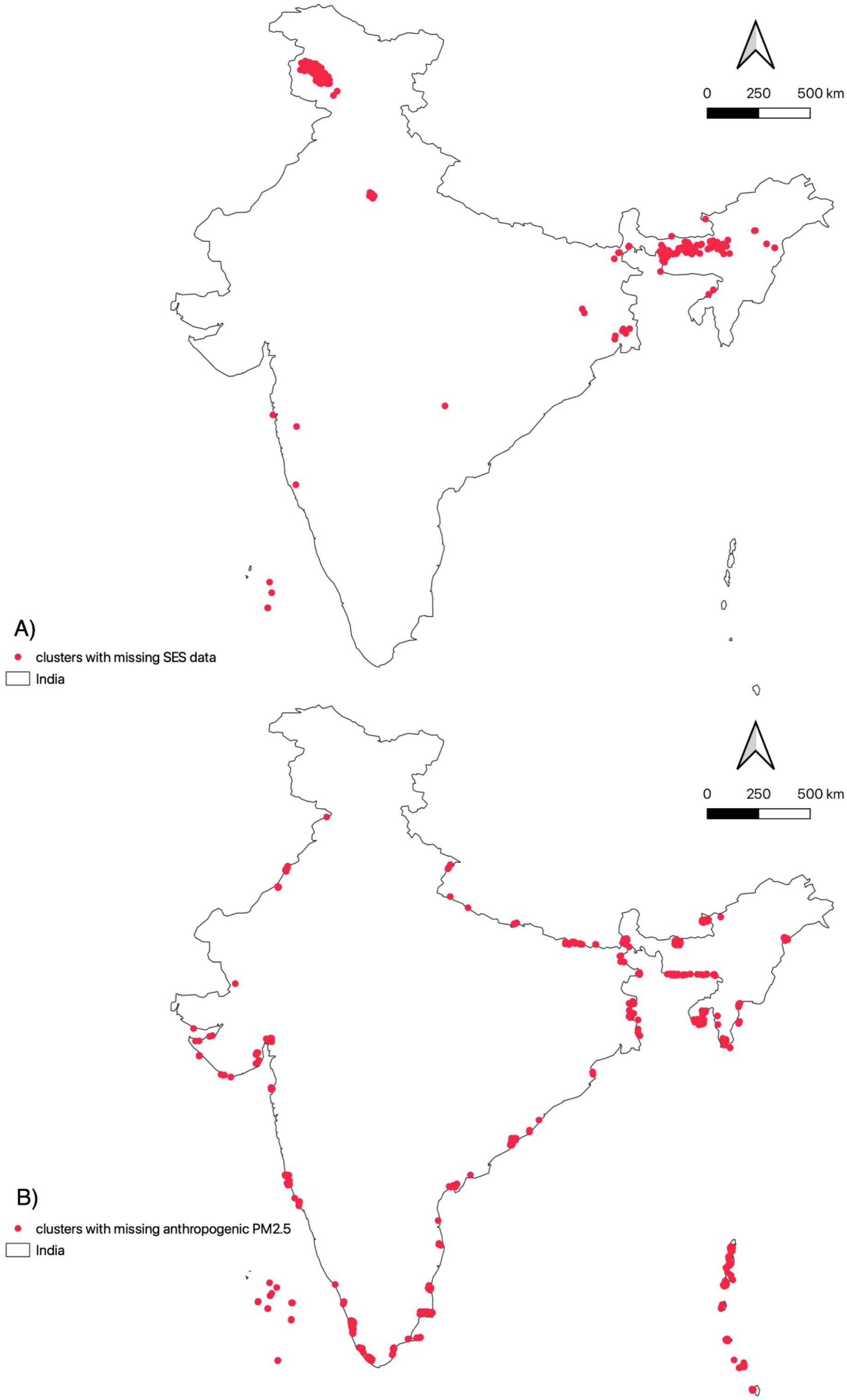

Figure S11: *Locations of clusters with missing data on A) SES covariates or population density, and B) anthropogenic and source-specific $PM_{2.5}$ levels. Maps were generated using QGIS 3.28*[1]

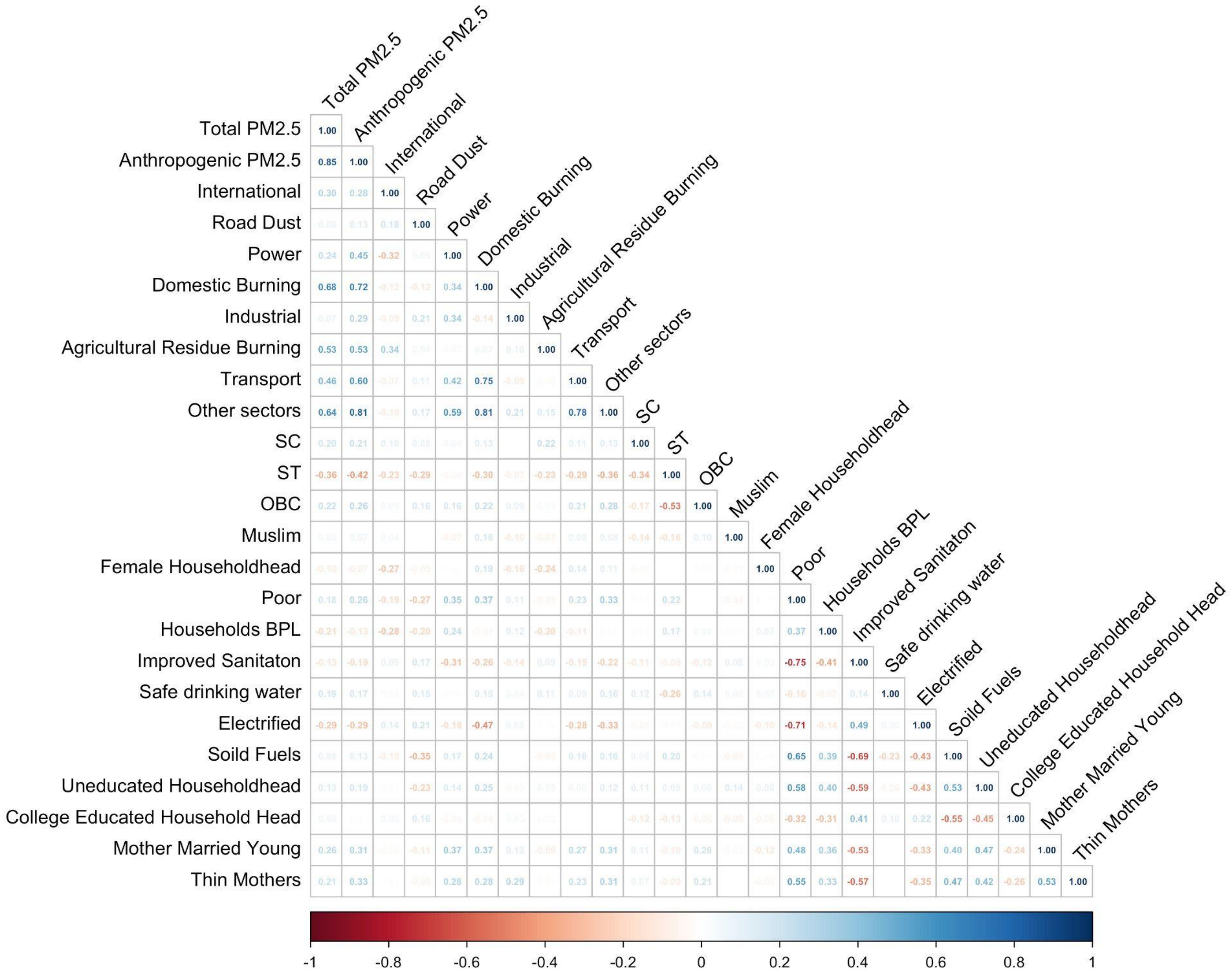

***Figure S12**: Pair-wise Pearson correlations between each of the SES variables and total, anthropogenic, and source-specific $PM_{2.5}$ considered in this study*

S2: Variation of source-specific PM exposures by clusters categorized into decile on the basis of the prevalence of different SES variables

There were small absolute differences in industrial $PM_{2.5}$ concentrations across clusters categorized into deciles by the prevalence of different SES parameters, we observed that industrial-$PM_{2.5}$ levels were lowest in clusters having the lowest prevalence of SC (Decile 10: 11.1 μg/m^3, Decile 1: 8.8 μg/m^3), OBC household heads (Decile 10: 11.0 μg/m^3, Decile 1: 6.9cμg/m^3), and underweight mothers (Decile 10: 13.1 μg/m^3, Decile 1: 7.2 μg/m^3). We observed the opposite trend for clusters with the lowest prevalence of ST households heads (Decile 10: 8.7 μg/m^3, Decile 1: 11.0 μg/m^3) (**Figure S14**).

Power-$PM_{2.5}$ also showed relatively small absolute increases over clusters with increasing proportions of poor households (Decile 10: 5.9 μg/m^3, Decile 1: 3.2 μg/m^3), households living BPL (Decile 10: 5.6 μg/m^3, Decile 1: 3.8 μg/m^3), households using solid fuels (Decile 10: 5.6 μg/m^3, Decile 1: 4.0 μg/m^3), and underweight mothers (Decile 10: 5.1 μg/m^3, Decile 1: 3.2 μg/m^3). Power-$PM_{2.5}$ levels were lower in clusters with the decreasing prevalence of households with improved sanitation (Decile 10: 3.5 μg/m^3, Decile 1: 5.7 μg/m^3), and electrified households (Decile 10: 3.1 μg/m^3, Decile 1: 5.1 μg/m^3) (**Figure S15**).

We observed that unsurprisingly, $PM_{2.5}$ from domestic burning was lowest in clusters with the highest prevalence of electrified households (Decile 10: 15.0 μg/m^3, Decile 1: 30.5 μg/m^3). Domestic burning-$PM_{2.5}$ concentrations were the highest in clusters with the highest prevalence of mothers married young (Decile 10: 24.7 μg/m^3, Decile 1: 14.4 μg/m^3), poor households (Decile 10: 25.5 μg/m^3, Decile 1: 15.5 μg/m^3), households with access to safe drinking water (Decile 10: 29.0 μg/m^3, Decile 1: 16.4 μg/m^3), Muslim households heads (Decile 10: 23.7 μg/m^3, Decile 1: 12.8 μg/m^3), and SC households (Decile 10: 20.9 μg/m^3, Decile 1: 12.3 μg/m^3) (**Figure S16**).

We observed little absolute variation in $PM_{2.5}$ levels across different clusters from agricultural residue burning, road dust, and international sources (**Figures S17, S18** and **S19**). $PM_{2.5}$ from these sources were lowest in clusters with the highest prevalence of poor residents (Agricultural Residue Burning: Decile 10: 5.4 μg/m^3, Decile 1: 11.1 μg/m^3; Road Dust: Decile 10: 0.3 μg/m^3, Decile 1: 0.5 μg/m^3; International: Decile 10: 13.0 μg/m^3, Decile 1: 18.6 μg/m^3). However, $PM_{2.5}$ from these sources were highest in clusters with the highest prevalence of SC residents (Agricultural Residue Burning: Decile 10: 7.9 μg/m^3, Decile 1: 2.7 μg/m^3; Road Dust: Decile 10: 0.4 μg/m^3, Decile 1: 0.2 μg/m^3; International: Decile 10: 16.6 μg/m^3, Decile 1: 11.6μg/m^3).

Transport-related $PM_{2.5}$ levels were highest in clusters with a higher prevalence of SC (Decile 10: 5.4 μg/m^3, Decile 1: 4.1 μg/m^3), OBC (Decile 10: 5.5 μg/m^3, Decile 1: 4.1 μg/m^3), Muslim

(Decile 10: 5.5 μg/m^3, Decile 1: 4.2 μg/m^3), female-headed household heads (Decile 10: 5.8 μg/m^3, Decile 1: 4.9 μg/m^3), households using solid fuels (Decile 10: 5.6 μg/m^3, Decile 1: 4.9 μg/m^3), underweight mothers (Decile 10: 5.6 μg/m^3, Decile 1: 4.3 μg/m^3), and mothers who married young (Decile 10: 5.9 μg/m^3, Decile 1: 4.8 μg/m^3). Transport-related $PM_{2.5}$ concentrations were lowest for clusters with a higher prevalence of households BPL (Decile 10: 4.4 μg/m^3, Decile 1: 5.0 μg/m^3) electrified households (Decile 10: 4.5 μg/m^3, Decile 1: 6.2 μg/m^3), households with an ST head (Decile 10: 4.2 μg/m^3, Decile 1: 5.3 μg/m^3) (**Figure S20**).

$PM_{2.5}$ concentrations from other sources displayed similar trends to total $PM_{2.5}$ concentrations (**Figure S21**).

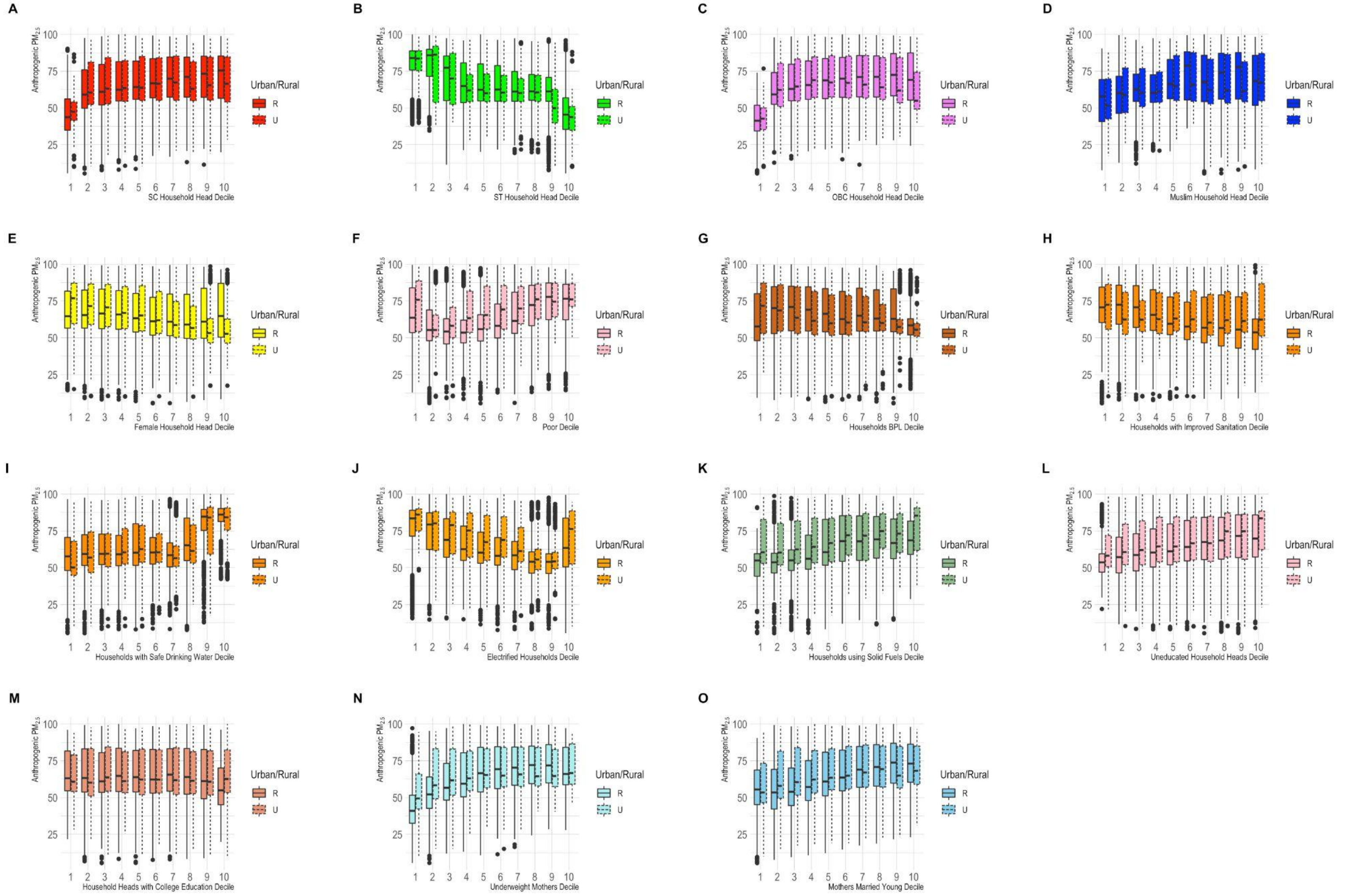

***Figure S13**: Anthropogenic $PM_{2.5}$ concentrations by decile of the different SES prevalence parameters, disaggregated by urban/rural clusters. $PM_{2.5}$ concentrations corresponding to the first and tenth decile are highlighted. The boxes correspond to the first and third quartiles of the distribution of $PM_{2.5}$ concentrations corresponding to each group.*

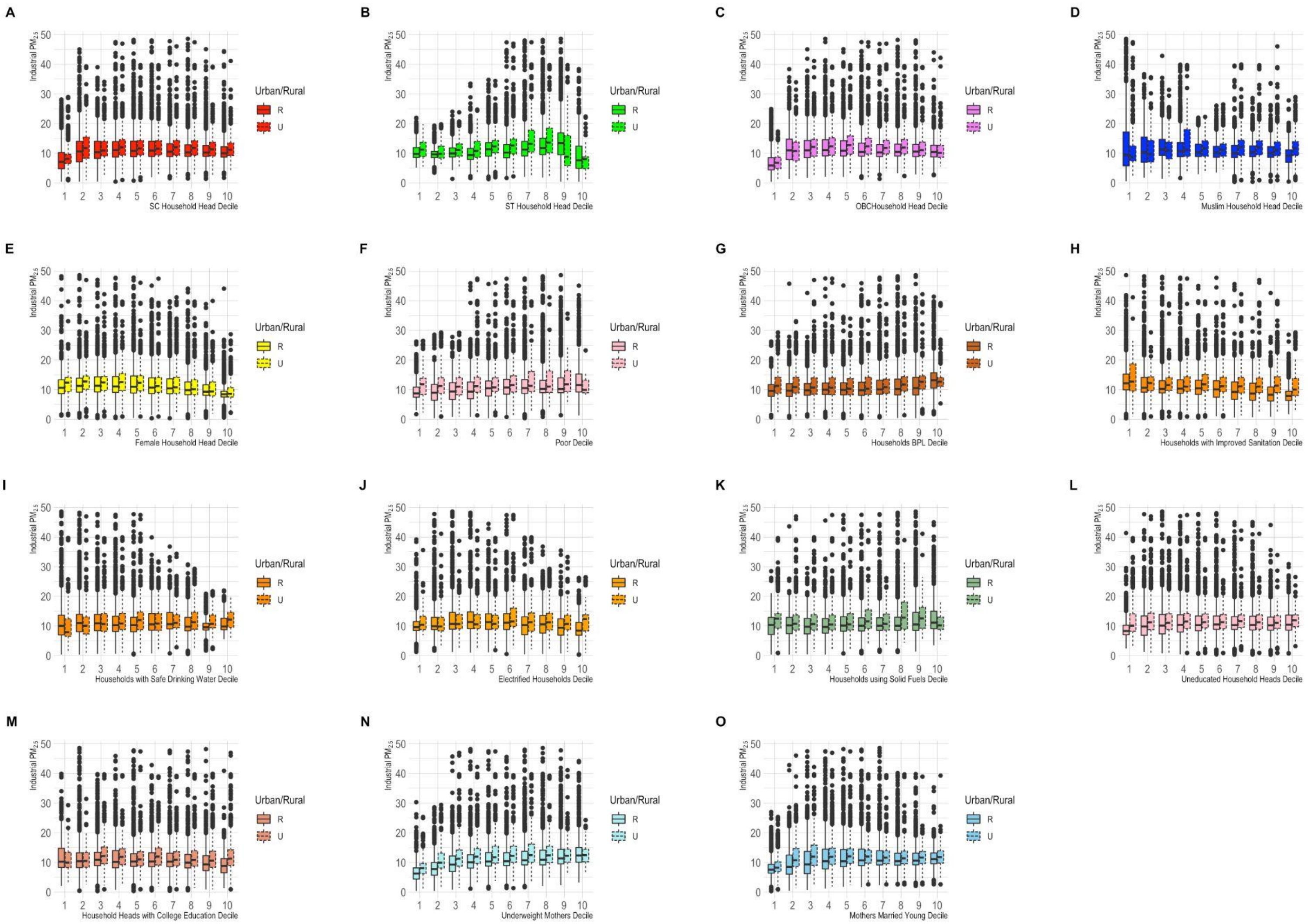

***Figure S14**: Industrial-$PM_{2.5}$ concentrations by decile of the different SES prevalence parameters, disaggregated by urban/rural clusters. Industrial-$PM_{2.5}$ concentrations corresponding to the first and tenth decile are highlighted. The boxes correspond to the first and third quartiles of the distribution of industrial-$PM_{2.5}$ concentrations corresponding to each group.*

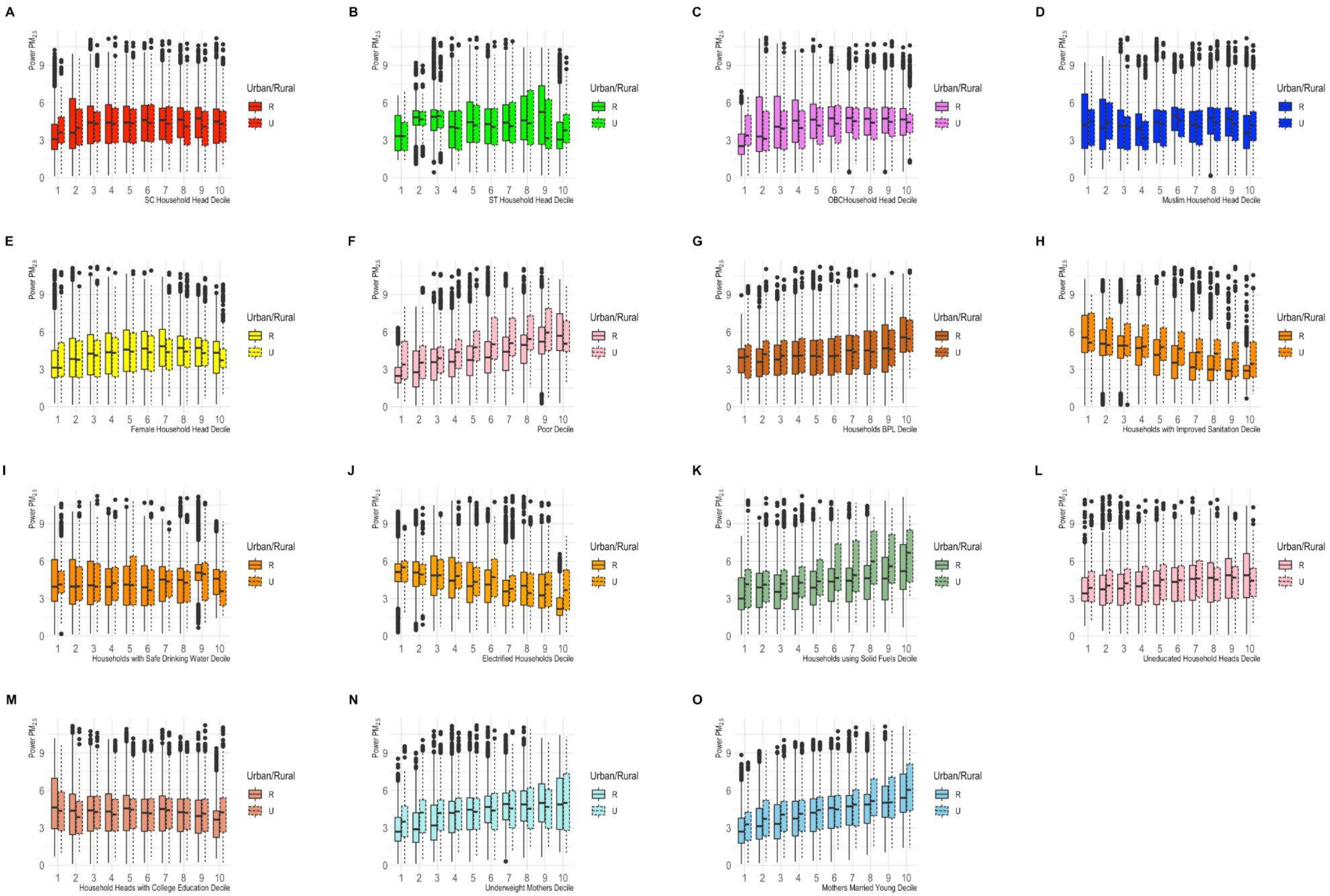

***Figure S15**: Power-$PM_{2.5}$ concentrations by decile of the different SES prevalence parameters, disaggregated by urban/rural clusters. Power-$PM_{2.5}$ concentrations corresponding to the first and tenth decile are highlighted. The boxes correspond to the first and third quartiles of the distribution of power-$PM_{2.5}$ concentrations corresponding to each group.*

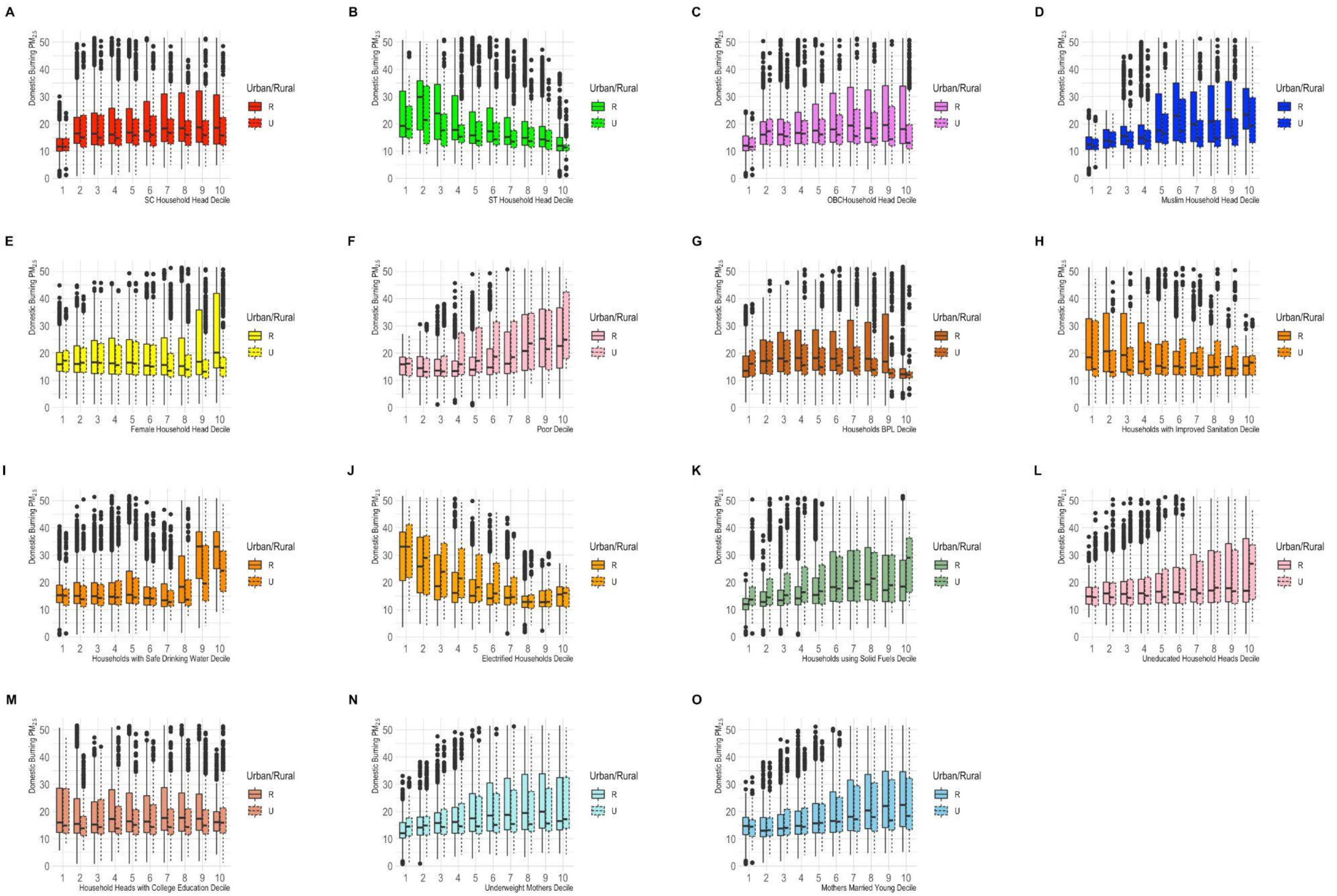

***Figure S16**: Domestic burning-PM$_{2.5}$ concentrations by decile of the different SES prevalence parameters, disaggregated by urban/rural clusters. Domestic burning-PM$_{2.5}$ concentrations corresponding to the first and tenth decile are highlighted. The boxes correspond to the first and third quartiles of the distribution of domestic burning-PM$_{2.5}$ concentrations corresponding to each group.*

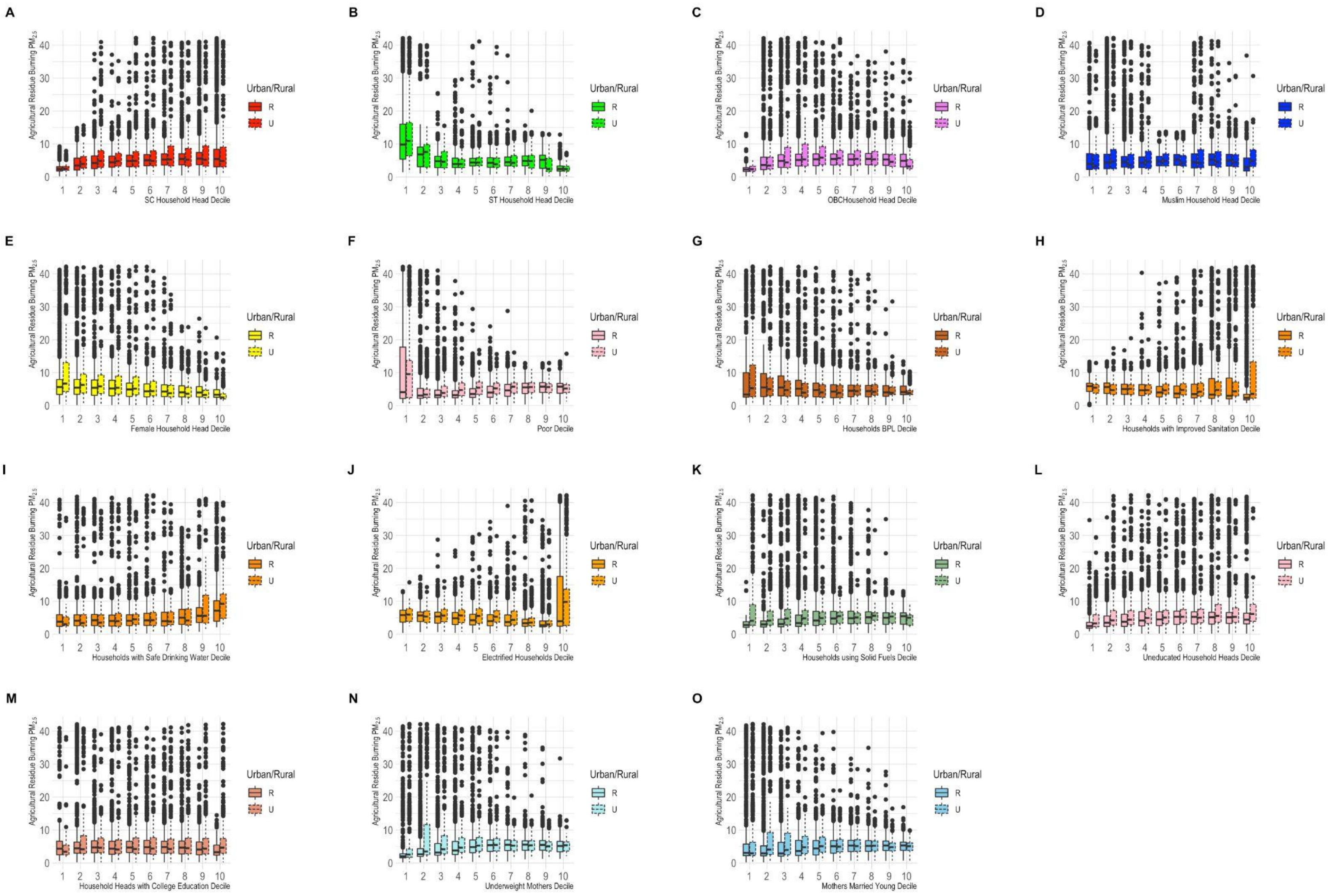

***Figure S17**: Agricultural residue burning-PM$_{2.5}$ concentrations by decile of the different SES prevalence parameters, disaggregated by urban/rural clusters. Agricultural residue burning-PM$_{2.5}$ concentrations corresponding to the first and tenth decile are highlighted. The boxes correspond to the first and third quartiles of the distribution of agricultural residue burning-PM$_{2.5}$ concentrations corresponding to each group.*

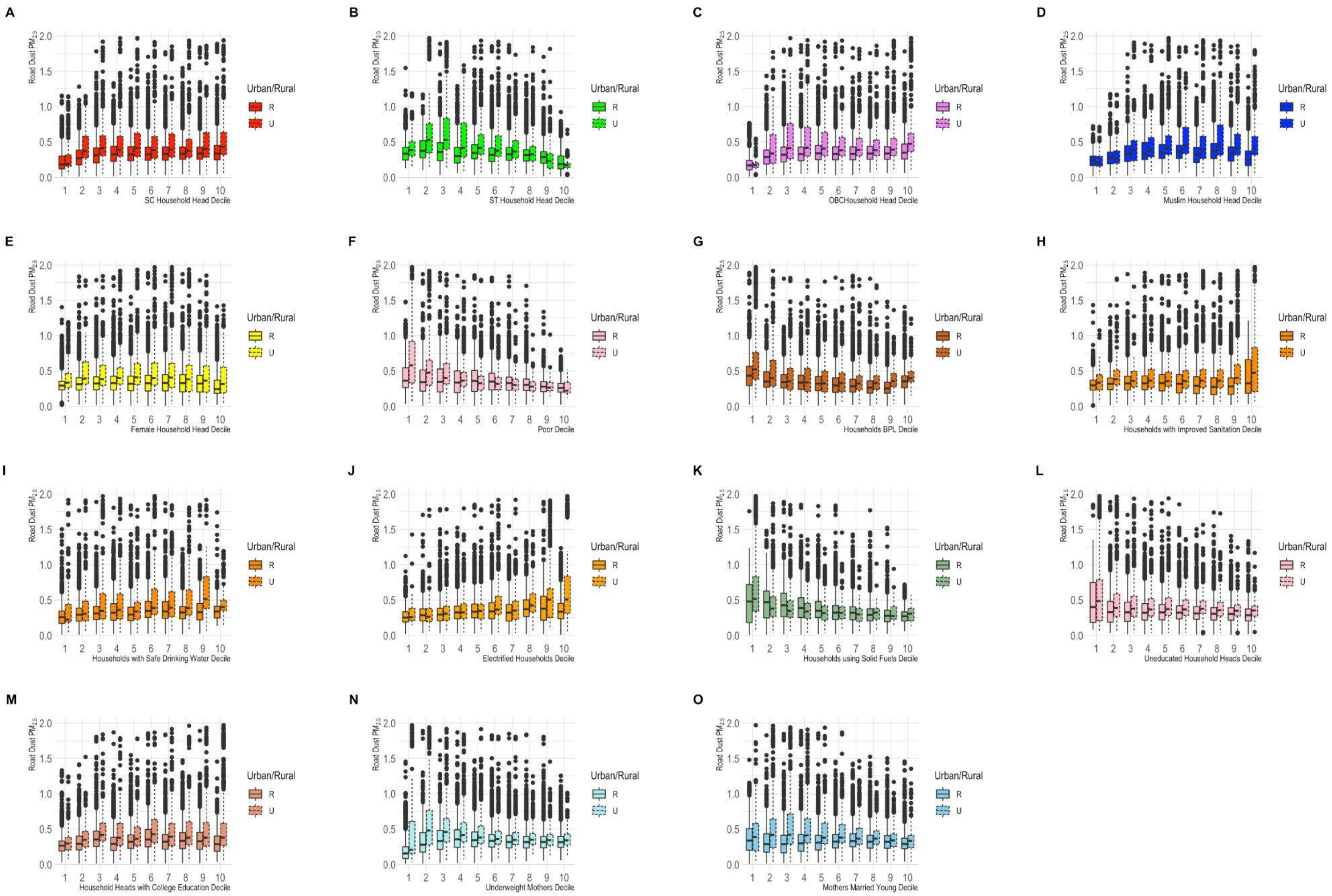

Figure S18*: Road dust-PM$_{2.5}$ concentrations by decile of the different SES prevalence parameters, disaggregated by urban/rural clusters. Road dust-PM$_{2.5}$ concentrations corresponding to the first and tenth decile are highlighted. The boxes correspond to the first and third quartiles of the distribution of road dust-PM$_{2.5}$ concentrations corresponding to each group.*

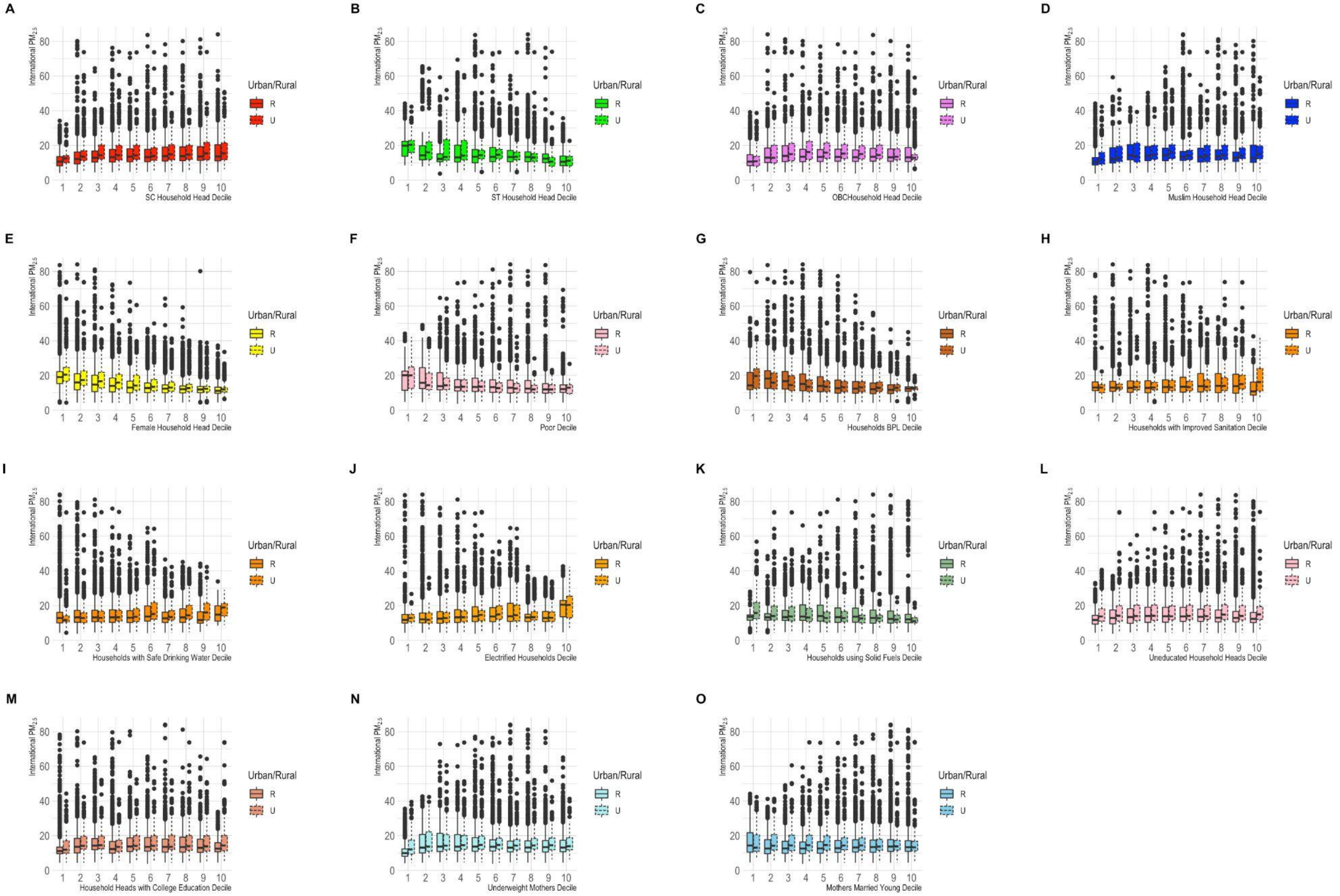

Figure S19*: International-PM$_{2.5}$ concentrations by decile of the different SES prevalence parameters, disaggregated by urban/rural clusters. International-PM$_{2.5}$ concentrations corresponding to the first and tenth decile are highlighted. The boxes correspond to the first and third quartiles of the distribution of international-PM$_{2.5}$ concentrations corresponding to each group.*

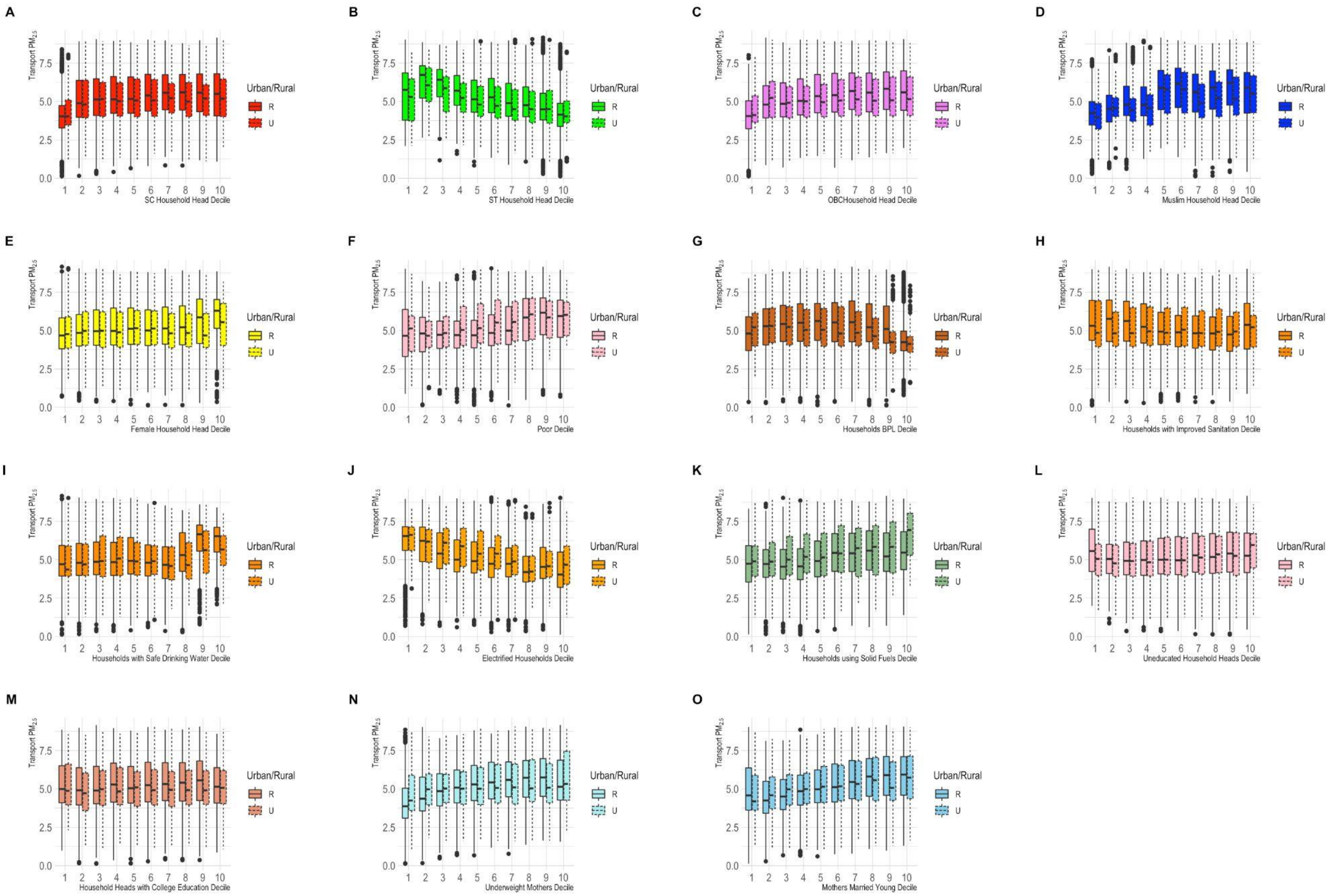

Figure S20*: Transport-$PM_{2.5}$ concentrations by decile of the different SES prevalence parameters, disaggregated by urban/rural clusters. Transport-$PM_{2.5}$ concentrations corresponding to the first and tenth decile are highlighted. The boxes correspond to the first and third quartiles of the distribution of transport-$PM_{2.5}$ concentrations corresponding to each group.*

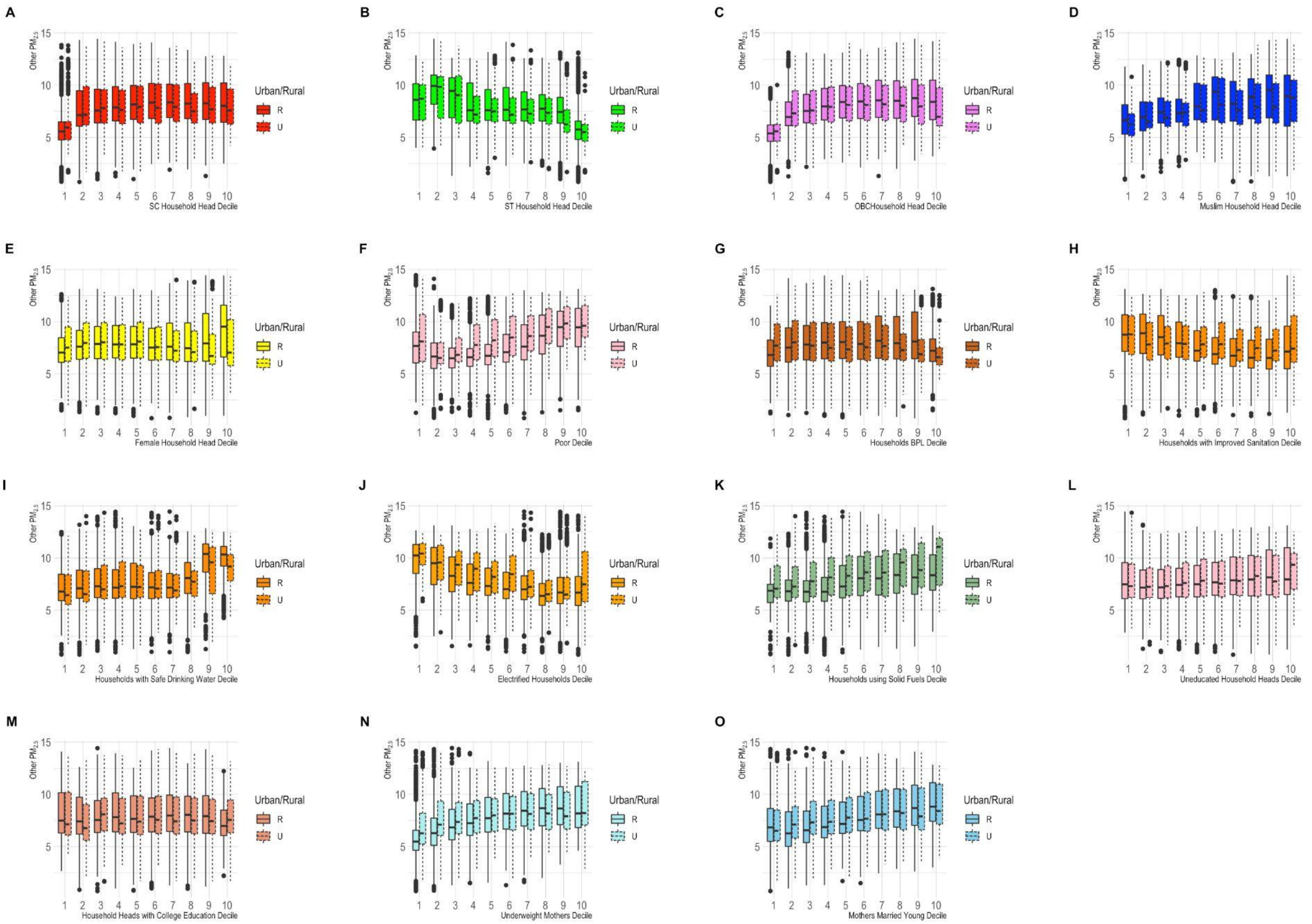

***Figure S21**: Other-$PM_{2.5}$ concentrations by decile of the different SES prevalence parameters, disaggregated by urban/rural clusters. Other-$PM_{2.5}$ concentrations corresponding to the first and tenth decile are highlighted. The boxes correspond to the first and third quartiles of the distribution of other-$PM_{2.5}$ concentrations corresponding to each group.*

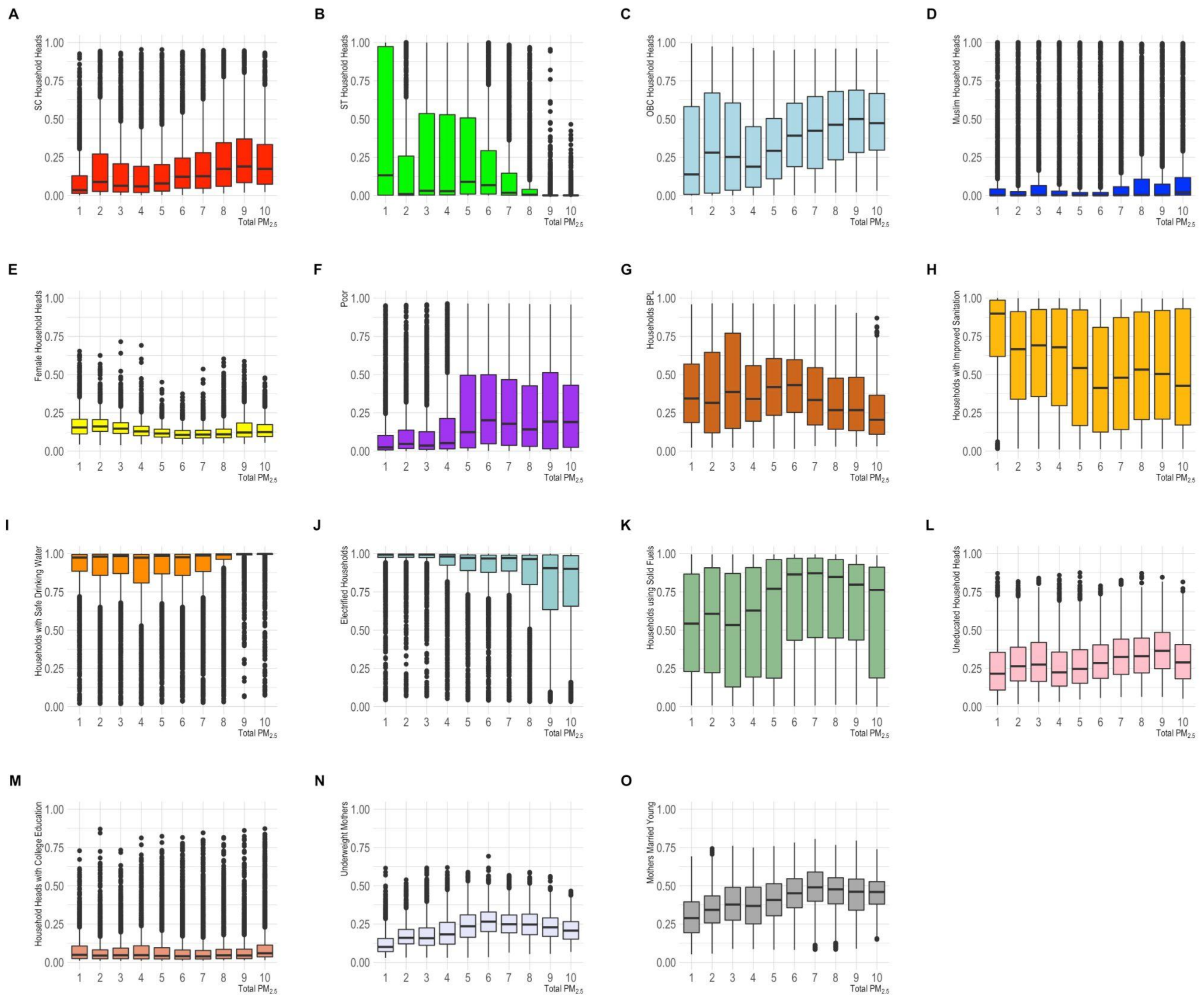

Figure S22: *Prevalence of the various SES parameters considered in this analysis for clusters belonging to different deciles of total-PM$_{2.5}$ concentrations.*

S3: Evaluating Variation in source-specific PM$_{2.5}$ Concentrations across Multiple Geographic Scales

Table S1: *Descriptive Statistics of NFHS-4 cluster level variables analyzed across the 28,072 clusters for which we have total PM$_{2.5}$ data. 8,396 (29.9%) clusters are urban*

	**Mean (St Dev)**	**Min/Max**	**25%**	**Median**	**75%**
**Outcome (units: μg/m^3)**					
Total PM$_{2.5}$	53.4 (25.8)	3.5/131.7	32.6	47.0	69.4
**SES (prevalence is expressed as a fraction)**					

Prevalence of households living in poverty	0.21 (0.25)	0.00/0.97	0.02	0.08	0.35
Prevalence of households with an SC head	0.18 (0.19)	0.00/0.95	0.03	0.10	0.26
Prevalence of households with an ST head	0.19 (0.33)	0.00/1.00	0.00	0.01	0.19
Prevalence of households with an OBC head	0.39 (0.29)	0.00/0.99	0.11	0.39	0.62
Prevalence of households with a Muslim head	0.11 (0.24)	0.00/1.00	0.00	0.00	0.06
Prevalence of households with a female head	0.14 (0.07)	0.04/0.71	0.10	0.13	0.17
Prevalence of households with a college-educated head	0.08 (0.11)	0.01/0.87	0.02	0.04	0.09
Prevalence of households with a head with no formal education	0.30 (0.16)	0.01/0.88	0.17	0.28	0.41
Prevalence of households with electricity	0.89 (0.20)	0.03/1.00	0.88	0.98	0.99
Prevalence of households using solid cooking fuels	0.61 (0.35)	0.00/1.00	0.27	0.74	0.94
Prevalence of households with improved sanitation	0.57 (0.35)	0.01/1.00	0.23	0.61	0.93
Prevalence of households with access to safe drinking water	0.90 (0.20)	0.02/1.00	0.92	0.99	1.00
Prevalence of households with a BPL ration card	0.38 (0.25)	0.02/0.97	0.16	0.33	0.55
Prevalence of mothers married young	0.41 (0.15)	0.05/0.81	0.30	0.41	0.52
Prevalence of underweight mothers	0.21 (0.10)	0.03/0.69	0.14	0.21	0.28

***Table S2**: Descriptive Statistics of NFHS-4 cluster level variables analyzed across the 27,534 clusters included in this analysis. 8,199 (29.8%) clusters are urban*

	**Mean (St Dev)**	**Min/Max**	**25%**	**Median**	**75%**
**Outcome (units: μg/m^3)**					
Anthropogenic $PM_{2.5}$	65.6 (17.8)	5.4/100.1	53.1	62.6	82.4

Source-specific $PM_{2.5}$					
Agricultural Residue Burning	5.7 (5.1)	0.1/42.2	2.8	4.5	6.7
Domestic Burning	19.5 (9.9)	0.7/51.7	12.2	16.0	24.3
Power	4.4 (2.1)	0.1/11.2	2.7	4.3	5.5
Road Dust	0.4 (0.2)	0.0/2.0	0.2	0.3	0.5
International	15.3 (7.3)	3.7/84.0	10.7	13.4	18.3
Industrial	11.5 (5.1)	0.3/48.6	8.5	10.4	13.6
Other	8.0 (2.3)	0.7/14.5	6.3	7.6	9.7
Transport	5.2 (1.6)	0.1/9.2	4.1	5.1	6.5
**SES (prevalence is expressed as a fraction)**					
Prevalence of households living in poverty	0.21 (0.26)	0.00/0.97	0.02	0.09	0.35
Prevalence of households with an SC head	0.18 (0.19)	0.00/0.95	0.03	0.11	0.27
Prevalence of households with an ST head	0.19 (0.33)	0.00/1.00	0.00	0.01	0.19
Prevalence of households with an OBC head	0.39 (0.29)	0.00/0.99	0.11	0.37	0.62
Prevalence of households with a Muslim head	0.11 (0.24)	0.00/1.00	0.00	0.00	0.06
Prevalence of households with a female head	0.14 (0.07)	0.04/0.71	0.10	0.12	0.17
Prevalence of households with a college-educated head	0.08 (0.11)	0.01/0.87	0.02	0.04	0.09
Prevalence of households with a head with no formal education	0.30 (0.16)	0.01/0.88	0.17	0.28	0.41
Prevalence of households with electricity	0.89 (0.20)	0.03/1.00	0.88	0.98	0.99
Prevalence of households using solid cooking fuels	0.61 (0.35)	0.00/1.00	0.27	0.74	0.94
Prevalence of households with improved sanitation	0.57 (0.35)	0.01/1.00	0.23	0.61	0.92
Prevalence of households with access to safe drinking water	0.90 (0.20)	0.02/1.00	0.92	0.99	1.00

Prevalence of households with a BPL ration card	0.38 (0.25)	0.02/0.97	0.16	0.33	0.56
Prevalence of mothers married young	0.41 (0.15)	0.05/0.81	0.30	0.42	0.52
Prevalence of underweight mothers	0.21 (0.10)	0.03/0.69	0.14	0.21	0.28

***Table S3**: % of Variance in total $PM_{2.5}$ levels attributable to the cluster, district and state-levels*

	Variance (%) in models that only adjust for a single SES parameter		
	**State (n = 36)**	**District (n = 640)**	**Cluster (n = 28,801)**
**Only adjusting for urban/rural**	520.2 (83.5%)	89.0 (14.3%)	13.7 (2.2%)
**Also adjusting for log(population density)**	477.8 (83.2%)	83.2 (14.5%)	13.0 (2.3%)
	**Variance (% explained in variation by adjusting for SES) in models that adjust for a single SES parameter, in addition to log(population density)**		
**SC Household Heads**	476.6 (0.3%)	83.1 (0.1%)	13.0 (0%)
**ST Household Heads**	471.2 (1.4%)	82.2 (1.2%)	12.9 (0.8%)
**OBC Household Heads**	477.0 (0.2%)	83.0 (0.2%)	13.0 (0%)
**Muslim Household Heads**	477.5 (0.1%)	83.2 (0%)	13.0 (0%)
**Female Household Heads**	477.7 (0%)	83.2 (0%)	13.0 (0%)
**Poor**	481.4 (0.8%)	82.9 (0.4%)	13.0 (0%)
**Households BPL with Ration Cards**	477.6 (0%)	83.1 (0.1%)	13.0 (0%)
**Households with Improved Sanitation**	478.7 (0.2%)	83.3 (0.1%)	13.0 (0%)
**Households with Safe Drinking Water**	477.6 (0%)	83.2 (0%)	13.0 (0%)
**Electrified Households**	478.4 (0.1%)	83.2 (0%)	13.0 (0%)
**Households using Solid Fuels**	478.6 (0.2%)	83.3 (0.1%)	13.0 (0%)
**Uneducated Household heads**	479.1 (0.3%)	83.1 (0.1%)	13.0 (0%)
**Household Heads with College Education**	477.8 (0%)	83.2 (0%)	13.0 (0%)
**Mothers Married Young**	476.8 (0.2%)	83.1 (0.1%)	13.0 (0%)
**Underweight Mothers**	479.4 (0.3%)	83.2 (0%)	13.0 (0%)
**Fully-adjusted Models**	472.6 (1.1%)	81.6 (1.9%)	12.9 (0.8%)

***Table S4**: % of Variance in anthropogenic $PM_{2.5}$ levels attributable to the cluster, district and state-levels*

	Variance (%) in models that only adjust for a single SES parameter		
	**State (n = 36)**	**District (n = 640)**	**Cluster (n = 28,801)**
**Only adjusting for urban/rural**	255.5 (82.8%)	44.5 (14.4%)	8.4 (2.7%)
**Also adjusting for log(population density)**	235.9 (82.6%)	41.5 (14.5%)	8.3 (2.9%)
	**Variance (% explained in variation by adjusting for SES) in models that adjust for a single SES parameter, in addition to log(population density)**		
**SC Household Heads**	235.6 (0.1%)	41.5 (0%)	8.2 (1.2%)
**ST Household Heads**	232.4 (1.5%)	41.0 (1.2%)	8.2 (1.2%)
**OBC Household Heads**	235.3 (0.3%)	41.5 (0%)	8.2 (1.2%)
**Muslim Household Heads**	235.9 (0%)	41.5 (0%)	8.3 (0%)
**Female Household Heads**	235.7 (0.1%)	41.5 (0%)	8.3 (0%)
**Poor**	236.9 (0.4%)	41.5 (0%)	8.2 (1.2%)
**Households BPL with Ration Cards**	236.0 (0%)	41.5 (0%)	8.3 (0%)
**Households with Improved Sanitation**	235.7 90.1%)	41.5 (0%)	8.3 (0%)
**Households with Safe Drinking Water**	235.9 (0%)	41.5 (0%)	8.3 (0%)
**Electrified Households**	236.5 (0.3%)	41.5 (0%)	8.2 (1.2%)
**Households using Solid Fuels**	236.1 (0.1%)	41.6 (0.2%)	8.3 (0%)
**Uneducated Household heads**	236.1 (0.1%)	41.5 (0%)	8.3 (0%)
**Household Heads with College Education**	235.9 (0%)	41.5 (0%)	8.3 (0%)
**Mothers Married Young**	235.2 (0.3%)	41.5 (0%)	8.3 (0%)
**Underweight Mothers**	236.5 (0.3%)	41.5 (0%)	8.3 (0%)
**Fully-adjusted Models**	231.7 (1.8%)	41.0 (1.2%)	8.2 (1.2%)

***Table S5**: % of Variance in source-specific $PM_{2.5}$ levels attributable to the cluster, district and state-levels*

**Industrial-$PM_{2.5}$**			
	**Variance (%) in models that only adjust for a single SES parameter**		
	**State (n = 36)**	**District (n = 640)**	**Cluster (n = 28,801)**

**Only adjusting for urban/rural**	22.0 (69.6%)	8.2 (25.9%)	1.4 (4.4%)
**Also adjusting for log(population density)**	21.5 (69.4%)	8.1 (26.1%)	1.4 (4.5%)
	**Variance (% explained in variation by adjusting for SES) in models that adjust for a single SES parameter, in addition to log(population density)**		
**Fully-adjusted Models**	21.4 (0.5%)	8.1 (0%)	1.4 (0%)
**Power-$PM_{2.5}$**			
	**Variance (%) in models that only adjust for a single SES parameter**		
	**State (n = 36)**	**District (n = 640)**	**Cluster (n = 28,801)**
**Only adjusting for urban/rural**	3.9 (81.3%)	0.8 (16.7%)	0.1 (2.1%)
**Also adjusting for log(population density)**	3.8 (80.9%)	0.8 (17.0%)	0.1 (2.1%)
	**Variance (% explained in variation by adjusting for SES) in models that adjust for a single SES parameter, in addition to log(population density)**		
**Fully-adjusted Models**	3.8 (0%)	0.8 (0%)	0.1 (0%)
**Domestic Burning-$PM_{2.5}$**			
	**Variance (%) in models that only adjust for a single SES parameter**		
	**State (n = 36)**	**District (n = 640)**	**Cluster (n = 28,801)**
**Only adjusting for urban/rural**	52.9 (76.3%)	14.3 (20.6%)	2.1 (3.0%)
**Also adjusting for log(population density)**	50.8 (76.7%)	13.4 (20.2%)	2.0 (3.0%)
	**Variance (% explained in variation by adjusting for SES) in models that adjust for a single SES parameter, in addition to log(population density)**		
**Fully-adjusted Models**	50.0 (1.6%)	13.2 (1.5%)	2.0 (0%)
**Agricultural Residue Burning-$PM_{2.5}$**			
	**Variance (%) in models that only adjust for a single SES parameter**		
	**State (n = 36)**	**District (n = 640)**	**Cluster (n = 28,801)**
**Only adjusting for urban/rural**	21.4 (70.4%)	8.0 (26.3%)	1.0 (3.3%)
**Also adjusting for log(population density)**	21.2 (70.2%)	8.0 (26.5%)	1.0 (3.3%)
	**Variance (% explained in variation by adjusting for SES) in models that adjust for a single SES parameter, in addition to log(population density)**		
**Fully-adjusted Models**	21.0 (0.9%)	7.9 (1.3%)	1.0 (0%)
**Transport-$PM_{2.5}$**			
	**Variance (%) in models that only adjust for a single SES parameter**		
	**State (n = 36)**	**District (n = 640)**	**Cluster (n = 28,801)**

**Only adjusting for urban/rural**	1.4 (58.3%)	0.8 (33.3%)	0.2 (8.3%)
**Also adjusting for log(population density)**	1.3 (56.5%)	0.8 (34.8%)	0.2 (8.7%)
	**Variance (% explained in variation by adjusting for SES) in models that adjust for a single SES parameter, in addition to log(population density)**		
**Fully-adjusted Models**	1.3 (0%)	0.8 (0%)	0.2 (0%)
**International-$PM_{2.5}$**			
	**Variance (%) in models that only adjust for a single SES parameter**		
	**State (n = 36)**	**District (n = 640)**	**Cluster (n = 28,801)**
**Only adjusting for urban/rural**	33.0 (65.7%)	15.6 (31.1%)	1.6 (3.2%)
**Also adjusting for log(population density)**	33.3 (66.1%)	15.5 (30.8%)	1.6 (3.2%)
	**Variance (% explained in variation by adjusting for SES) in models that adjust for a single SES parameter, in addition to log(population density)**		
**Fully-adjusted Models**	32.9 (1.2%)	15.5 (0%)	1.6 (0%)
**Road Dust-$PM_{2.5}$**			
	**Variance (%) in models that only adjust for a single SES parameter**		
	**State (n = 36)**	**District (n = 640)**	**Cluster (n = 28,801)**
**Only adjusting for urban/rural**	0.054 (68.4%)	0.019 (24.1%)	0.006 (7.6%)
**Also adjusting for log(population density)**	0.046 (67.6%)	0.016 (23.5%)	0.006 (8.8%)
	**Variance (% explained in variation by adjusting for SES) in models that adjust for a single SES parameter, in addition to log(population density)**		
**Fully-adjusted Models**	0.046 (0%)	0.016 (0%)	0.006 (0%)
**Other-$PM_{2.5}$**			
	**Variance (%) in models that only adjust for a single SES parameter**		
	**State (n = 36)**	**District (n = 640)**	**Cluster (n = 28,801)**
**Only adjusting for urban/rural**	3.7 (74.0%)	1.1 (22.0%)	0.2 (4%)
**Also adjusting for log(population density)**	3.4 (73.9%)	1.0 (21.7%)	0.2 (4.3%)
	**Variance (% explained in variation by adjusting for SES) in models that adjust for a single SES parameter, in addition to log(population density)**		
**Fully-adjusted Models**	3.3 (2.9%)	1.0 (0%)	0.2 (0%)

S4: Evaluating Associations between source-specific $PM_{2.5}$ levels and different EJ dimensions

***Table S6**: Associations between the different SES parameters and anthropogenic $PM_{2.5}$ in models only adjusted for urban/rural and the logarithm of population density, as well in fully-adjusted models, mutually adjusted for other SES parameters. We also display associations from fully-adjusted models disaggregated by urban/rural*

	**Anthropogenic-$PM_{2.5}$**			
	**All**		**Urban**	**Rural**
	**Unadjusted**	**Fully-Adjusted**	**Fully-Adjusted**	**Fully-Adjusted**
**SC Household Heads**	0.038<br>(-0.002, 0.078)	0.015<br>(-0.038, 0.067)	-0.054<br>(-0.130, 0.022)	0.028<br>(-0.040, 0.095)
**ST Household Heads**	-0.315*<br>(-0.385, -0.245)	-0.292*<br>(-0.383, -0.200)	-0.173*<br>(-0.329, -0.016)	-0.215*<br>(-0.331, -0.100)
**OBC Household Heads**	0.111*<br>(0.062, 0.161)	0.038<br>(-0.029, 0.106)	-0.020<br>(-0.122, 0.082)	0.050<br>(-0.035, 0.135)
**Muslim Household Heads**	0.023<br>(-0.020, 0.066)	0.010<br>(-0.037, 0.056)	-0.015<br>(-0.084, 0.054)	0.017<br>(-0.044, 0.079)
**Female Household Heads**	-0.046<br>(-0.115, 0.022)	-0.052<br>(-0.123, 0.018)	-0.129*<br>(-0.233, -0.024)	-0.007<br>(-0.095, 0.081)
**Poor**	-0.092*<br>(-0.153, -0.031)	-0.008<br>(-0.101, 0.086)	0.051<br>(-0.059, 0.162)	0.138*<br>(0.023, 0.253)
**Households BPL with Ration Cards**	-0.058<br>(-0.125, 0.009)	-0.036<br>(-0.109, 0.036)	-0.017<br>(-0.129, 0.096)	-0.008<br>(-0.093, 0.078)
**Households with Improved Sanitation**	-0.029<br>(-0.089, 0.032)	-0.137*<br>(-0.219, -0.055)	0.089<br>(-0.001, 0.180)	-0.108*<br>(-0.205, -0.012)
**Households with Safe Drinking Water**	0.006<br>(-0.039, 0.051)	-0.013<br>(-0.059, 0.032)	-0.042<br>(-0.112, 0.029)	0.000<br>(-0.056, 0.057)
**Electrified Households**	0.080*<br>(0.028, 0.132)	0.072*<br>(0.008, 0.137)	0.083<br>(-0.007, 0.174)	0.060<br>(-0.019, 0.138)
**Households using Solid Fuels**	-0.037<br>(-0.104, 0.031)	-0.067<br>(-0.154, 0.019)	0.106*<br>(0.002, 0.210)	-0.085*<br>(-0.163, -0.008)
**Uneducated Household heads**	-0.032<br>(-0.085, 0.022)	0.002<br>(-0.064, 0.068)	0.035<br>(-0.062, 0.131)	-0.083*<br>(-0.160, -0.006)
**Household Heads with College Education**	-0.034<br>(-0.075, 0.008)	-0.035<br>(-0.084, 0.014)	-0.030<br>(-0.104, 0.045)	-0.013<br>(-0.066, 0.039)
**Mothers Married Young**	0.072<br>(-0.010, 0.155)	0.051<br>(-0.037, 0.138)	0.053<br>(-0.078, 0.183)	0.019<br>(-0.086, 0.125)
**Underweight Mothers**	-0.040<br>(-0.108, 0.027)	-0.035<br>(-0.106, 0.035)	0.015<br>(-0.090, 0.120)	-0.054<br>(-0.138, 0.030)

Domestic burning-$PM_{2.5}$ was significantly higher in clusters with a high prevalence of Muslim household heads: 0.028 μg/m^3 (95% CI: 0.005 μg/m^3, 0.052 μg/m^3), poor households: 0.049 μg/m^3 (95% CI: 0.002 μg/m^3, 0.095 μg/m^3), and electrified households: 0.049 μg/m^3 (95% CI: 0.017 μg/m^3, 0.081 μg/m^3). Domestic burning was significantly lower in clusters with a higher prevalence of ST households -0.169 μg/m^3 (95% CI: -0.214 μg/m^3, -0.132 μg/m^3), households with improved sanitation: -0.077 μg/m^3 (95% CI: -0.118 μg/m^3, -0.037 μg/m^3), and household heads with no formal education: -0.081 μg/m^3 (95% CI: -0.114 μg/m^3, -0.048 μg/m^3).

Industrial-$PM_{2.5}$ levels were significantly lower in clusters with a high prevalence of households heads with no formal education: -0.024 μg/m^3 (-0.044 μg/m^3, -0.003 μg/m^3), households using solid fuels: -0.067 μg/m^3(95% CI: -0.103 μg/m^3, -0.031 μg/m^3). Power-$PM_{2.5}$ was significantly higher in clusters with a higher prevalence of electrified households: 0.008 μg/m^3 (95% CI: 0.00 μg/m^3, 0.016 μg/m^3), and a lower prevalence of Muslim householdheads: -0.006 μg/m^3 (95% CI: -0.012 μg/m^3, -0.001 μg/m^3).

International-$PM_{2.5}$ was significantly higher in clusters with a higher prevalence of Muslim household heads: 0.027 μg/m^3 (95% CI: 0.006 μg/m^3, 0.048 μg/m^3), household heads with no formal education: 0.043 μg/m^3(95% CI: 0.013 μg/m^3, 0.072 μg/m^3), college-educated household heads: 0.027 μg/m^3 (95% CI: 0.006 μg/m^3, 0.049 μg/m^3), and lower in clusters with a higher prevalence of ST households: -0.042 μg/m^3(95% CI: -0.083 μg/m^3, -0.002 μg/m^3), households with improved sanitation: -0.042 μg/m^3 (95% CI: -0.078 μg/m^3, -0.005 μg/m^3), and households with safe drinking water: -0.022 μg/m^3 (95% CI: -0.043 μg/m^3, -0.002 μg/m^3)

Agricultural residue burning-$PM_{2.5}$ was significantly higher in clusters with a higher prevalence of household heads with no formal education: 0.050 μg/m^3 (95% CI: 0.027 μg/m^3, 0.073 μg/m^3), households using solid-fuels: 0.038 μg/m^3 (95% CI: 0.008 μg/m^3, 0.068 μg/m^3), and lower in clusters with a higher prevalence of ST households: -0.041 μg/m^3 (95% CI: -0.073 μg/m^3, -0.010 μg/m^3), Muslim households: -0.033 μg/m^3 (95% CI: -0.049 μg/m^3, -0.017 μg/m^3), households headed by a woman: -0.035 μg/m^3(95% CI: -0.060 μg/m^3, -0.011 μg/m^3).

Road dust-$PM_{2.5}$ was significantly lower in clusters with a higher prevalence of households with safe drinking water: -0.002 μg/m^3 (95% CI: -0.003 μg/m^3, -0.001 μg/m^3). Transport-$PM_{2.5}$ was significantly lower in clusters with a higher prevalence of households headed by women: 0.013 μg/m^3 (95% CI: 0.002 μg/m^3, 0.025 μg/m^3), electrified households: 0.026 μg/m^3 (95% CI; 0.016 μg/m^3, 0.037 μg/m^3), and lower in clusters with a higher prevalence of ST households: -0.019 μg/m^3 (95% CI: -0.034 μg/m^3, -0.005 μg/m^3), households with improved sanitation: -0.015 μg/m^3 (95% CI: -0.029 μg/m^3, -0.002 μg/m^3), household heads with no formal education: -0.018 μg/m^3 (95% CI: -0.030 μg/m^3, -0.009 μg/m^3).

Table S7*: Associations between the different SES parameters and source-specific $PM_{2.5}$ in models only adjusted for urban/rural and the logarithm of population density, as well in fully-adjusted models, mutually adjusted for other SES parameters.*

	**Domestic Burning**	**Industrial**	**Power**	**International**	**Agricultural Residue**	**Road Dust**	**Transport**	**Other**

					Burning			
	Unadjusted	Unadjusted	Unadjusted	Unadjusted	Unadjusted	Unadjusted	Unadjusted	Unadjusted
**SC Household Heads**	0.011 (-0.009, 0.031)	-0.002 (-0.018, 0.015)	-0.004 (-0.008, 0.001)	0.008 (-0.009, 0.026)	0.022* (0.008, 0.036)	-0.000 (-0.001, 0.001)	0.004 (-0.002, 0.011)	0.003 (-0.004, 0.010)
**ST Household Heads**	-0.145* (-0.180, -0.110)	-0.042* (-0.071, -0.013)	0.009* (0.001, 0.017)	-0.056* (-0.087, -0.025)	-0.040* (-0.064, -0.016)	-0.001 (-0.003, 0.001)	-0.019* (-0.030, -0.008)	-0.046* (-0.058, -0.035)
**OBC Household Heads**	0.036* (0.011, 0.061)	0.015 (-0.005, 0.036)	-0.003 (-0.008, 0.003)	0.031* (0.009, 0.053)	0.003 (-0.014, 0.021)	0.000 (-0.001, 0.002)	-0.000 (-0.008, 0.007)	0.017* (0.009, 0.026)
**Muslim Household Heads**	0.023* (0.002, 0.045)	0.003 (-0.015, 0.021)	-0.006* (-0.012, -0.001)	0.028* (0.009, 0.047)	-0.020* (-0.035, -0.005)	-0.001* (-0.002, -0.000)	-0.005 (-0.012, 0.002)	0.002 (-0.005, 0.009)
**Female Household Heads**	0.039* (0.005, 0.073)	-0.024 (-0.052, 0.004)	-0.000 (-0.008, 0.008)	-0.041* (-0.071, -0.010)	-0.022 (-0.046, 0.001)	-0.002 (-0.003, 0.000)	0.008 (-0.003, 0.019)	-0.002 (-0.013, 0.010)
**Poor**	-0.016 (-0.046, 0.014)	-0.033* (-0.058, -0.008)	0.007 (-0.000, 0.014)	0.001 (-0.026, 0.028)	-0.023* (-0.044, -0.001)	-0.001 (-0.003, 0.000)	-0.018* (-0.028, -0.008)	-0.008 (-0.018, 0.002)
**Households BPL with Ration Cards**	-0.002 (-0.036, 0.031)	-0.015 (-0.043, 0.012)	0.004 (-0.004, 0.011)	-0.002 (-0.032, 0.027)	0.001 (-0.022, 0.024)	-0.002 (-0.003, 0.000)	-0.003 (-0.014, 0.008)	-0.005 (-0.016, 0.006)
**Households with Improved Sanitation**	-0.037* (-0.067, -0.007)	0.010 (-0.015, 0.035)	-0.006 (-0.013, 0.001)	-0.029* (-0.056, -0.002)	0.007 (-0.014, 0.028)	0.003* (0.001, 0.005)	-0.002 (-0.011, 0.008)	-0.007 (-0.017, 0.003)
**Households with Safe Drinking Water**	0.023* (0.001, 0.045)	-0.001 (-0.019, 0.018)	-0.006* (-0.012, -0.001)	-0.022* (-0.042, -0.002)	0.007 (-0.009, 0.022)	-0.001* (-0.003, -0.000)	-0.003 (-0.010, 0.004)	-0.003 (-0.011, 0.004)
**Electrified Households**	0.035* (0.009, 0.061)	0.031 (0.009, 0.052)	0.002 (-0.004, 0.008)	-0.015 (-0.038, 0.008)	0.013 (-0.005, 0.031)	-0.001 (-0.002, 0.001)	0.026* (0.017, 0.034)	0.013* (0.005, 0.022)
**Households using Solid Fuels**	0.016 (-0.017, 0.050)	-0.050* (-0.078, -0.023)	-0.005 (-0.013, 0.003)	0.027 (-0.002, 0.057)	0.029* (0.006, 0.053)	-0.004* (-0.005, -0.002)	-0.000 (-0.011, 0.011)	-0.011* (-0.022, -0.000)
**Uneducated Household heads**	-0.044* (-0.071, -0.018)	-0.017 (-0.039, 0.005)	0.000 (-0.006, 0.006)	0.031* (0.008, 0.055)	0.026* (0.007, 0.044)	-0.003* (-0.004, -0.001)	-0.018* (-0.027, -0.010)	-0.014* (-0.023, -0.006)
**Household Heads with College Education**	-0.011 (-0.032, 0.010)	-0.007 (-0.025, 0.010)	0.004 (-0.000, 0.009)	0.003 (-0.015, 0.022)	-0.017* (-0.031, -0.003)	0.002* (0.000, 0.003)	0.006 (-0.001, 0.013)	0.000 (-0.007, 0.007)
**Mothers Married Young**	0.039 (-0.002, 0.080)	-0.017 (-0.051, 0.017)	0.006 (-0.003, 0.016)	0.024 (-0.012, 0.061)	0.008 (-0.021, 0.036)	-0.004* (-0.006, -0.002)	-0.006 (-0.019, 0.007)	0.007 (-0.006, 0.021)
**Underweight Mothers**	0.019 (-0.015, 0.053)	-0.006 (-0.034, 0.022)	0.005 (-0.003, 0.013)	-0.013 (-0.043, 0.017)	0.003 (-0.020, 0.027)	-0.002* (-0.004, -0.001)	0.002 (-0.009, 0.013)	-0.008 (-0.020, 0.003)

***Table S8**: Associations between the different SES parameters and source-specific $PM_{2.5}$ in models only adjusted for urban/rural and the logarithm of population density, as well in fully-adjusted models, mutually adjusted for other SES parameters.*

	Domestic Burning	Industrial	Power	International	Agricultural Residue Burning	Road Dust	Transport	Other
	Fully-Adjusted	Fully-Adjusted	Fully-Adjusted	Fully-Adjusted	Fully-Adjusted	Fully-Adjusted	Fully-Adjusted	Fully-Adjusted

**SC Household Heads**	-0.015 (-0.041, 0.011)	-0.005 (-0.027, 0.017)	-0.005 (-0.012, 0.001)	0.017 (-0.006, 0.040)	0.008 (-0.010, 0.026)	0.001 (-0.001, 0.002)	0.000 (-0.008, 0.009)	0.001 (-0.008, 0.009)
**ST Household Heads**	-0.169* (-0.214, -0.132)	-0.036 (-0.074, 0.002)	0.001 (-0.009, 0.012)	-0.042* (-0.083, -0.002)	-0.041* (-0.073, -0.010)	0.000 (-0.002, 0.003)	-0.019* (-0.034, -0.005)	-0.046* (-0.061, -0.031)
**OBC Household Heads**	-0.019 (-0.052, 0.015)	0.001 (-0.027, 0.029)	-0.002 (-0.010, 0.006)	0.029 (-0.001, 0.058)	-0.003 (-0.026, 0.020)	0.001 (-0.001, 0.003)	-0.005 (-0.016, 0.005)	0.007 (-0.004, 0.018)
**Muslim Household Heads**	0.028* (0.005, 0.052)	-0.001 (-0.020, 0.018)	-0.006* (-0.012, -0.001)	0.027* (0.006, 0.048)	-0.033* (-0.049, -0.017)	-0.001 (-0.002, 0.000)	-0.002 (-0.009, 0.006)	0.003 (-0.005, 0.010)
**Female Household Heads**	0.056* (0.021, 0.091)	-0.029 (-0.058, 0.000)	0.000 (-0.008, 0.008)	-0.048 (-0.079, -0.016)	-0.035* (-0.060, -0.011)	-0.001 (-0.003, 0.000)	0.013* (0.002, 0.025)	0.001 (-0.010, 0.013)
**Poor**	0.049* (0.002, 0.095)	-0.006 (-0.045, 0.033)	0.010 (-0.001, 0.021)	-0.039 (-0.080, 0.003)	-0.031 (-0.063, 0.002)	0.000 (-0.002, 0.003)	-0.000 (-0.015, 0.015)	0.015 (-0.000, 0.031)
**Households BPL with Ration Cards**	0.009 (-0.027, 0.045)	-0.004 (-0.034, 0.026)	0.005 (-0.003, 0.014)	-0.005 (-0.037, 0.027)	-0.008 (-0.033, 0.017)	-0.000 (-0.002, 0.002)	0.000 (-0.011, 0.012)	0.002 (-0.010, 0.014)
**Households with Improved Sanitation**	-0.077* (-0.118, -0.037)	-0.032 (-0.066, 0.002)	-0.005 (-0.015, 0.005)	-0.042* (-0.078, -0.005)	0.026 (-0.003, 0.054)	0.002 (-0.000, 0.004)	-0.015* (-0.029, -0.002)	-0.026* (-0.039, -0.012)
**Households with Safe Drinking Water**	0.018 (-0.005, 0.041)	-0.006 (-0.025, 0.013)	-0.005 (-0.011, 0.000)	-0.022* (-0.043, -0.002)	0.004 (-0.011, 0.020)	-0.002* (-0.003, -0.001)	-0.006 (-0.013, 0.001)	-0.006 (-0.014, 0.002)
**Electrified Households**	0.049* (0.017, 0.081)	0.024 (-0.003, 0.051)	0.008* (0.000, 0.016)	-0.020 (-0.049, 0.008)	0.007 (-0.016, 0.029)	-0.002 (-0.003, 0.000)	0.026* (0.016, 0.037)	0.017* (0.007, 0.028)
**Households using Solid Fuels**	0.018 (-0.025, 0.061)	-0.067* (-0.103, -0.031)	-0.013* (-0.023, -0.003)	0.011 (-0.027, 0.049)	0.038* (0.008, 0.068)	-0.002 (-0.004, 0.000)	0.012 (-0.002, 0.026)	-0.017* (-0.031, -0.003)
**Uneducated Household heads**	-0.081* (-0.114, -0.048)	0.002 (-0.025, 0.030)	0.001 (-0.007, 0.009)	0.043* (0.013, 0.072)	0.050* (0.027, 0.073)	-0.001 (-0.003, 0.001)	-0.018* (-0.030, -0.009)	-0.015* (-0.026, -0.004)
**Household Heads with College Education**	-0.008 (-0.032, 0.017)	-0.024* (-0.044, -0.003)	0.004 (-0.002, 0.010)	0.027* (0.006, 0.049)	-0.013 (-0.030, 0.004)	0.000 (-0.001, 0.001)	0.004 (-0.004, 0.012)	-0.002 (-0.010, 0.006)
**Mothers Married Young**	0.030 (-0.014, 0.073)	-0.016 (-0.052, 0.020)	0.010 (-0.000, 0.020)	0.011 (-0.028, 0.050)	-0.008 (-0.038, 0.023)	-0.003 (-0.005, -0.000)	-0.004 (-0.018, 0.010)	0.008 (-0.007, 0.022)
**Underweight Mothers**	0.018 (-0.017, 0.053)	0.006 (-0.024, 0.035)	0.005 (-0.003, 0.014)	-0.024 (-0.055, 0.008)	-0.000 (-0.025, 0.024)	-0.001 (-0.003, 0.001)	0.006 (-0.006, 0.017)	-0.007 (-0.019, 0.005)

***Table S9**: Associations between the different SES parameters and source-specific $PM_{2.5}$ in models in fully-adjusted models, mutually adjusted for other SES parameters, disaggregated by urban/rural*

	**Domestic Burning**		**Industrial**		**Power**		**International**		**Agricultural Residue Burning**		**Road Dust**		**Transport**		**Other**	
	**Urban**	**Rural**	**Urban**	**Rural**	**Urban**	**Rural**	**Urban**	**Rural**	**Urban**	**Rural**	**Urban**	**Rural**	**Urban**	**Rural**	**Urban**	**Rural**
**SC Household Heads**	-0.019 (-0.054, 0.015)	-0.025 (-0.059, 0.010)	-0.005 (-0.035, 0.024)	-0.004 (-0.033, 0.025)	-0.006 (-0.015, 0.003)	-0.002 (-0.010, 0.007)	-0.007 (-0.036, 0.022)	0.034* (0.002, 0.065)	-0.023 (-0.052, 0.005)	0.018 (-0.005, 0.040)	0.001 (-0.001, 0.004)	0.001 (-0.001, 0.002)	-0.001 (-0.013, 0.012)	-0.003 (-0.014, 0.007)	-0.002 (-0.014, 0.011)	0.001 (-0.010, 0.012)
**ST Household Heads**	-0.081* (-0.152, -0.010)	-0.194* (-0.253, -0.135)	-0.001 (-0.062, 0.060)	0.012 (-0.037, 0.061)	0.000 (-0.018, 0.018)	0.009 (-0.005, 0.023)	-0.011 (-0.072, 0.049)	-0.024 (-0.078, 0.029)	-0.030 (-0.089, 0.029)	-0.039* (-0.078, -0.000)	0.001 (-0.004, 0.006)	-0.001 (-0.003, 0.002)	-0.026* (-0.028, 0.005)	-0.023* (-0.042, -0.005)	-0.051* (-0.077, -0.025)	-0.044* (-0.063, -0.025)

**OBC Household Heads**	0.009 (-0.037, 0.055)	-0.042 (-0.086, 0.001)	-0.014 (-0.054, 0.025)	0.021 (-0.014, 0.058)	0.002 (-0.010, 0.014)	0.003 (-0.008, 0.013)	0.003 (-0.037, 0.042)	0.044* (0.004, 0.083)	-0.030 (-0.069, 0.008)	0.002 (-0.026, 0.031)	0.001 (-0.003, 0.004)	-0.000 (-0.002, 0.002)	-0.012 (-0.028, 0.005)	-0.013 (-0.027, 0.000)	0.003 (-0.014, 0.020)	0.004 (-0.010, 0.018)
**Muslim Household Heads**	0.014 (-0.018, 0.045)	0.013 (-0.018, 0.045)	0.007 (-0.020, 0.033)	0.006 (-0.020, 0.032)	-0.003 (-0.011, 0.005)	-0.006 (-0.014, 0.001)	-0.002 (-0.029, 0.024)	0.052* (0.024, 0.080)	-0.021 (-0.047, 0.005)	-0.036* (-0.057, -0.015)	-0.002* (-0.004, 0.000)	-0.000 (-0.002, 0.001)	-0.005 (-0.016, 0.006)	-0.004 (-0.013, 0.006)	-0.007 (-0.019, 0.004)	0.008 (-0.002, 0.018)
**Female Household Heads**	0.000 (-0.047, 0.048)	0.077* (0.031, 0.122)	-0.033 (-0.073, 0.008)	-0.024 (-0.062, 0.013)	0.001 (-0.011, 0.014)	0.004 (-0.007, 0.015)	-0.028 (-0.068, 0.013)	-0.045* (-0.086, -0.004)	-0.044* (-0.084, -0.005)	-0.035* (-0.065, -0.005)	-0.004* (-0.007, -0.000)	-0.001 (-0.003, 0.001)	0.002 (-0.015, 0.019)	0.014* (0.000, 0.028)	-0.010 (-0.027, 0.008)	0.006 (-0.008, 0.021)
**Poor**	0.011 (-0.039, 0.061)	0.100* (0.041, 0.159)	0.017 (-0.026, 0.060)	0.021 (-0.028, 0.070)	0.006 (-0.007, 0.019)	0.024* (0.010, 0.038)	0.007 (-0.036, 0.050)	-0.006 (-0.059, 0.048)	-0.008 (-0.049, 0.034)	-0.022 (-0.061, 0.017)	-0.001 (-0.005, 0.002)	-0.001 (-0.003, 0.002)	-0.007 (-0.025, 0.011)	0.020* (0.001, 0.038)	-0.001 (-0.019, 0.018)	0.027* (0.008, 0.046)
**Households BPL with Ration Cards**	0.046 (-0.005, 0.097)	0.001 (-0.043, 0.045)	-0.036 (-0.080, 0.008)	0.020 (-0.016, 0.056)	-0.001 (-0.014, 0.012)	0.005 (-0.006, 0.015)	0.015 (-0.029, 0.059)	-0.005 (-0.044, 0.035)	-0.016 (-0.058, 0.027)	0.000 (-0.029, 0.029)	-0.004* (-0.007, -0.000)	-0.001 (-0.003, 0.001)	0.011 (-0.008, 0.029)	-0.009 (-0.023, 0.004)	0.000 (-0.019, 0.019)	-0.001 (-0.015, 0.013)
**Households with Improved Sanitation**	0.004 (-0.037, 0.045)	-0.084* (-0.133, -0.034)	0.009 (-0.026, 0.045)	-0.034 (-0.075, 0.007)	0.005 (-0.006, 0.015)	-0.005 (-0.017, 0.006)	0.020 (-0.015, 0.055)	-0.033 (-0.078, 0.011)	0.007 (-0.027, 0.041)	0.041* (0.008, 0.073)	0.001 (-0.002, 0.004)	0.000 (-0.002, 0.003)	0.005 (-0.010, 0.020)	-0.021* (-0.037, -0.006)	0.014 (-0.001, 0.029)	-0.026* (-0.042, -0.010)
**Households with Safe Drinking Water**	0.008 (-0.024, 0.040)	0.017 (-0.012, 0.046)	0.013 (-0.014, 0.041)	-0.020 (-0.044, 0.004)	-0.006 (-0.015, 0.002)	-0.002 (-0.009, 0.004)	-0.003 (-0.31, 0.024)	-0.010 (-0.035, 0.037)	-0.007 (-0.033, 0.020)	0.012 (-0.007, 0.031)	-0.002 (-0.004, 0.001)	-0.001 (-0.002, 0.000)	-0.011 (-0.022, 0.001)	-0.005 (-0.014, 0.004)	-0.000 (-0.012, 0.011)	-0.008 (-0.017, 0.002)
**Electrified Households**	-0.030 (-0.071, 0.011)	0.027 (-0.013, 0.068)	0.021 (-0.014, 0.056)	0.048* (0.015, 0.082)	0.012* (0.001, 0.022)	0.006 (-0.003, 0.016)	0.037* (0.002, 0.072)	-0.032 (-0.069, 0.004)	0.029 (-0.005, 0.064)	0.006 (-0.020, 0.033)	-0.002 (-0.004, 0.001)	-0.002 (-0.003, 0.000)	0.004 (-0.011, 0.018)	0.028* (0.015, 0.040)	-0.002 (-0.017, 0.013)	0.010 (-0.003, 0.023)
**Households using Solid Fuels**	0.007 (-0.040, 0.054)	0.035 (-0.005, 0.075)	0.035 (-0.006, 0.075)	-0.080* (-0.113, -0.047)	0.006 (-0.006, 0.018)	-0.013 (-0.022, -0.003)	0.025 (-0.015, 0.066)	0.001 (-0.035, 0.037)	0.031 (-0.008, 0.071)	0.017 (-0.009, 0.044)	-0.003 (-0.006, 0.001)	-0.003* (-0.005, -0.002)	0.016 (-0.001, 0.033)	0.003 (-0.010, 0.015)	0.014 (-0.003, 0.031)	-0.014* (-0.026, -0.001)
**Uneducated Household heads**	-0.007 (-0.051, 0.037)	-0.097* (-0.137, -0.058)	-0.019 (-0.056, 0.019)	-0.018 (-0.051, 0.014)	0.004 (-0.008, 0.015)	-0.007 (-0.017, 0.002)	0.026 (-0.012, 0.063)	0.022 (-0.014, 0.057)	0.025 (-0.012, 0.061)	0.048* (0.022, 0.074)	0.001 (-0.002, 0.004)	-0.003* (-0.004, -0.001)	0.007 (-0.008, 0.023)	-0.027* (-0.040, -0.015)	0.007 (-0.010, 0.023)	-0.028* (-0.041, -0.016)
**Household Heads with College Education**	0.010 (-0.024, 0.044)	0.029* (0.002, 0.056)	-0.032* (-0.061, -0.003)	-0.021 (-0.043, 0.046)	0.010* (0.001, 0.018)	-0.000 (-0.007, 0.006)	0.019 (-0.010, 0.047)	0.028* (0.003, 0.052)	-0.033((-0.061, -0.005)	-0.017 (-0.035, 0.001)	0.000 (-0.002, 0.003)	-0.003* (-0.005, -0.000)	0.016* (0.004, 0.028)	0.010* (0.002, 0.019)	0.006 (-0.006, 0.018)	0.001 (-0.008, 0.010)
**Mothers Married Young**	-0.004 (-0.064, 0.055)	0.026 (-0.029, 0.080)	0.007 (-0.044, 0.058)	0.001 (-0.043, 0.046)	0.019* (0.004, 0.034)	0.002 (-0.011, 0.015)	0.015 (-0.036, 0.066)	-0.015 (-0.064, 0.034)	-0.024 (-0.074, 0.025)	-0.015 (-0.050, 0.021)	0.000 (-0.004, 0.004)	-0.003* (-0.005, -0.000)	0.013 (-0.008, 0.034)	-0.013 (-0.029, 0.004)	0.015 (-0.006, 0.037)	-0.002 (-0.019, 0.015)
**Underweight Mothers**	0.011 (-0.037, 0.059)	0.005 (-0.039, 0.048)	-0.011 (-0.052, 0.030)	0.017 (-0.018, 0.053)	0.004 (-0.008, 0.016)	0.004 (-0.006, 0.015)	0.002 (-0.039, 0.043)	-0.034 (-0.072, 0.005)	0.033 (-0.007, 0.072)	-0.008 (-0.037, 0.020)	0.000 (-0.003, 0.003)	-0.003* (-0.004, -0.001)	-0.003 (-0.020, 0.014)	0.004 (-0.009, 0.017)	0.000 (-0.017, 0.018)	-0.011 (-0.025, 0.003)

S5: Evaluating associations between PM levels and different EJ dimensions after accounting for potential non-linearities

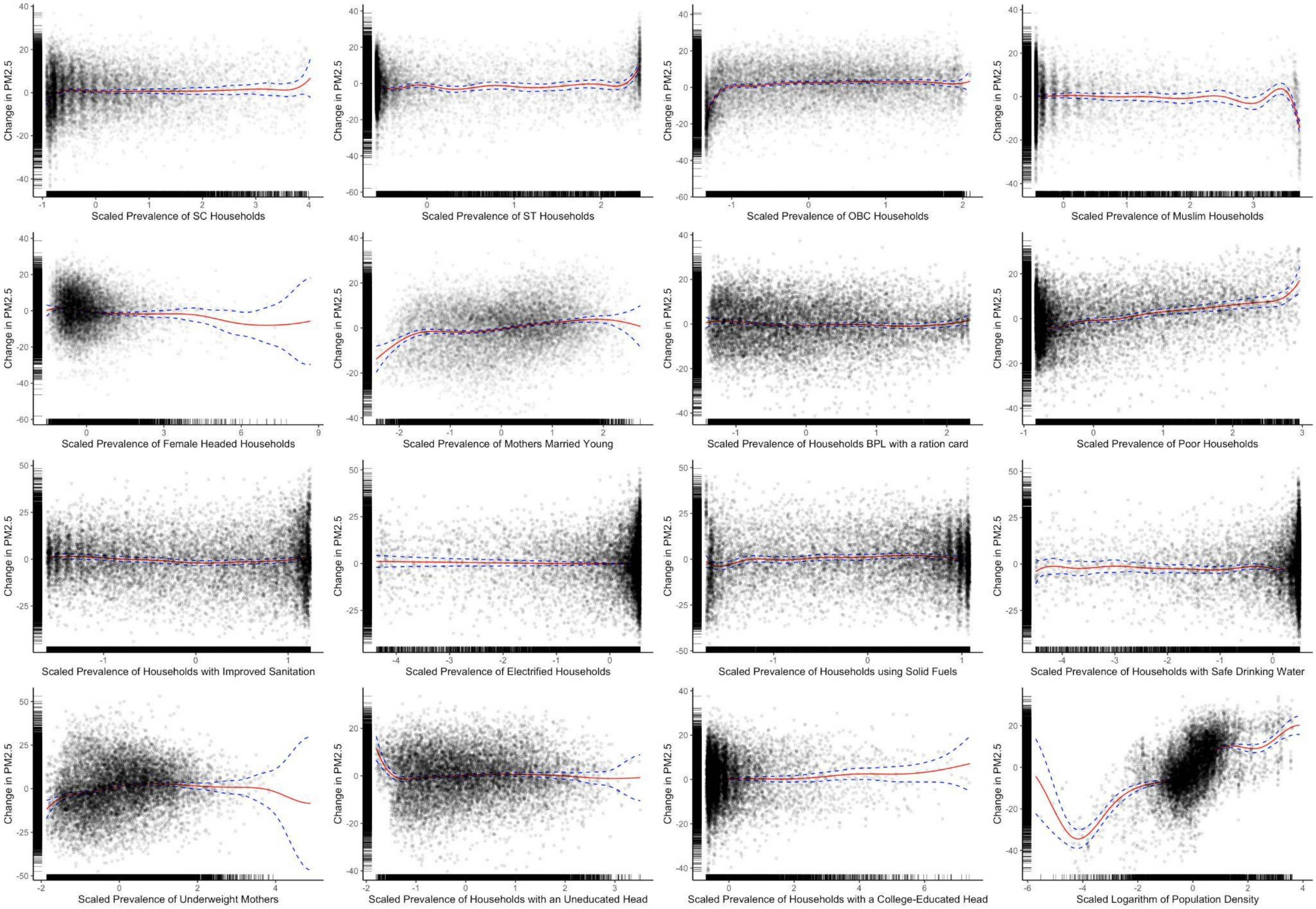

Figure S23*: Partial response plot (red line) and 95% CI (between the blue lines) for the association between anthropogenic $PM_{2.5}$ and the prevalence of A) SC households, B) ST households, C) OBC households, D) Muslim households, E) Households with a female head, F) Mothers married young < 18 y of age), G) Households BPL, H) Poor Households, I) Households with improved sanitation, J) Electrified households, K) Households using solid fuels, L) Households with safe drinking water, M) Underweight mothers, N) Household head without formal education, O) Household head with college-educated head, P) Population density in fully-adjusted models. We also display partial residual points and rug plots to provide readers with an understanding of the distribution of variables considered.*

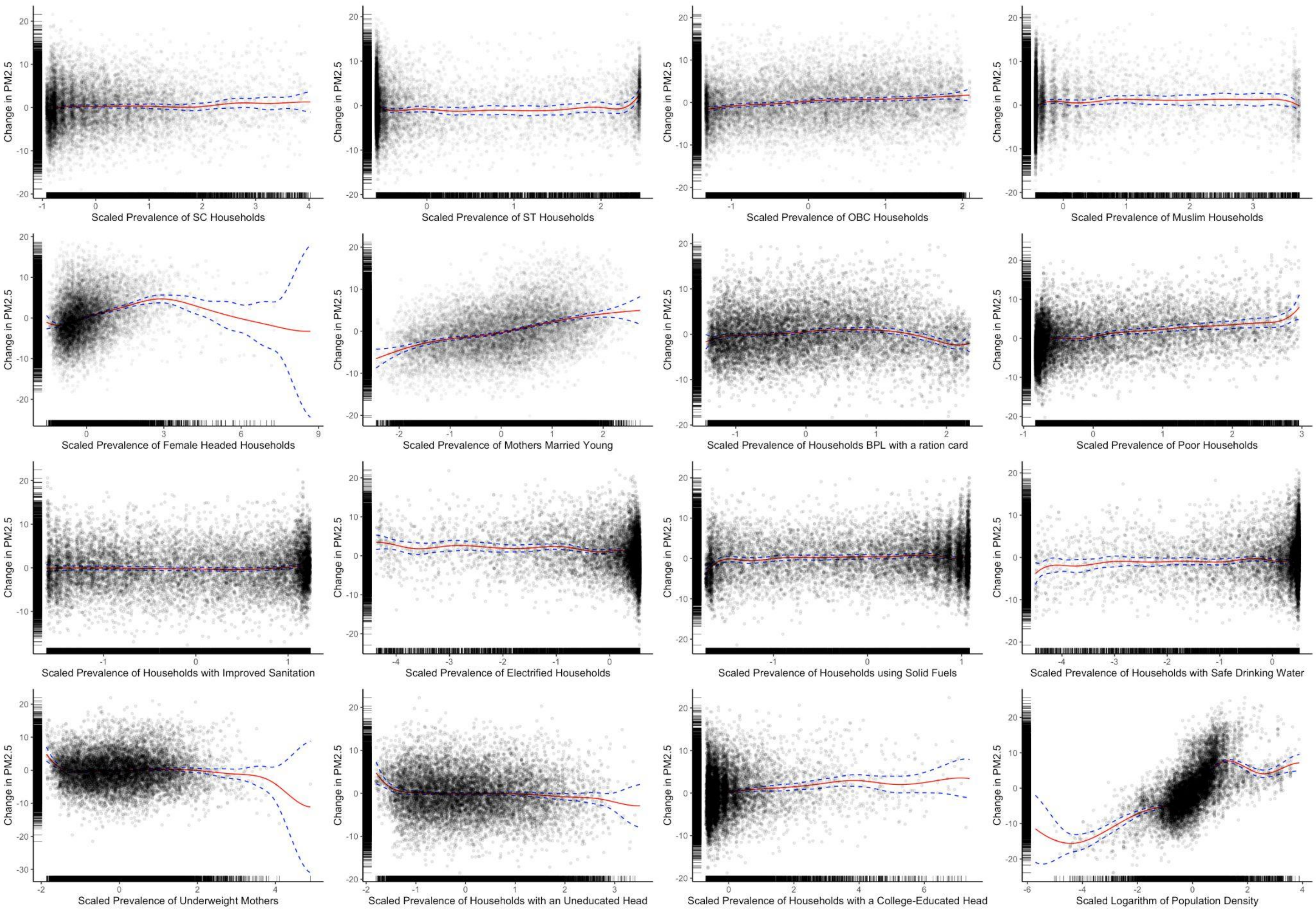

Figure S24*: Partial response plot (red line) and 95% CI (between the blue lines) for the association between industrial $PM_{2.5}$ and the prevalence of A) SC households, B) ST households, C) OBC households, D) Muslim households, E) Households with a female head, F) Mothers married young < 18 y of age), G) Households BPL, H) Poor Households, I) Households with improved sanitation, J) Electrified households, K) Households using solid fuels, L) Households with safe drinking water, M) Underweight mothers, N) Household head without formal education, O) Household head with college-educated head, P) Population density in fully-adjusted models. We also display partial residual points and rug plots to provide readers with an understanding of the distribution of variables considered.*

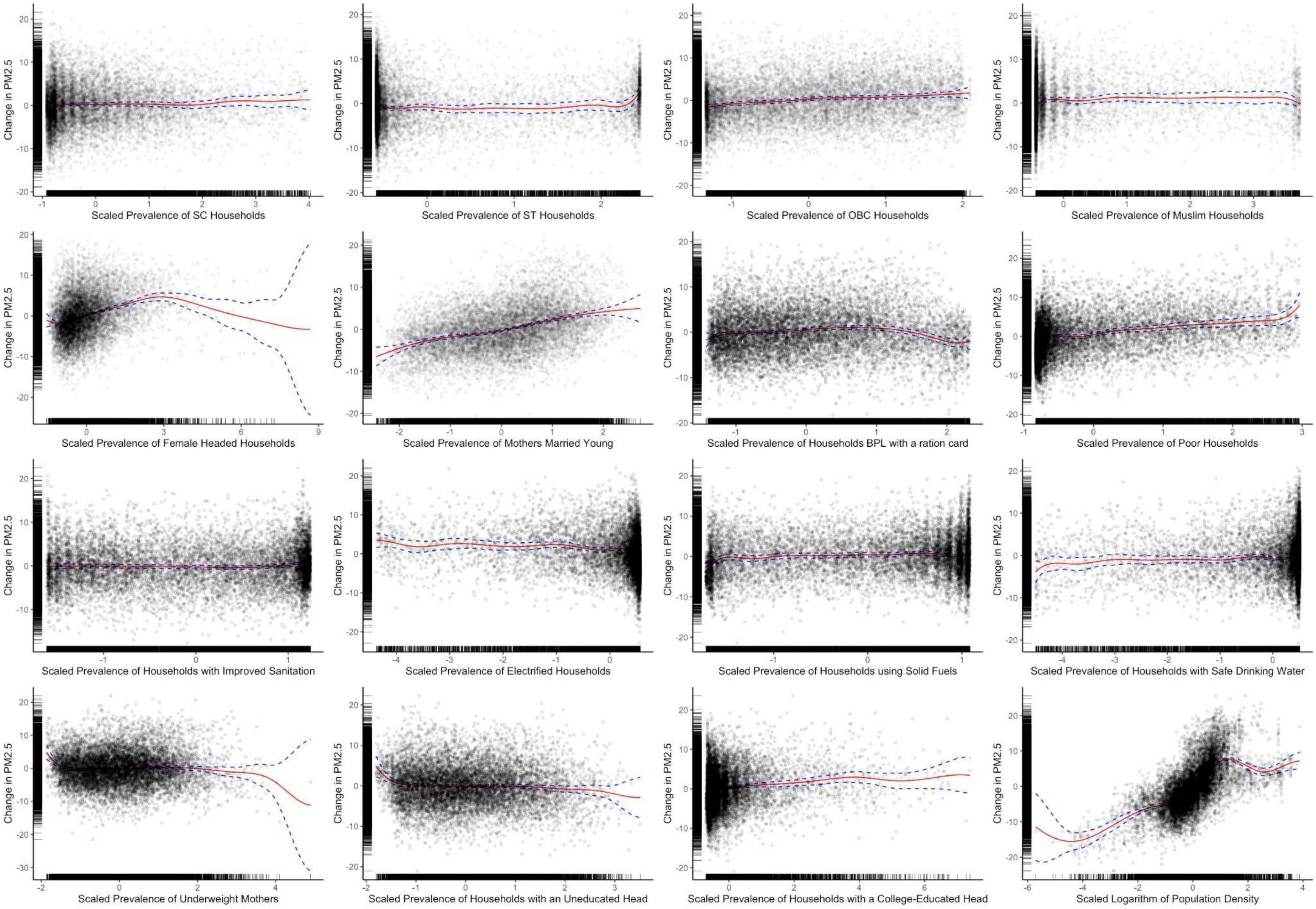

***Figure S25**: Partial response plot (red line) and 95% CI (between the blue lines) for the association between domestic burning $PM_{2.5}$ and the prevalence of A) SC households, B) ST households, C) OBC households, D) Muslim households, E) Households with a female head, F) Mothers married young < 18 y of age), G) Households BPL, H) Poor Households, I) Households with improved sanitation, J) Electrified households, K) Households using solid fuels, L) Households with safe drinking water, M) Underweight mothers, N) Household head without formal education, O) Household head with college-educated head, P) Population density in fully-adjusted models. We also display partial residual points and rug plots to provide readers with an understanding of the distribution of variables considered.*

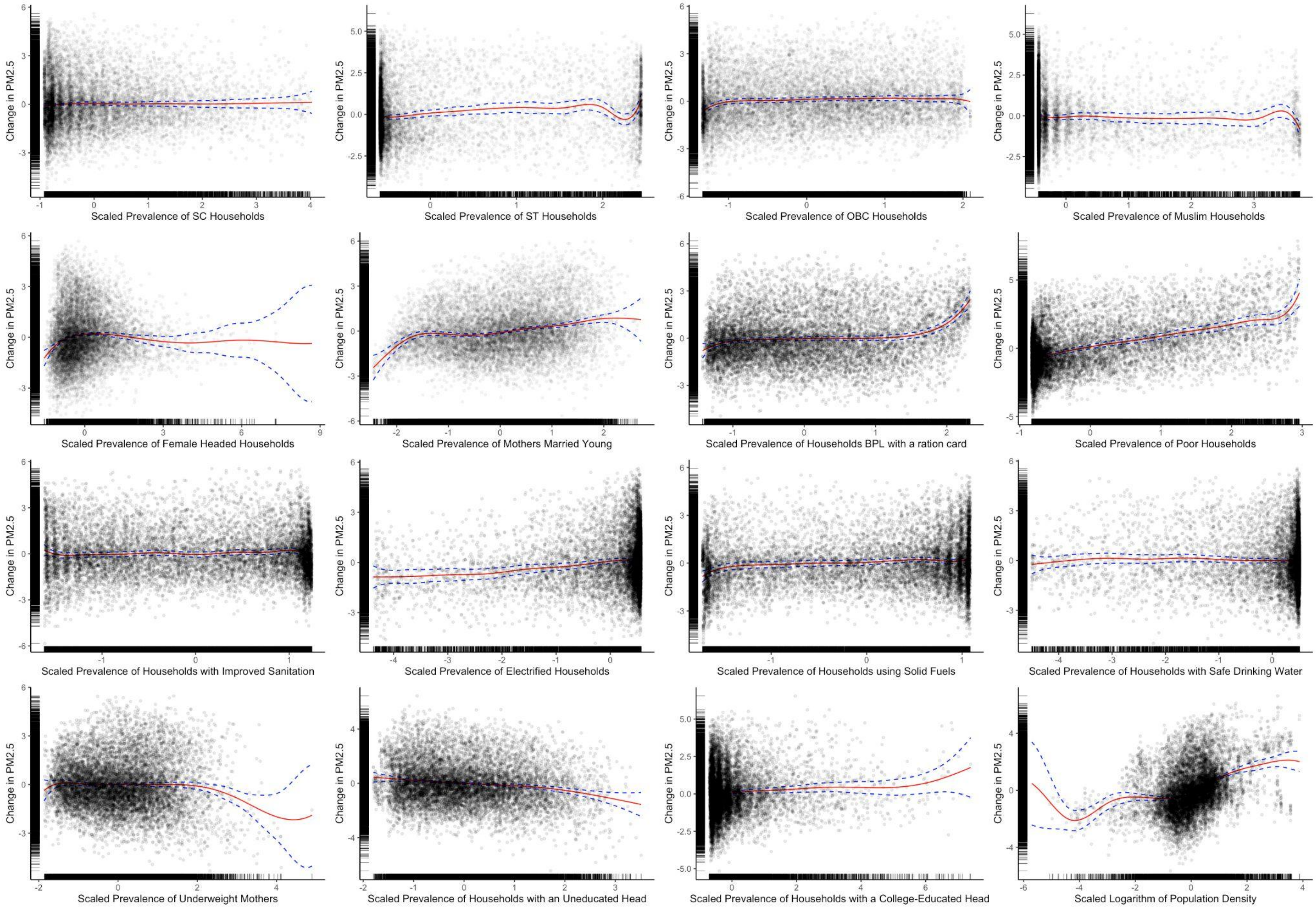

***Figure S26**: Partial response plot (red line) and 95% CI (between the blue lines) for the association between power $PM_{2.5}$ and the prevalence of A) SC households, B) ST households, C) OBC households, D) Muslim households, E) Households with a female head, F) Mothers married young < 18 y of age), G) Households BPL, H) Poor Households, I) Households with improved sanitation, J) Electrified households, K) Households using solid fuels, L) Households with safe drinking water, M) Underweight mothers, N) Household head without formal education, O) Household head with college-educated head, P) Population density in fully-adjusted models. We also display partial residual points and rug plots to provide readers with an understanding of the distribution of variables considered.*

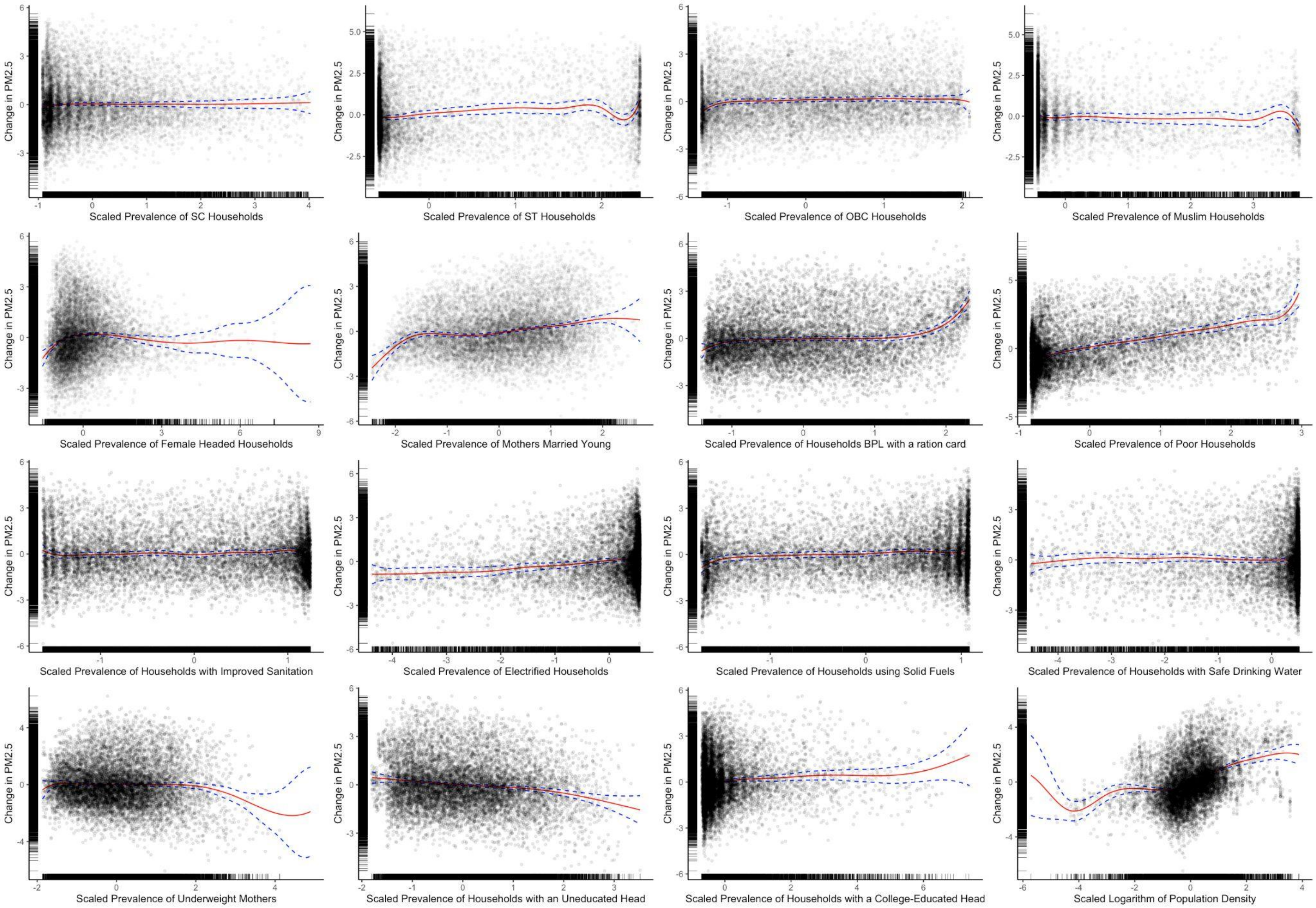

***Figure S27**: Partial response plot (red line) and 95% CI (between the blue lines) for the association between transport $PM_{2.5}$ and the prevalence of A) SC households, B) ST households, C) OBC households, D) Muslim households, E) Households with a female head, F) Mothers married young < 18 y of age), G) Households BPL, H) Poor Households, I) Households with improved sanitation, J) Electrified households, K) Households using solid fuels, L) Households with safe drinking water, M) Underweight mothers, N) Household head without formal education, O) Household head with college-educated head, P) Population density in fully-adjusted models. We also display partial residual points and rug plots to provide readers with an understanding of the distribution of variables considered.*

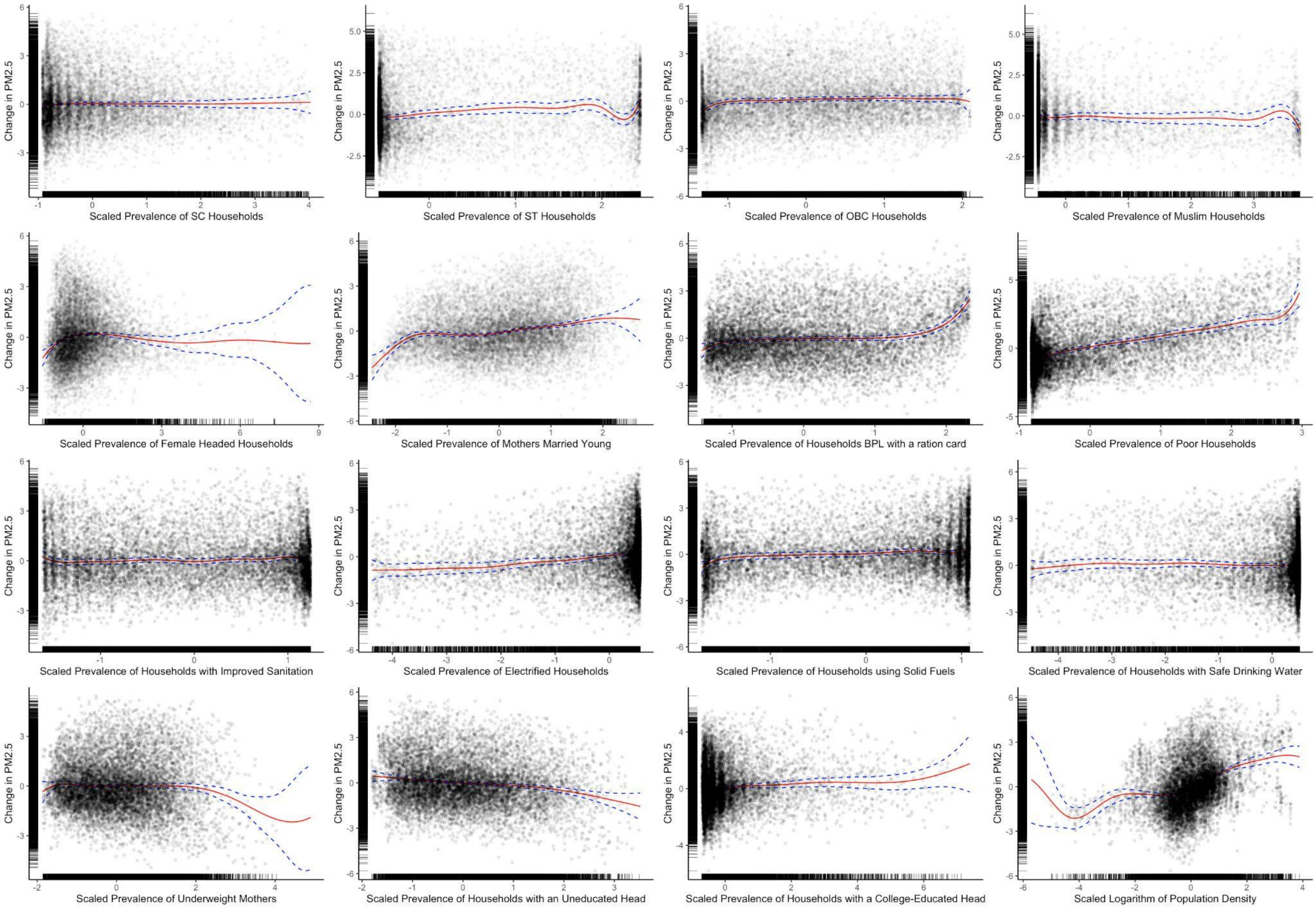

Figure S28*: Partial response plot (red line) and 95% CI (between the blue lines) for the association between road dust $PM_{2.5}$ and the prevalence of A) SC households, B) ST households, C) OBC households, D) Muslim households, E) Households with a female head, F) Mothers married young < 18 y of age), G) Households BPL, H) Poor Households, I) Households with improved sanitation, J) Electrified households, K) Households using solid fuels, L) Households with safe drinking water, M) Underweight mothers, N) Household head without formal education, O) Household head with college-educated head, P) Population density in fully-adjusted models. We also display partial residual points and rug plots to provide readers with an understanding of the distribution of variables considered.*

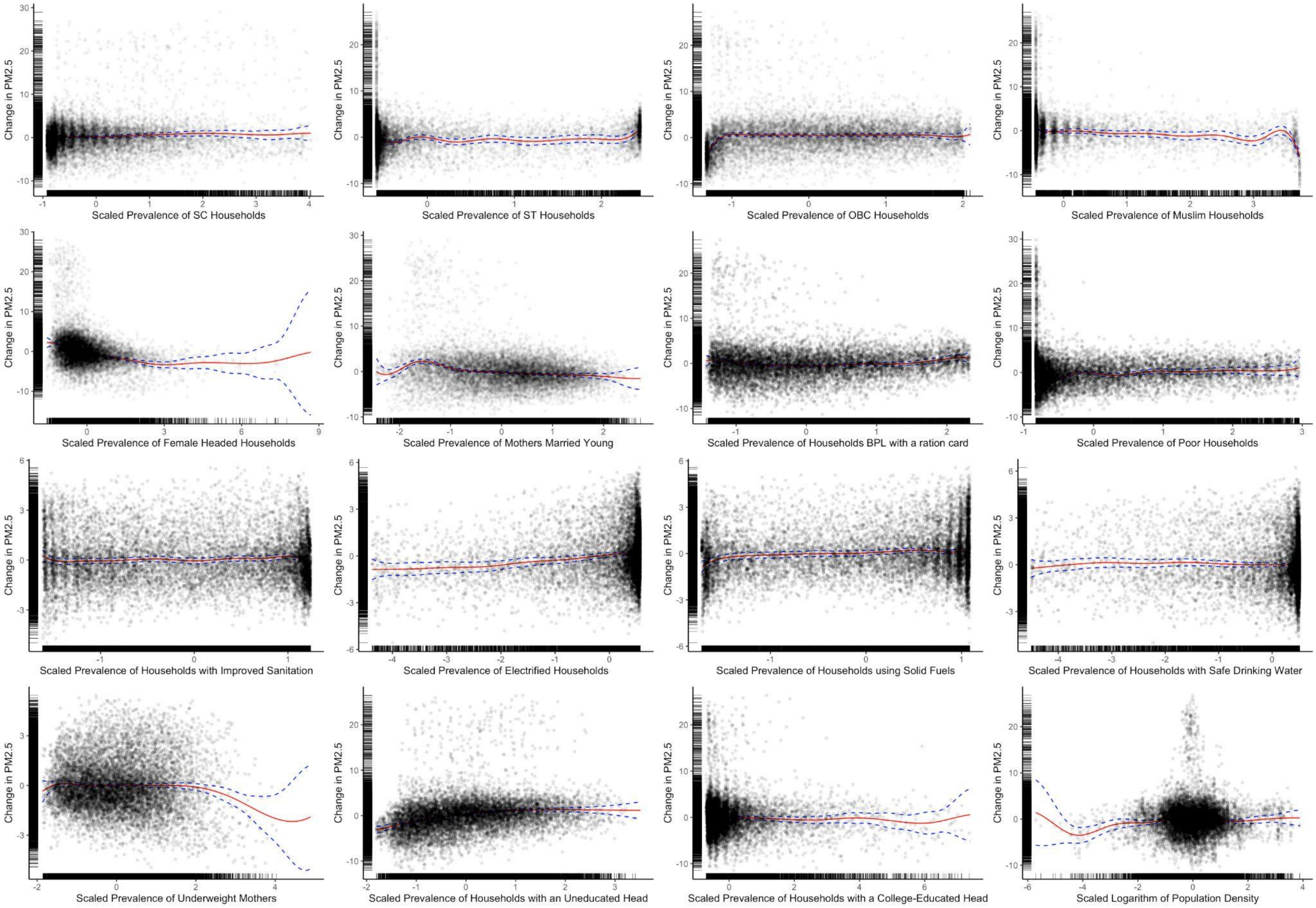

Figure S29*: Partial response plot (red line) and 95% CI (between the blue lines) for the association between agricultural residue burning $PM_{2.5}$ and the prevalence of A) SC households, B) ST households, C) OBC households, D) Muslim households, E) Households with a female head, F) Mothers married young < 18 y of age), G) Households BPL, H) Poor Households, I) Households with improved sanitation, J) Electrified households, K) Households using solid fuels, L) Households with safe drinking water, M) Underweight mothers, N) Household head without formal education, O) Household head with college-educated head, P) Population density in fully-adjusted models. We also display partial residual points and rug plots to provide readers with an understanding of the distribution of variables considered.*

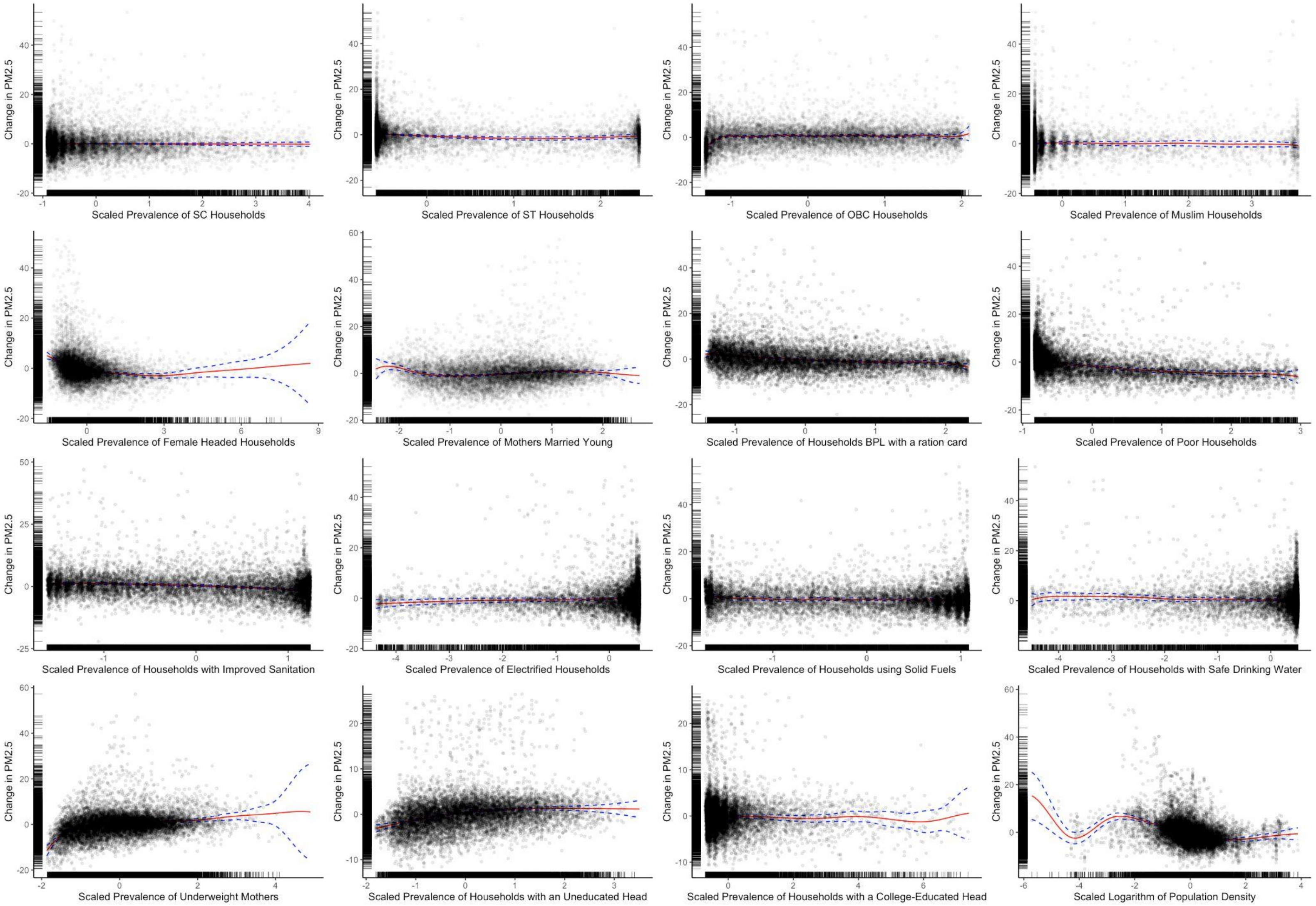

Figure S30*: Partial response plot (red line) and 95% CI (between the blue lines) for the association between international $PM_{2.5}$ and the prevalence of A) SC households, B) ST households, C) OBC households, D) Muslim households, E) Households with a female head, F) Mothers married young < 18 y of age), G) Households BPL, H) Poor Households, I) Households with improved sanitation, J) Electrified households, K) Households using solid fuels, L) Households with safe drinking water, M) Underweight mothers, N) Household head without formal education, O) Household head with college-educated head, P) Population density in fully-adjusted models. We also display partial residual points and rug plots to provide readers with an understanding of the distribution of variables considered.*

References

1. QGIS Development Team. *QGIS Geographic Information System*. (Open Source Geospatial Foundation, 2009).

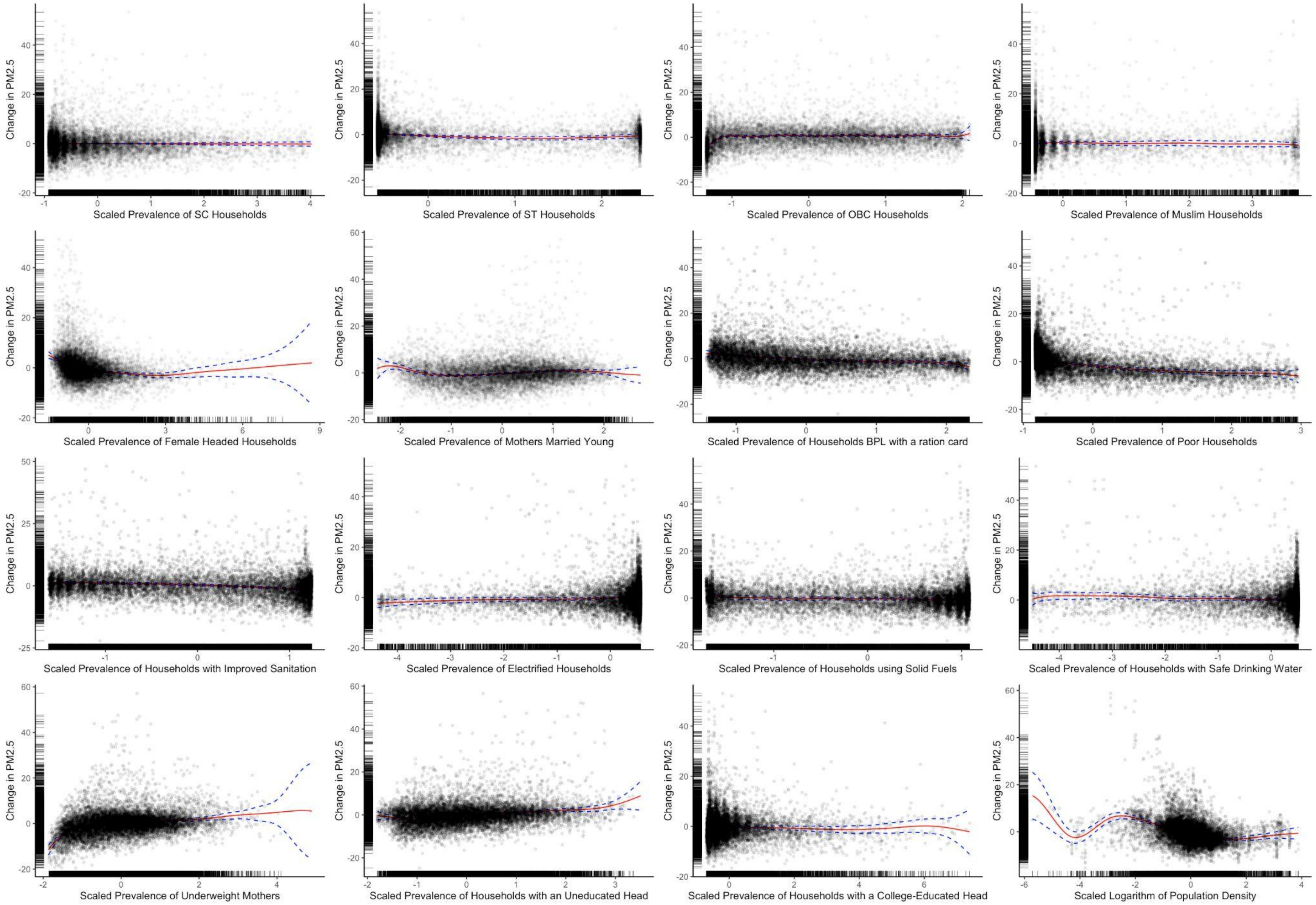

Figure S31*: Partial response plot (red line) and 95% CI (between the blue lines) for the association between other $PM_{2.5}$ and the prevalence of A) SC households, B) ST households, C) OBC households, D) Muslim households, E) Households with a female head, F) Mothers married young < 18 y of age), G) Households BPL, H) Poor Households, I) Households with improved sanitation, J) Electrified households, K) Households using solid fuels, L) Households with safe drinking water, M) Underweight mothers, N) Household head without formal education, O) Household head with college-educated head, P) Population density in fully-adjusted models. We also display partial residual points and rug plots to provide readers with an understanding of the distribution of variables considered.*
